# Supplementary material for: Strain-level detection of Fusobacterium nucleatum in colorectal cancer specimens by targeting the CRISPR–Cas region
Source: Microbiol Spectr. 2023 Oct 11;11(6):e05123-22. doi: 10.1128/spectrum.05123-22 (PMC10714804; doi:10.1128/spectrum.05123-22)
Supplement: Supplemental figures and tables — Fig. S1 to S15; Tables S1 to S6. [file spectrum.05123-22-s0001.pdf]

Supplemental Materials

Strain-level detection of *Fusobacterium nucleatum* in colorectal cancer specimens by targeting the CRISPR–Cas region

Yumi Shimomura, Yutaka Sugi, Aiko Kume, Wataru Tanaka, Tsutomu Yoshihara, Tetsuya Matsuura, Yasuhiko Komiya, Yusuke Ogata, Wataru Suda, Masahira Hattori, Takuma Higurashi, Atsushi Nakajima, Mitsuharu Matsumoto

F. nucleatum subsp. *nucleatum* JCM8532^{T}, Type I-B2_5'-end

```
ATTTCTTATA AAATTTATTG TCTACCTCTG ATAATGTAAA ATTACTGGAA GATTGACAAA AGTTATAAAT GTGTTTATTT ATAAATAAAT TTAAGAAAAT AAAAAATTAT
AAGAAATTAA AGTTTTTTAT TTTTTAGTAA AAAAACTTAC ATTGACAAAA AGATATAAAA AAAGTATTAT GTAGGAGTAG TTTTTTTAGC TAGCTATTTA TGTATTTCTA
TATTAGAATT TAAATGAGTT TTGAGTTAAT TCATTGACAT AATGTATTTT TGAATTTATG TATTTCTATA TTAGAATTTA AATCTACTTC CAACTAATTT ATTATTTGAA
TCAACAGATT TATGTATTTC TATATTAGAA TTTAAATAAG TATTACAGAT GTTTAAGTTA AAAGATTCAA GTATTCATTT ATGTATTTCT ATATTAGAAT TTAAATAAAT
CTGACAGTGG TTATAATTTC AAAGAAGTTT TTTAATTTAT GTATTTCTAT ATTAGAATTT AAATGTAACT ATTCCAGGAT TAAATGAAGT CAATAATCTA ATTTATGTAT
TTCTATATTA GAATTTAAAT GTAGCTTCAT TAGCTAATTC AAATAAAAAA TCTCTTATTT ATGTATTTCT
```

F. nucleatum subsp. *nucleatum* JCM8532^{T}, Type I-B2, 3'-end (Reverse complement)

```
CAACTGTCAG ATCTTTTACA TATCAGTTTT GTTAACTCAT TATATCATAC TTTTTGAAGA ATTCGATATT ATTTTTATAT AATTAAATTG AACATGTATA AATATCTAAG
TGAGGGATTT AAATTATGTA AAGTATATAA AAGTAGTAGT GGTCTATGAA GATAGTAAAC AATATCATTT TTTAAGAATA CAATAAGAAA ATTTTAAATA CATTTCATAA
AAAACTTCAA TATATATGAG TAAAAATATC CTATTAAAGT TTCAAATGAA GATTTTTTAT TATAGGAACT TTATGAAGGA ACTTTATGAA ATAAAAATAG TTTATAAAAA
TGTAAAAAAG TAAAAGCCAA TTAAATGGAT TAATTTTTAT TATGTTAAGT ATATCTTTAT AAAGTAAACT AAGAATTAAG TATCTTTAAA GTGATAAAAA AAATAAAAAG
ATAAATTCCA ACTAAAATAT TAGAATAATG AACATAATTC TTTTAAAAAT AGAAAAAGAA AGTTACTGAA TATTAAAATG ATAAAATTTT ATTAAAAATT GAGTGAATTT
TACAATAAAA TTATTGCATT TAATCGTAAT TGGTGTTAAT ATATACTTAA TAAATTTAAA AGATAAATTT
```

F. nucleatum subsp. *polymorphum* JCM12990^{T}, Type I-B2, 5'-end

```
TTTCTTATAA AATTTATTGT CTACCTCTCA TAATGTAAAA TTACTGGAAG ATTGACAAAA GTCAAAAAAG TCTTTATTTA TAAATAAATT TAAGAAAATA AAAAAAATTT
AAGAAATTAA ATTTTTTTAT TTTTTACTAA AAAACTTATA TTGACAAAAA GATATTAAAA AAGTATTATA TGGGAGTAGT TTTTTTAGGT AGCTATTTAT GTATTTCTAT
ATTAGAATTT AAATAAGGCT TGAACACAGA TTAACTAAGA AAATTATAAA AAATTTATGT ATTTCTATAT TAGAATTTAA ATGGAGAGAT AGTATTGACA CATTAGGAGT
TGAGGTAATT TATGTATTTC TATATTAGAA TTTAAATAAA TGGTATTTGT AAAGTTGATT TCTTTATACT CTTAATTTAT GTATTTCTAT ATTAGAATTT AAATCAACTT
TAAAAATTAT TGGATGTAAG GAGTTGTATT CATTTATGTA TTTCTATATT AGAATTTAAA TTCTAATGCT AAACTGTTCT TTTTTAATGG ATTTATTCAT TTATGTATTT
CTATATTAGA ATTTAAATGA TAAAGCTTTA ATTTTAACAG CAGGAGTAGA CATCATTTAT GTATTTCTAT
```

F. nucleatum subsp. *polymorphum* JCM12990^{T}, Type I-B2, 3'-end (Reverse complement)

```
TTAATTTATG TATTTCTATA TTAGAATTTA AATGGAACTG CTGCTTTAGG TGTTAAACTT CCTTCATAAA TTTATGTATT TCTATATTAG AATTTAAATG TTTTATATAG
TGAGTTTGAG AGAGAGTTGA CTGAAATTTA TGTATTTCTA TATTAGAATT TAAAGTGAAT TTCTTTAAAA TTTAATGTAT AATTCAAGTA TCAATTTATG TATTTCTATA
TTAGAAAAAA ATACAGAAGA AAGGGATTGA AAAATAAAGA CTTAATGCTG TTATATAACA AAAGAATAAA TAAAAAATTA AAAGGATTAA CTCTAGTTTT TTATATAAGT
TAATCCTTTT TAATAAGTTA AATTAAATTT ATTCAATTTT TTAAGGGGAG TACATTTTTT ATTATGTTAA GTATAGCTTT ATAAAGTAAA CTAAGAATTA AGTATTCATA
AAGTAACAGA AAAATTAAAA AAACGAAATT CAACTAAAAT ATTAATATAA TGAACACAAA AATTTAAGAT AGAAAATAAA AATTATCAAT TATTAAATTA ATAAAATTTT
ATTAAAAATT AAGTGAATTT CACAATAAAA ATATTGCATT TAATCGTAAT TGGTGTTAAT ATATACTTAA TAAATTTGAA AGATAAATT
```

F. nucleatum subsp. *vincentii* JCM11023^{T}, Type II-A, 5'-end

```
TGATCTACCC TCAGTAACAA ATAGTGATTT AAAAGAATAT AGAAAATTTA GAAAGTTTTT AATAGAAAAT GGATTTTCAA TGCTACAAGA ATCAGTATAT TCAAAGCTAC
TTTTACATAA TACAGCTTCA ATCAATATGG TAGATAAATT ACAAAAGAAT AAACCAGAAA AGGGGTCTGT TTGTATGTTA ATAATAACGG AAAAACAGTA TCAAAAAATG
ATTCTTTTAA TTGGGGAGTT AAAAGGGACA TTTTTAGAAA CAGATGAAAG GCTTGTGGTT TTATGACATT TCAGTATAAA GGTTTTAATT TCAAAATAGA TTTTGAAGAA
AAAAATATTT TTTCTTTAAT AGTAGAAAAT AAAAGAGCAT ATAGAAAAAT TATAGAGGAT TTAGTAAATA ACTCAAATAT TGAAGATGGG GATATTATTT TATCTAAGAA
TAATAAATTA GTTATACCAG AAAAAGAAAT TTTTATATTT TCAGATTATT TTAACTTTGA TATAAACAAA TTTGTTTTAA ATAAGTATTA TAAGGAATTA AAAAATTTAA
GTGAAAATGA GTTTTTAAAT GAAACTTTAG AAATTAAAGA AATTTTAAAA GATTATATTA ATAAATTAA
```

F. nucleatum subsp. *vincentii* JCM11023^{T}, Type II-A, 3'-end (Reverse complement)

```
AATTTTAAAT AATATCATAT ATTTTTTACT CCTTTAAAAT ATATTAGTAA TCTTCTAAAT GTTCTAATTC AAATTCAAGG TCAAATAATT TTAATTCTAA TTGAGCAACT
CTTCTCCTCA ATTATTTTAT AATATTTTTT ACAAAAAAAG AAGCTATTAT TTCAATAACT TCTTCAATCT TTATTATAAA TAAAATTTTA TTTGAATTCT TTTTTTATTT
CTGCTATCCA ATCTCCAATC CTTTTTGGAG TTAGGTTAGG TTGATTTTCT TCATCAAGAG CAAGTCCAAT AAATTTATCA TCTTTCACAA TACTTGTTTC TTCATAGTGA
TATCCATCAG TGCTTGTAAA TCCAACAACC TTACCACCTT TTTTAATAAC AATATCATAT AAATACTTTA TTCCACCACA GAAAGATTCT CCAAAAGCGA ATTGATTTCC
AAGCCCAACA AGTCCAACTA CTTTACCTGT AAAATCAATT TCTTCTAATT TTTTTAAATT ATTCATCCAA GCTGCATGAG CTTCTCCAAC TTGATATGTG GGTGTAACAA
AAATAAGATT TTCAAAATTT TCTATTTCTT TAACACCATT TTTGACATTA AAAGTTTTAA AATCATCTTT
```

F. nucleatum subsp. *animalis* JCM11025^{T}, Type III-A, 5'-end

```
AAGAATAGTA GAATATCAAA TTATGTTGCT AATTAAAGCA TTAGAAAGTA ATAATGCAGA TATATATAAG CCAGTTTTAA TTAGGTGATA TTATGGATAA TTGGGATTTT
TTAGATGAAG ATTTTGAAAA GGAAATTTTT GAAGATAACT TCACAGTTAT TATAATATAT GATATTATAT CCAATAAGAG AAGAACACAA TTATCCAAAC TTTTGAGTGC
ATTTGGATTT AGAATTCAAA AATCAGCATT TGAATGCCTT TTAACAAGAG AAAAATATAA ATTACTTATT GAAAAAATTG ATAGATATGC AAAACCTGAA GATTTAATAA
GAATTTATCG TTTAAATCAA AATGTAGTAA CTCAAATATA TGGAGAAAAA TTAGAAAATG AAAATGAAAT GTATTACTTT TTTTAATCAA AAATAGACGA TATGCTAAAA
ATTGGAGGTC TGATATGATT TTTTTAAAAA CAAATAATAA ACTTAAAAAA ATTAAAAAGT CATACGATAA TAGCTTTATA AACTATTACA AACAAGGTGT TTTTTGACCA
TAGATAAGAG
```

F. nucleatum subsp. *animalis* JCM11025^{T}, Type III-A, 3'-end (Reverse complement)

```
TAAGAGATAA TGTAACCTCA CAAGGGGACA GAAATTAAAG ATATAGTCTT CTATGAAAAG TAAATATCAT AGAGGGCTAT ATTTATTTGA ATAAAGATAT AGAGAAAATA
TAACTTTGTA AAGAGATAGA AAGTCATTTG AATTGCAAAT TGATGTTGGA GAAAATTAAA GATTAGAAAA AATAAAATTA AAGCAGGAAC GAAAATTTTG AAAAAATTCT
TTTGATATAG TATAACATTA AACAAAATAA AGAAGAATTA CAACTTCAAG AGGAGACAGA AATTCATTCT ATTGACAAGA AATCAATATC TTTTTATAAT ATCAGTATGA
AGAAATAAAA TTCTGAAAAA AGATAAAAAG TATAAAGATG TTATGATATT CTAATATTCA ACTAAGAATT TCTCACAATT GAAATTTTTA TAGAAAAAAT AAAAACTATT
TAAGGAGGGA TTTTATGAAA AAAACACTAT TTTCTATATT GCTTGTAGGA ATTTGTATGA CTGGAGTAGC AAATGCAAAA GAAAAAAATC CAATTCTTTT AAAACAAGTA
TACAAAAAAG AAGAATTAAT
```

Supplementary Figure S1. The DNA sequences of the PCR product of the target CRISPR-associated regions in *F. nucleatum* type strains

Approximately 500–600 bases (shown here) were used for analysis. The reverse sequences are shown as the reverse complementary sequences. The repeat sequences are presented in red. The sequences of these PCR products are 100% identical to the original genomic sequences (Supplementary Table S4).

Method: AP-PCR, Sample: Isolates

Patient D (Primer: D8635)

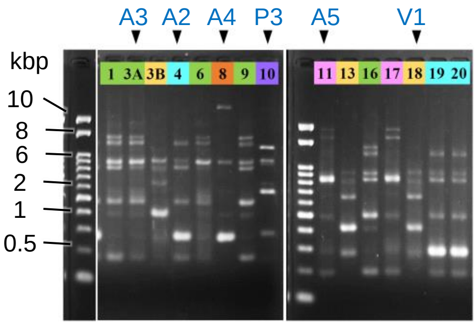

Strains	AP-PCR pattern	Representative strains
S1	A3	
S3A	A3	○
S3B	V1	
S4	A2	○
S6	A3	
S8	A4	○
S9	A3	
S10	P3	○
S11	A5	○
S13	V1	
S16	A3	
S17	A5	
S18	V1	○
S19	A2	
S20	A2	

Patient E (Primer: D8635)

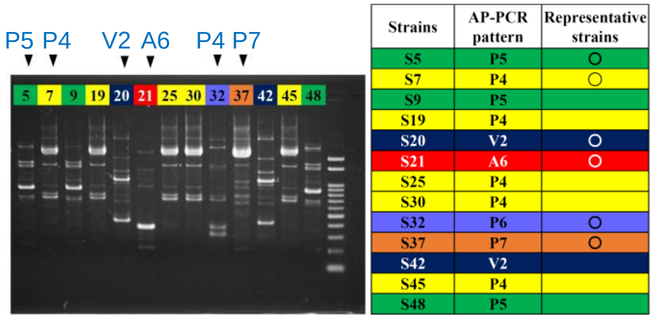

Strains	AP-PCR pattern	Representative strains
S5	P5	○
S7	P4	○
S9	P5	
S19	P4	
S20	V2	○
S21	A6	○
S25	P4	
S30	P4	
S32	P6	○
S37	P7	○
S42	V2	
S45	P4	
S48	P5	

Patient F (Primer: D11344)

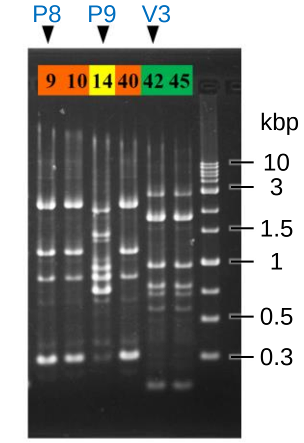

Strains	AP-PCR pattern	Representative strains
S9	P8	○
S10	P8	
S14	P9	○
S40	P8	
S42	V3	○
S45	V3	

Patient G (Primer: D8635)

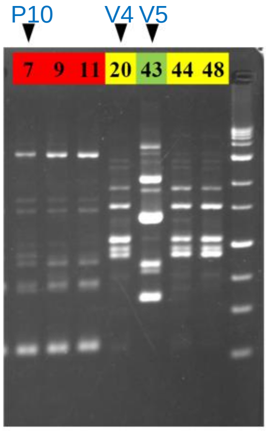

Strains	AP-PCR pattern	Representative strains
S7	P10	○
S9	P10	
S11	P10	
S20	V4	○
S43	V5	○
S44	V4	
S48	V4	

Patient M (Primer: D11344)

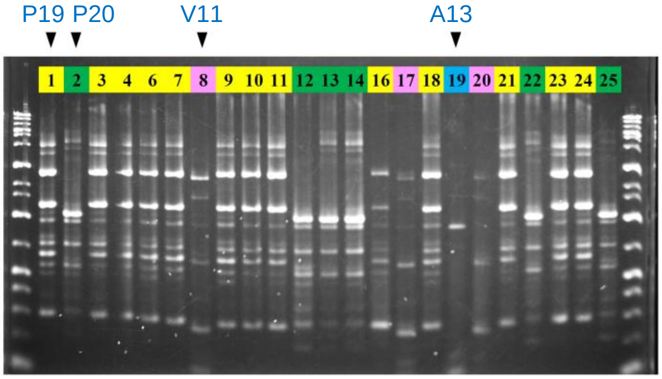

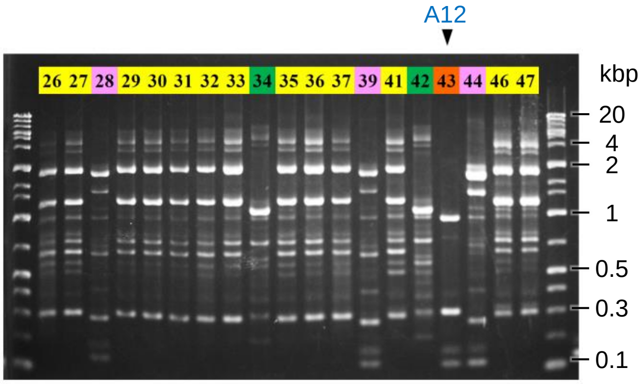

Strains	AP-PCR pattern	Representative strains
S1	P19	○
S2	P20	○
S3	P19	
S4	P19	
S6	P19	
S7	P19	
S8	V11	○
S9	P19	
S10	P19	
S11	P19	
S12	P20	
S13	P20	
S14	P20	
S16	P19	
S17	V11	
S18	P19	
S19	A13	○
S20	V11	
S21	P19	
S22	P20	
S23	P19	
S24	P19	
S25	P20	
S26	P19	
S27	P19	
S28	V11	
S29	P19	
S30	P19	
S31	P19	
S32	P19	
S33	P19	
S34	P20	
S35	P19	
S36	P19	
S37	P19	
S39	V11	
S41	P19	
S42	P20	
S43	A12	○
S44	V11	
S46	P19	
S47	P19	

Supplementary Figure S2. The *F. nucleatum* strains isolated from the saliva of CRC patients used in this study.
The AP-PCR pattern of the isolates derived from the saliva of CRC patients with isolated *F. nucleatum* from both CRC biopsy and saliva (Patient D, E, F, G, and M) in our previous study (Gut 68: 1335-7, 2019). The target CRISPR-associated regions of the representative strains (marked with arrows and the corresponding strain names) were analyzed by PCR.

ID and strain name of isolates derived from Patient F

Lane	Isolate ID	Bacterial name	Strain
1	16-315-A-C4	*F. nucleatum* subsp. *vincentii*	V3
2	16-315-A-C8	*F. nucleatum* subsp. *nucleatum*	N1
3	16-315-A-C9	*F. nucleatum* subsp. *nucleatum*	N1
4	16-315-A-C10	*F. nucleatum* subsp. *nucleatum*	N1
5	16-315-A-C15	*F. nucleatum* subsp. *nucleatum*	N1
6	16-315-A-C16	*F. nucleatum* subsp. *nucleatum*	N1
7	16-315-A-C17	*F. nucleatum* subsp. *nucleatum*	N1
8	16-315-A-C23	*F. nucleatum* subsp. *nucleatum*	N1
9	16-315-A-C29	*F. nucleatum* subsp. *nucleatum*	N1
10	16-315-A-C33	*F. nucleatum* subsp. *nucleatum*	N1
11	16-315-A-C36	*F. nucleatum* subsp. *vincentii*	V3
12	16-315-A-C37	*F. nucleatum* subsp. *nucleatum*	N1
13	16-315-A-C38	*F. nucleatum* subsp. *nucleatum*	N1
14	16-315-A-S9	*F. nucleatum* subsp. *polymorphum*	P8
15	16-315-A-S10	*F. nucleatum* subsp. *polymorphum*	P8
16	16-315-A-S14	*F. nucleatum* subsp. *polymorphum*	P9
17	16-315-A-S40	*F. nucleatum* subsp. *polymorphum*	P8
18	16-315-A-S42	*F. nucleatum* subsp. *vincentii*	V3
19	16-315-A-S45	*F. nucleatum* subsp. *vincentii*	V3

First 13 isolates (lane 1 to 13) were obtained from colorectal cancer, last 6 isolates (lane 14-19) were obtained from saliva

Method: AP-PCR or *F. nucleatum*-genotyping PCR;
Sample: Isolates

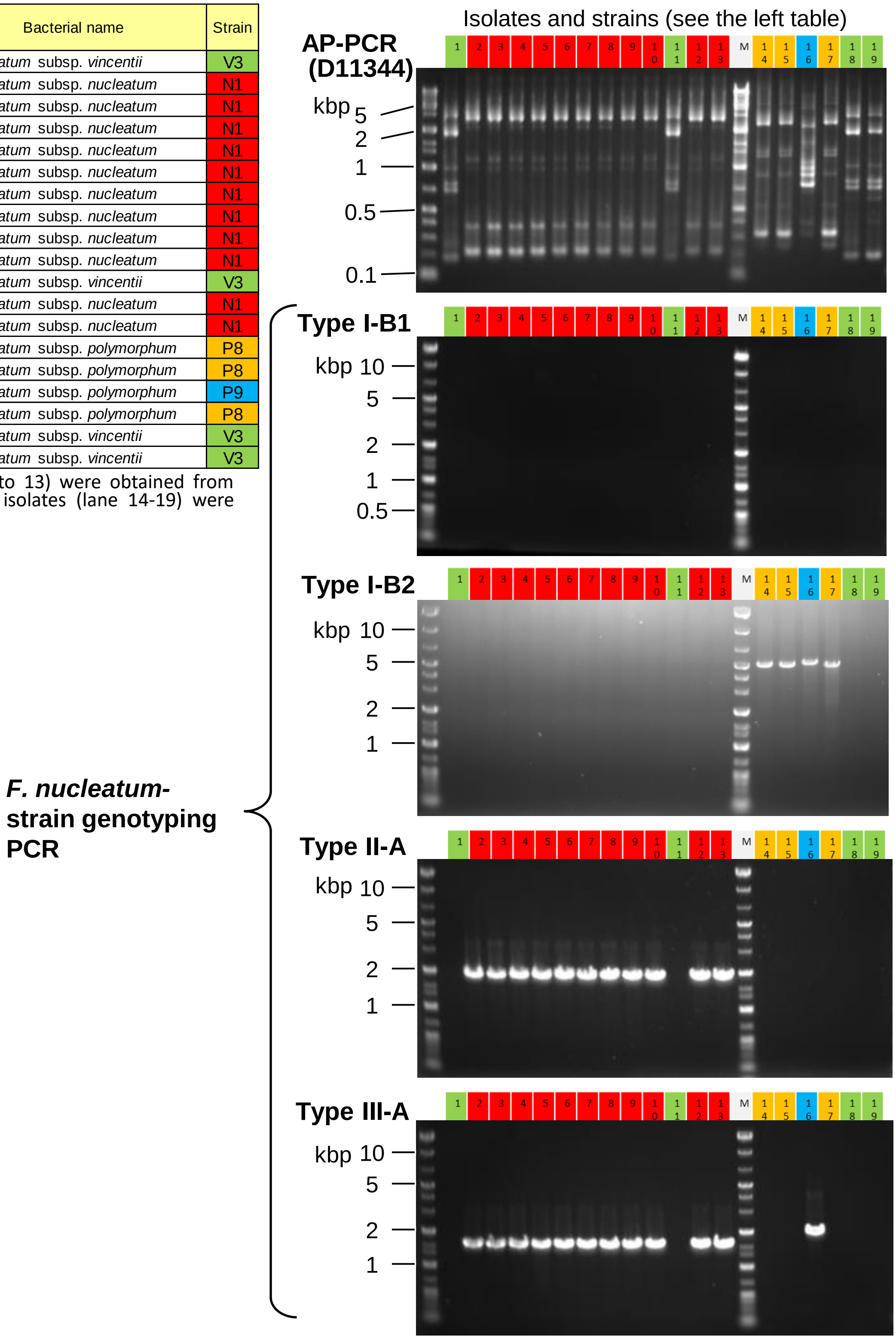

Supplementary Figure S3. *F. nucleatum*-strain typing PCR products of all the isolates derived from the saliva of CRC patients in our previous study (Gut 68: 1335-7, 2019).
All isolates derived from the saliva of one CRC patient (Patient F) (19 isolates classified as four strains in the Table provided [top left]) were analyzed by Fn-strain typing PCR. AP-PCR was performed using the D11344 primer.

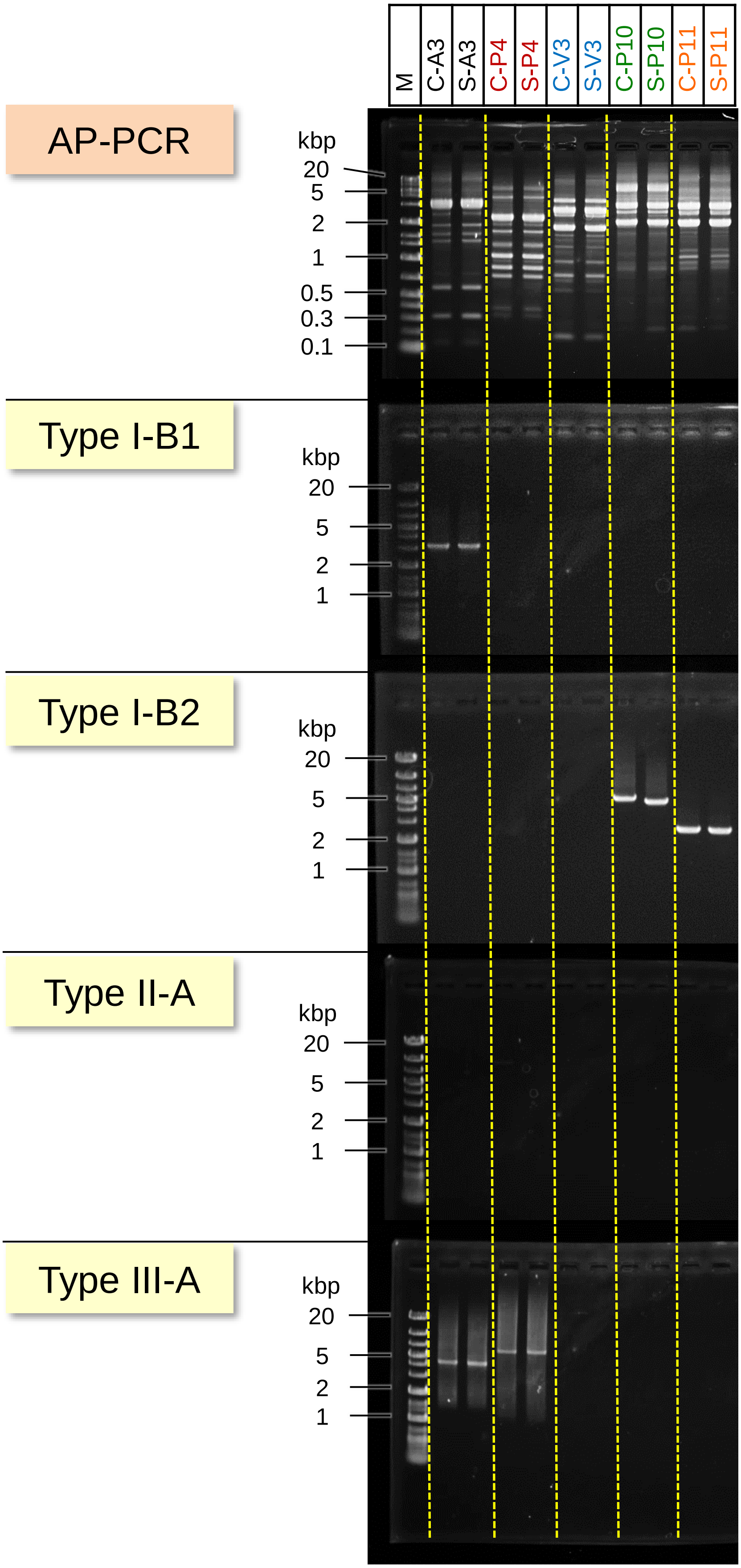

Supplementary Figure S4. *Fn*-strain genotyping PCR products of five pairs of isolates derived from saliva or CRC, which were identified as strains originated from identical strain by whole-genome sequencing. Isolates with the same color are those that have been detected as the same strain by AP-PCR. M:Maker of gene size

Method: *F. nucleatum*-genotyping PCR; Sample: Isolates or saliva

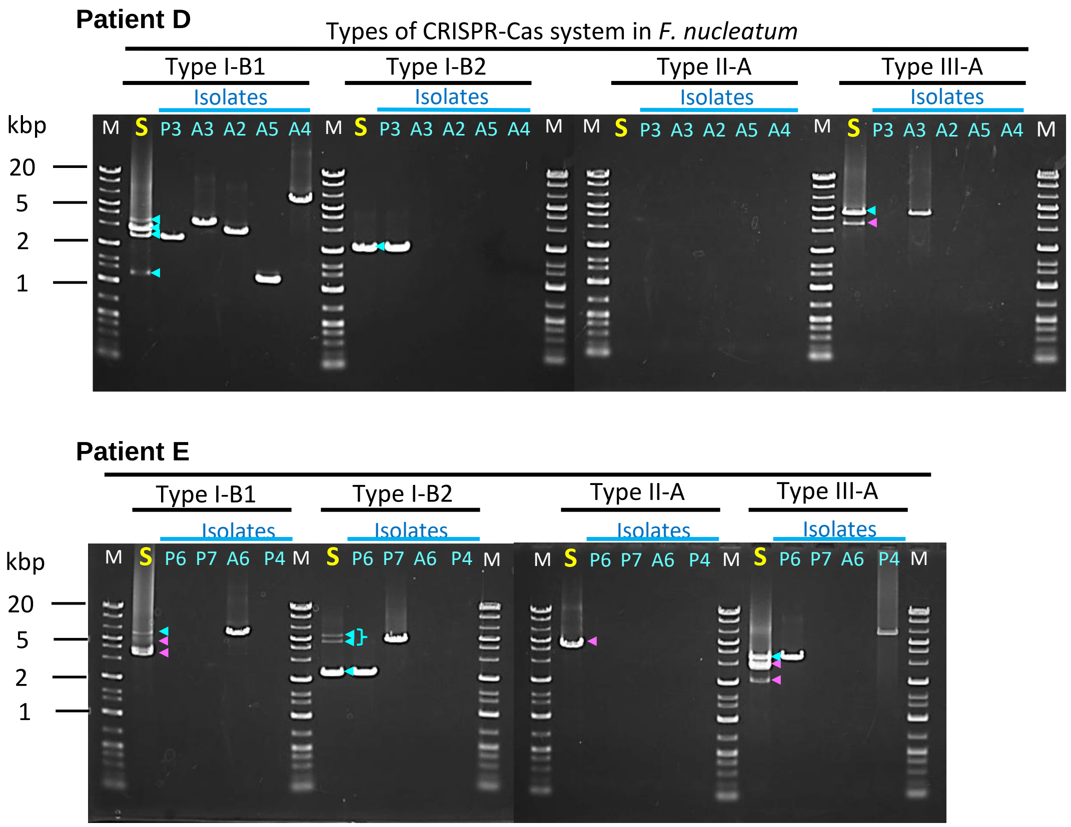

Supplementary Figure S5. Detection of strains from genomic DNA of saliva derived from CRC patients.
S: saliva; others: IDs of strains that isolated from the same saliva in our previous study.
PCR amplicons derived from *F. nucleatum* isolates presenting in saliva and those derived from non-isolated strain are shown as blue and pink arrowhead, respectively. It is unclear which amplicon is derived from strain P7, the first or second one from the top in Type I-B2 in the patient E.
M: Maker of gene size

Method: AP-PCR; Sample: Isolates

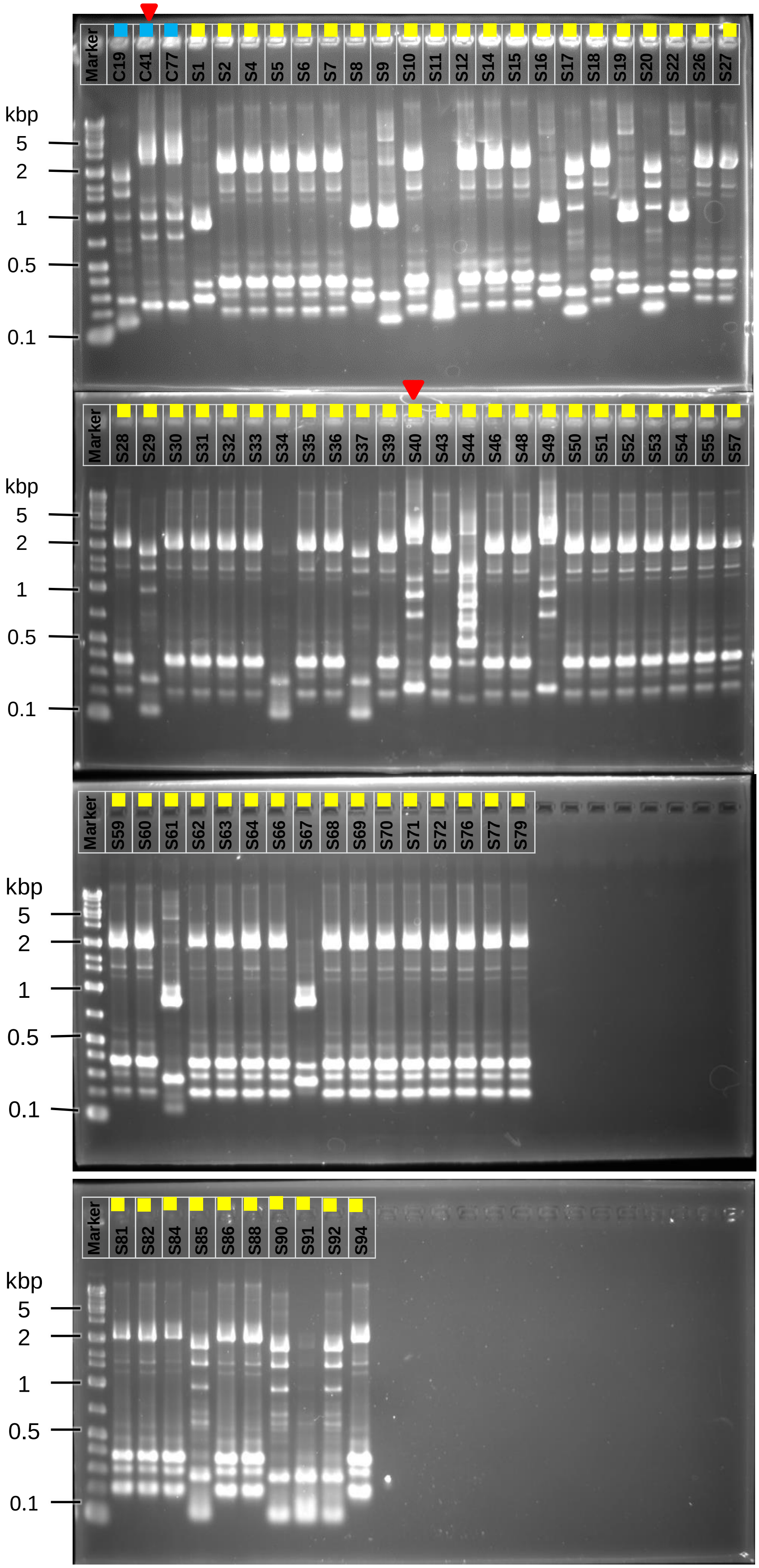

Supplementary Figure S6. The AP-PCR patterns of the *F. nucleatum* strains isolated from both CRC specimen and saliva of the CRC patient O
All the isolates (96 isolates/sample) obtained from both the biopsy samples were analyzed using *F. nucleatum*-specific primers and identified as *F. nucleatum*. The isolates identified as *F. nucleatum* derived from the CRC specimen and saliva samples were labeled with blue and yellow squares, respectively. Isolate C41 and S40 (red triangle) were used as representative isolates of CRC biopsy and saliva samples, respectively, in the application test (Figure 5a). AP-PCR was performed using the primer D11344.

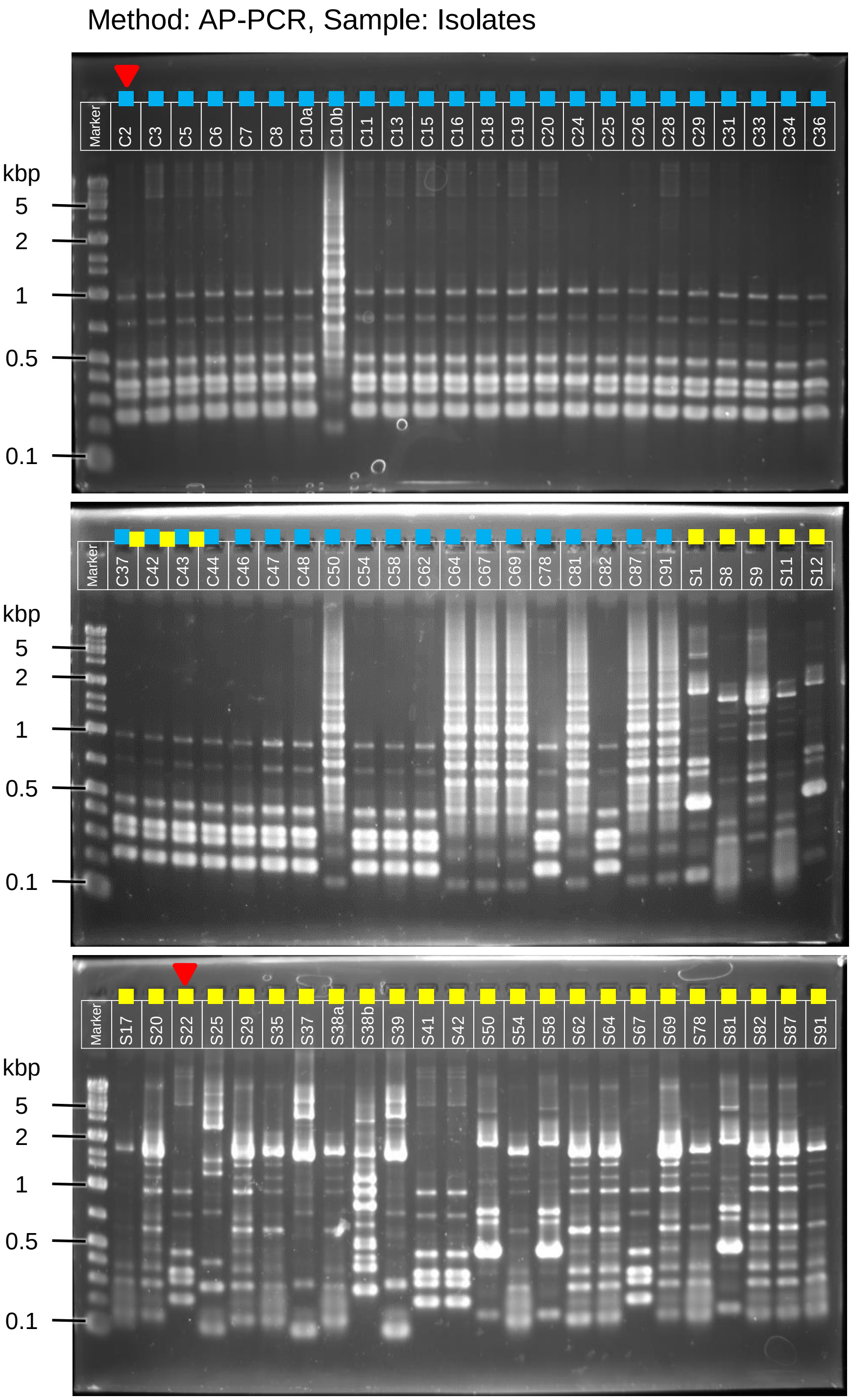

Supplementary Figure S7. The AP-PCR patterns of the *F. nucleatum* strains isolated from both CRC specimen and saliva samples of the CRC patient P

All the isolates (96 isolates/sample) obtained from both the biopsy samples were analyzed using *F. nucleatum*-specific primers and identified as *F. nucleatum*. The isolates identified as *F. nucleatum* derived from the CRC specimen and saliva samples were labeled with blue and yellow squares, respectively. Isolate C2 and S22 (red triangle) were used as representative isolates of CRC biopsy and saliva samples, respectively, in the application test (Figure 5a). AP-PCR was performed using the primer D11344.

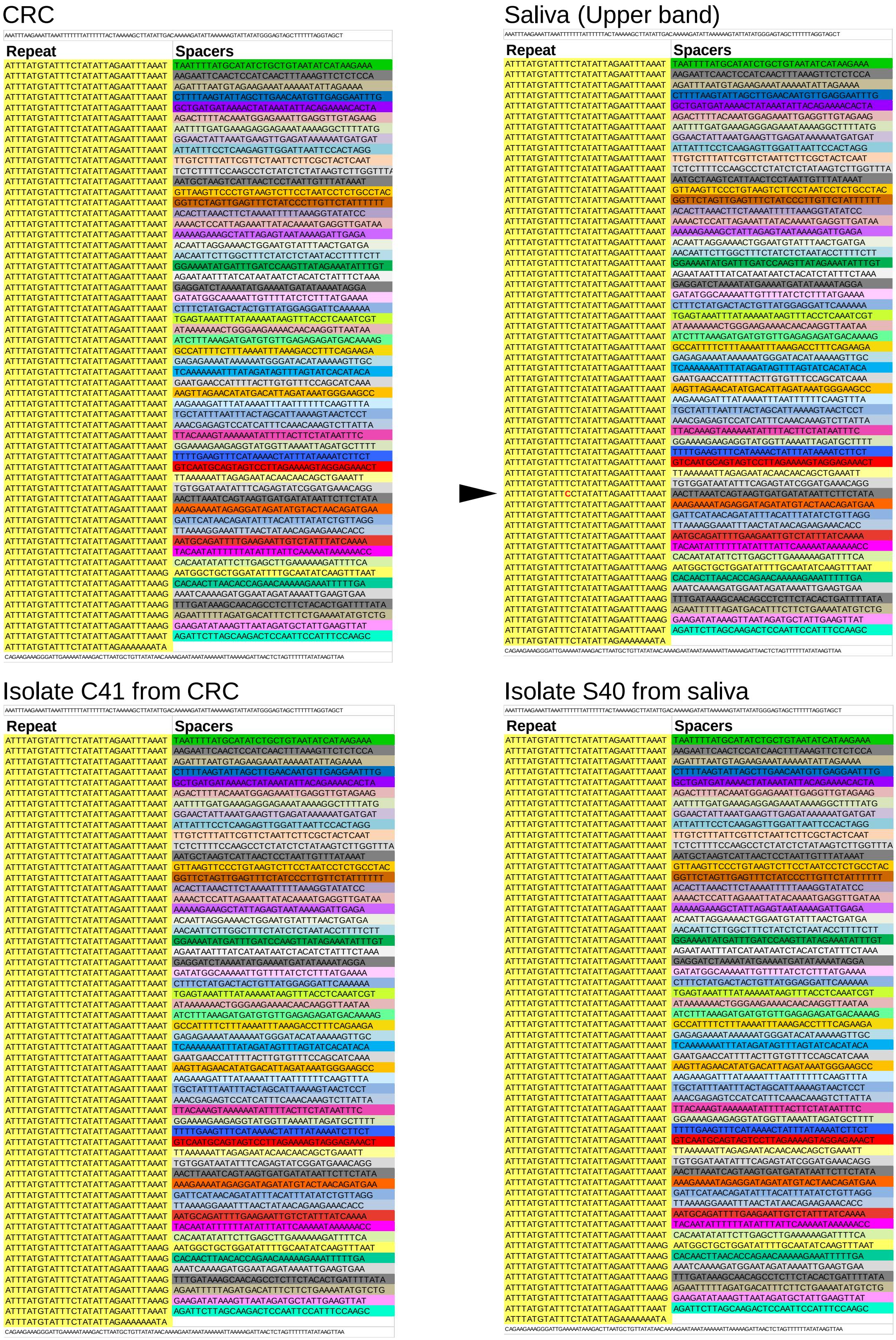

Supplementary Figure S8. DNA sequences of same-sized *F. nucleatum*-strain typing PCR amplicons derived from CRC specimen, saliva, and isolates (Subject O in Fig. 5a)

Base sequences highlighted in yellow and other colors are repeat sequences and spacer sequences, respectively. Identical sequences were detected from the CRC specimen and isolates. There was only a one-base difference as shown in red in the repeat sequences (arrowheaded line) between sequences derived from saliva and others. These sequences were identified as *Fusobacterium nucleatum* subsp. *polymorphum* by BLAST.

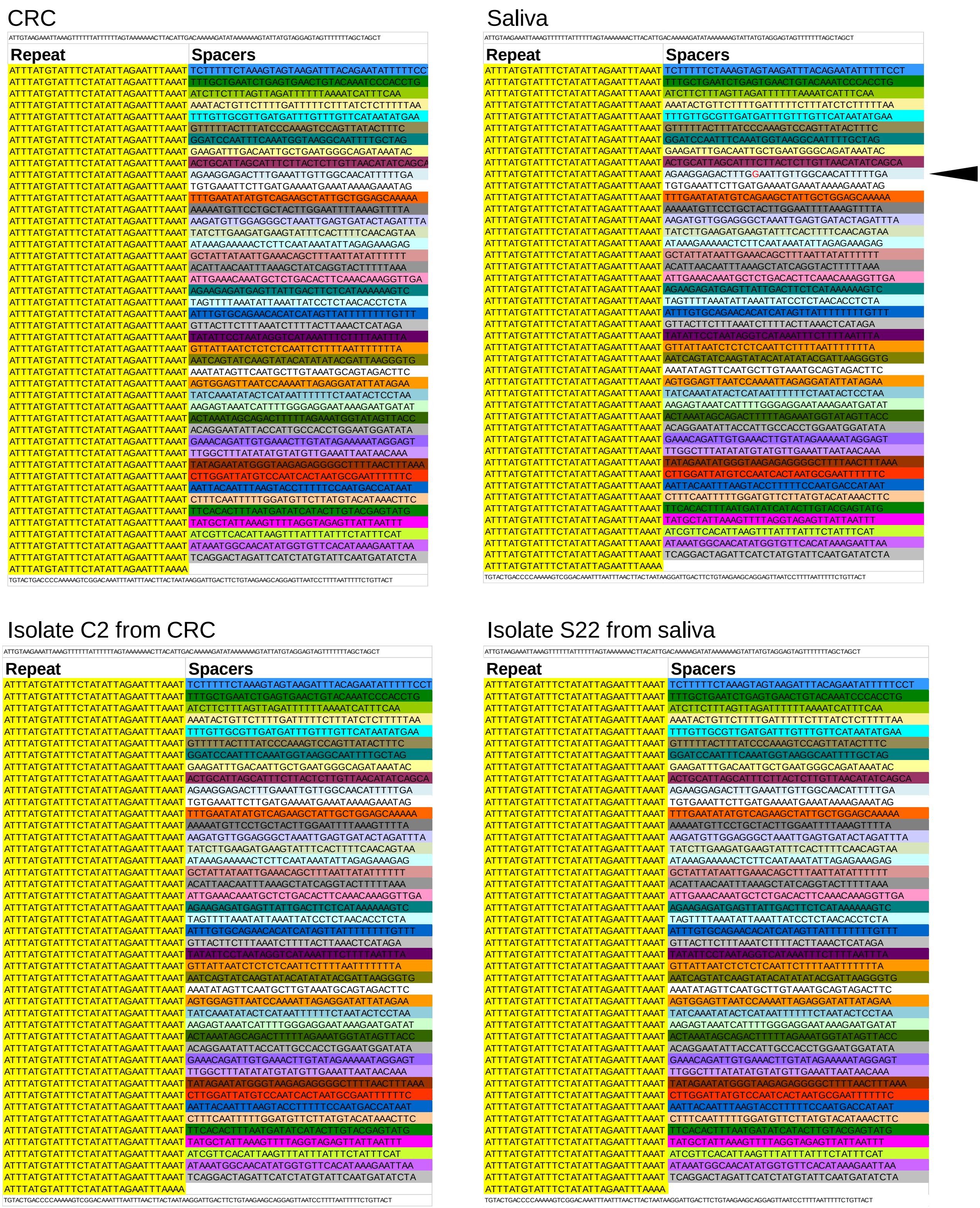

Supplementary Figure S9. DNA sequences of same-sized *F. nucleatum*-strain typing PCR amplicons derived from CRC specimen, saliva, and isolates (Subject P in Figure 5a)

Base sequences highlighted in yellow and other colors are repeat sequences and spacer sequences, respectively. Identical sequences were detected from the CRC specimen and isolates. There was only a one-base difference as shown in red in the spacer sequences (arrowheaded line) between sequences derived from saliva and others. These sequences were identified as *Fusobacterium nucleatum* subsp. *polymorphum* by BLAST.

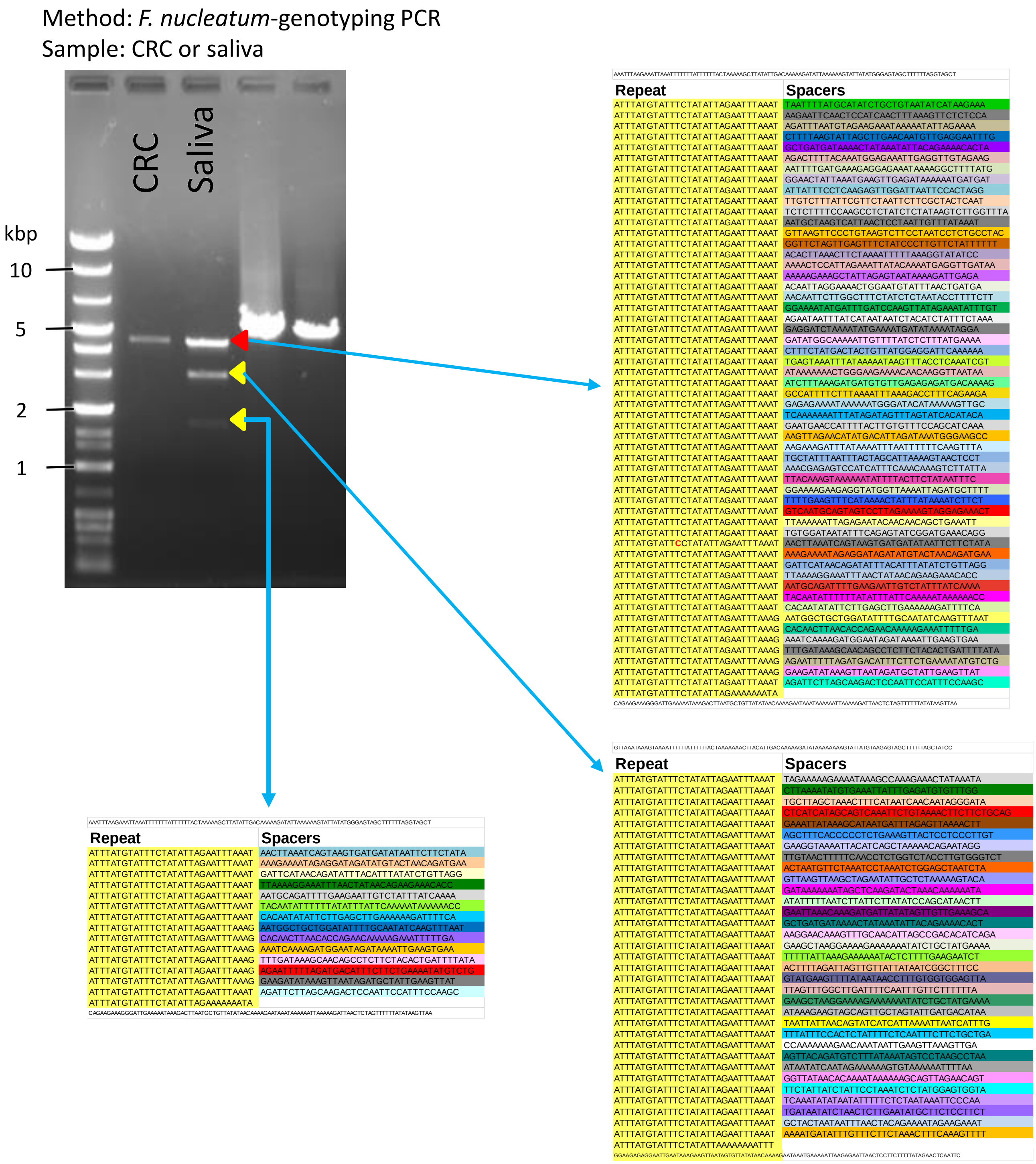

Supplementary Figure S10. DNA sequences of other *F. nucleatum*-strain typing PCR amplicons derived from saliva (Subject O)

Base sequences highlighted in yellow and other colors are repeat sequences and spacer sequences, respectively. All bands are derived from the CRISPR–Cas system. All detected amplicons are CRISPR-associated regions. Each amplicon is derived from each strain of *Fusobacterium* spp.

Method: AP-PCR; Sample: Isolates

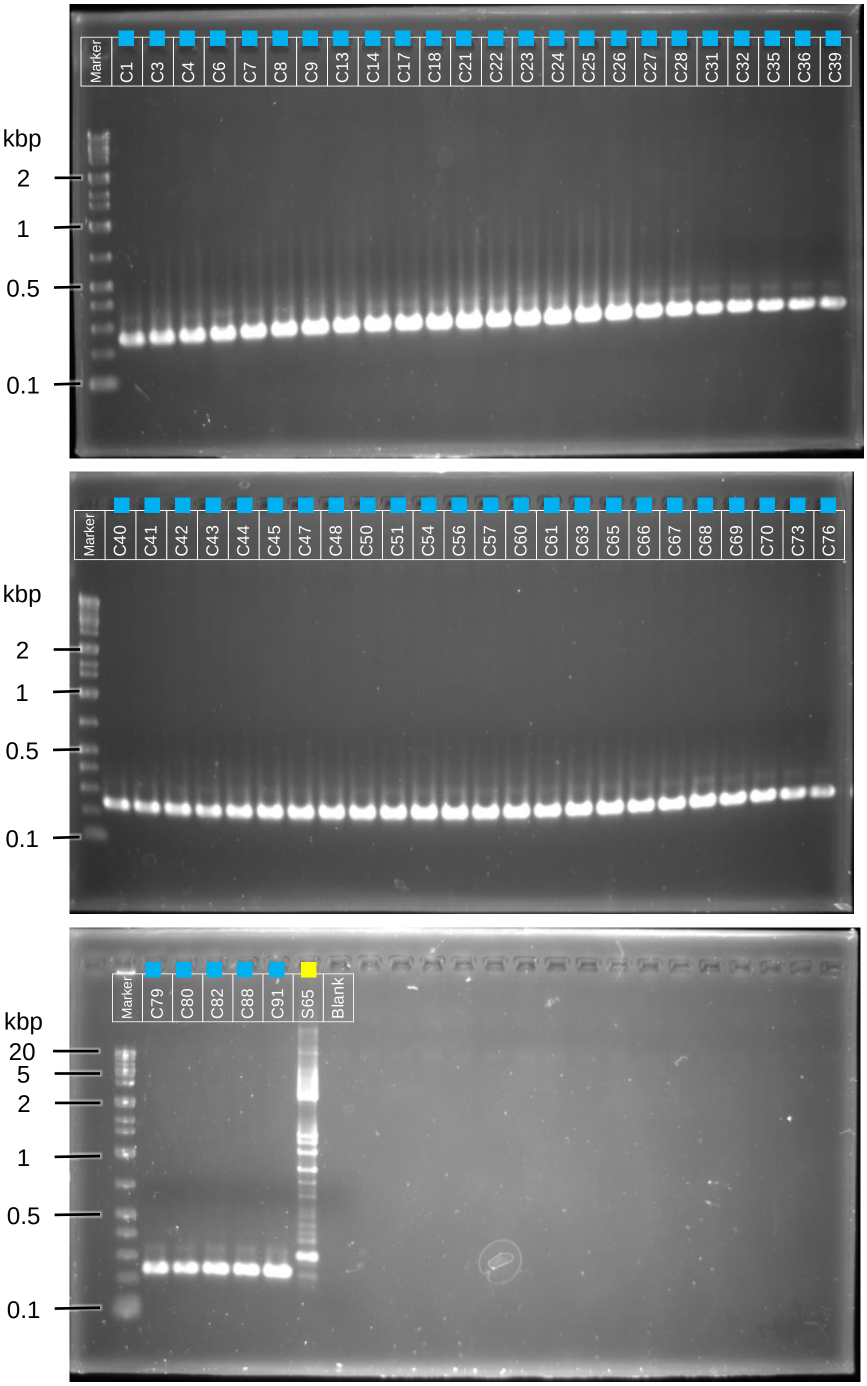

Supplementary Figure S11. The AP-PCR patterns of the *F. nucleatum* strains isolated from both CRC specimen and saliva samples of the CRC patient Q

All the isolates (96 isolates/sample) obtained from both the specimens were analyzed using *F. nucleatum*-specific primers and identified as *F. nucleatum*. The isolates identified as *F. nucleatum* derived from the CRC specimen and saliva samples were labeled with blue and yellow squares, respectively. There was no identical strain from both specimens.

AP-PCR was performed using the primer D11344.

Method: *F. nucleatum*-genotyping PCR; Sample: CRC or saliva

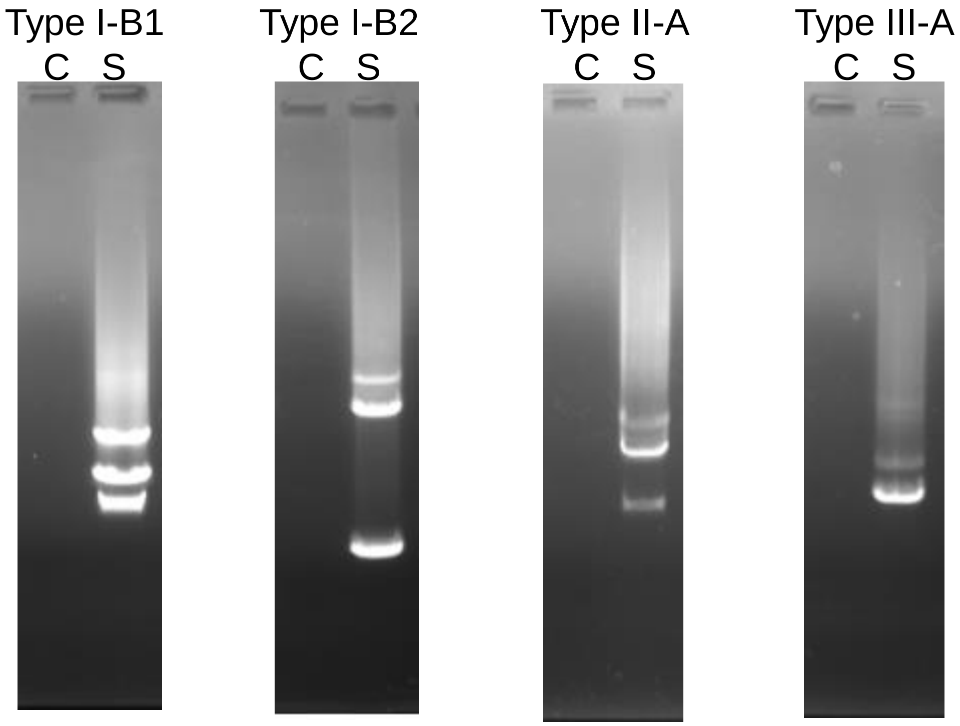

Supplementary Figure S12. The *F. nucleatum*-strain typing PCR analysis did not detect identical strain from both specimens derived from the Patient Q (There was no PCR product from the CRC specimen).
C: CRC specimen; S: Saliva

Method: *F. nucleatum*-genotyping PCR; Sample: Isolates

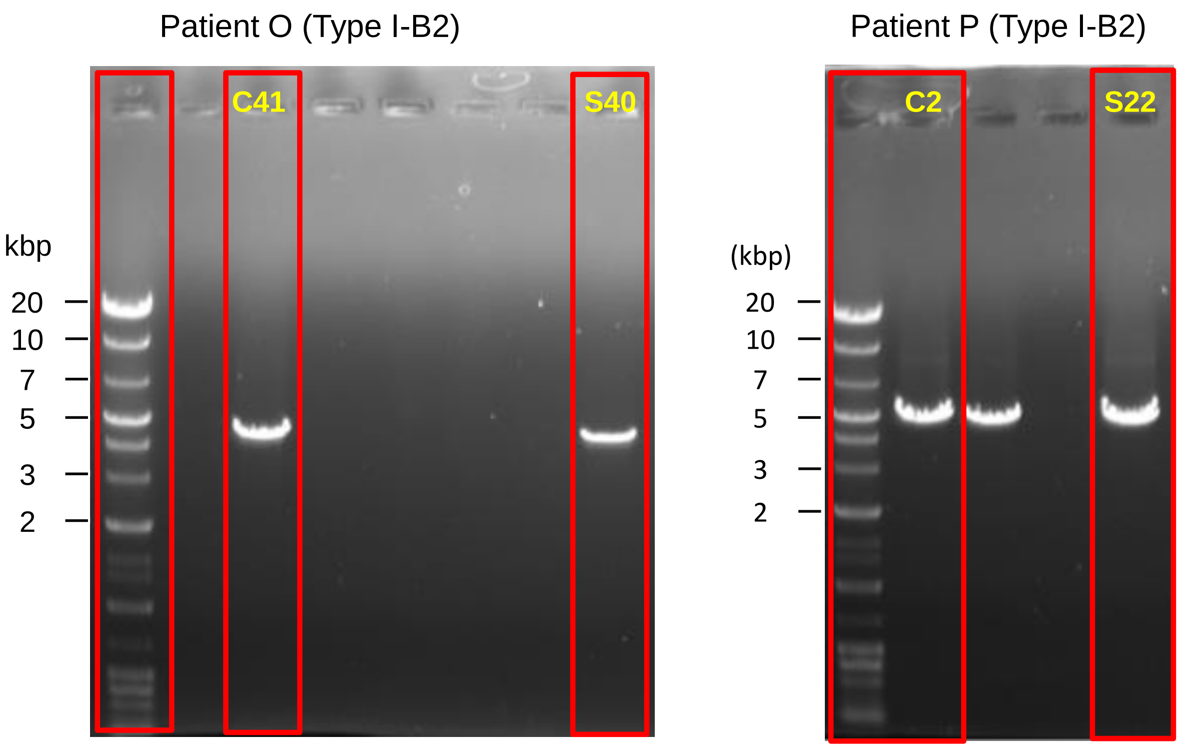

Supplementary Figure S13. The original gel images of Figure 5a.
Markers and PCR products (in red boxes) in the original gels have been cut and pasted to Fig. 5a for clarity. In other lanes, other strains or a negative control was applied.

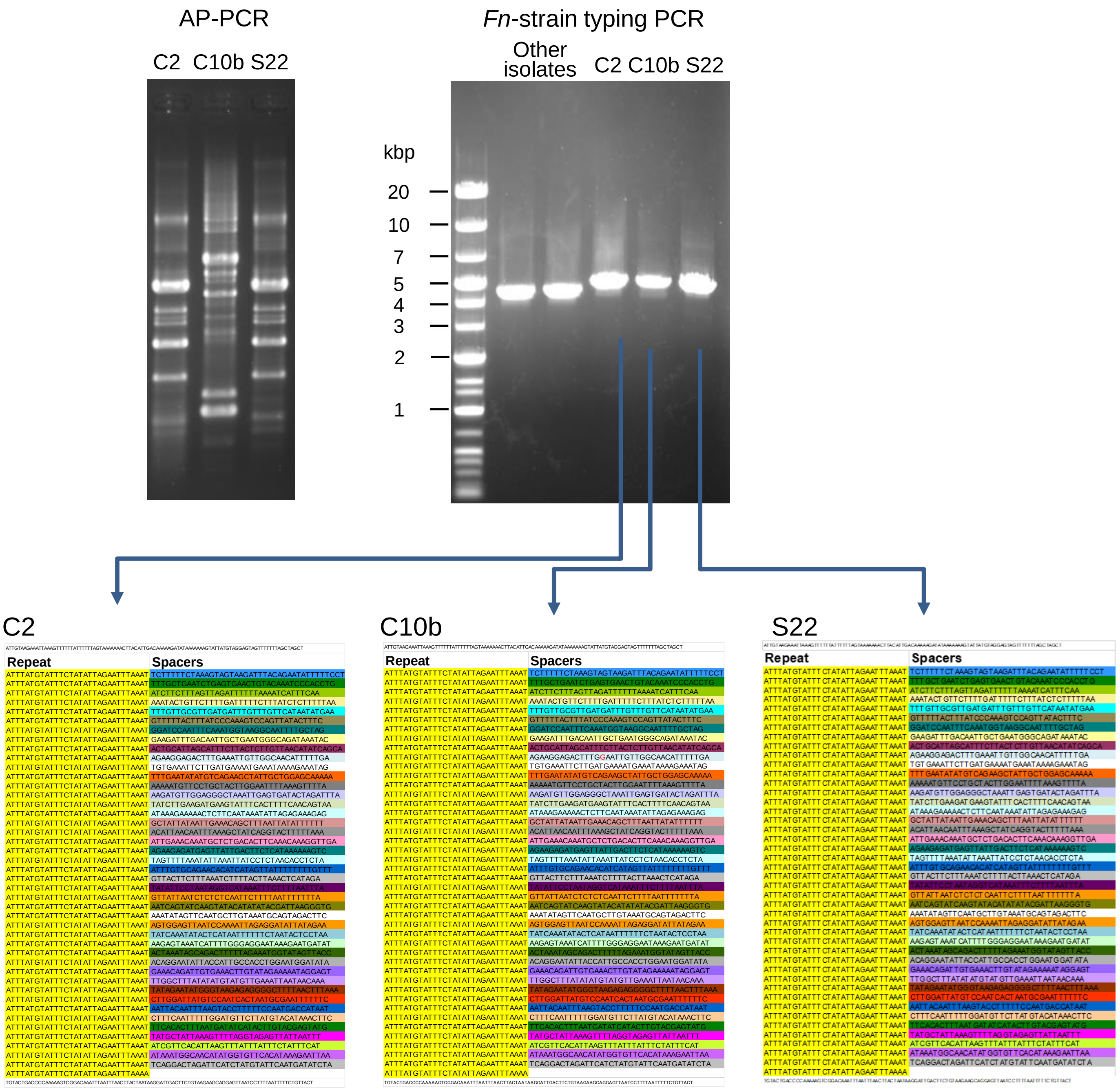

Supplementary Figure S14. Same-sized *F. nucleatum* strain-typing PCR amplicons had identical DNA sequences.

Same-sized amplicons were detected from isolates C10b, C2, and S22 obtained from patient P, although the AP-PCR pattern of C10b differed from that of C2 and S22 (upper part). The DNA sequence of the C10b amplicon was identical to that of the C2 and S22 amplicons (bottom part).

Type I-B1

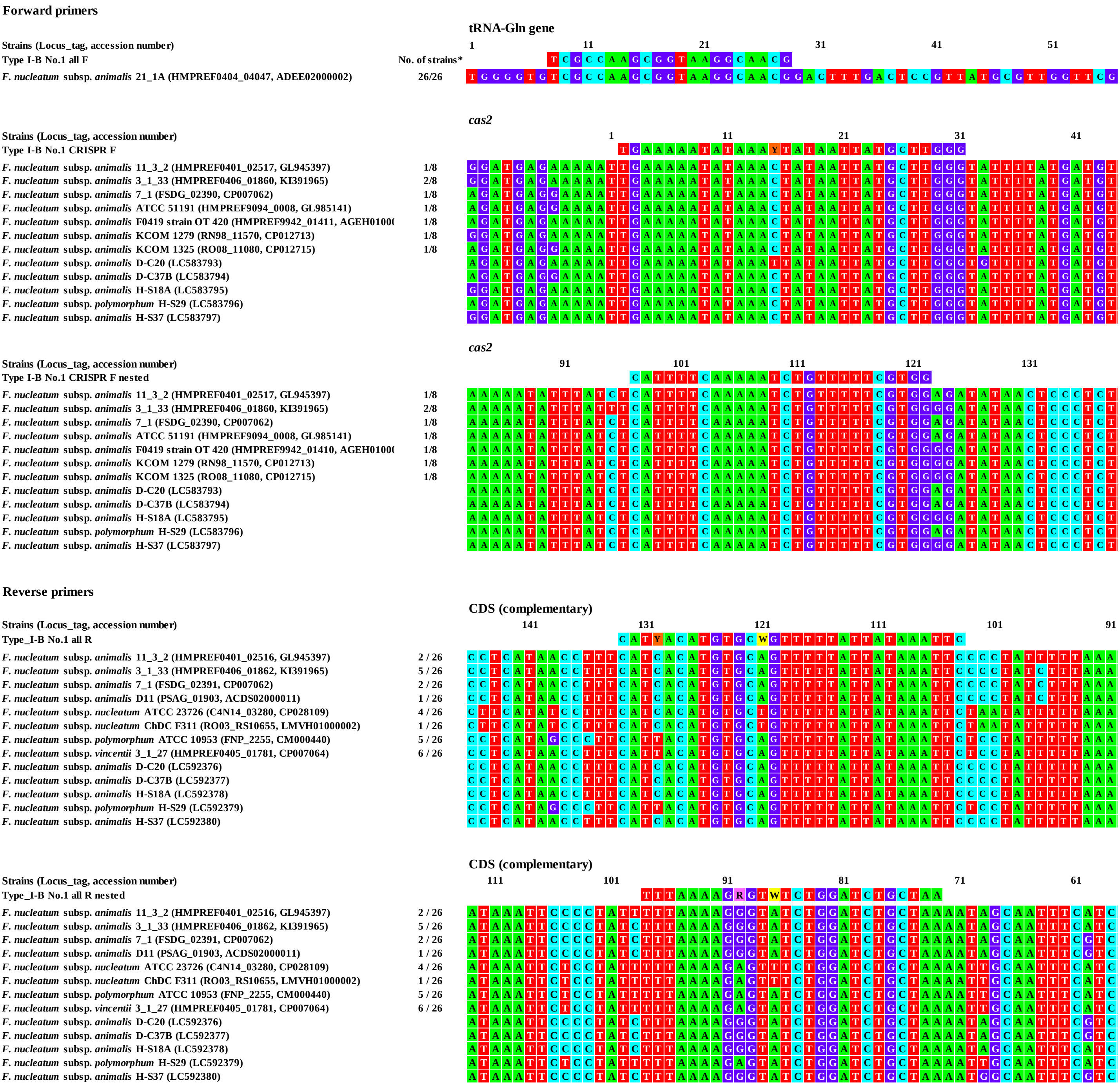

Supplementary Figure S15. DNA sequences for design of primers
*The number of strains that have a cording sequence with the same sequence among all strains obtained from the database

Type I-B2

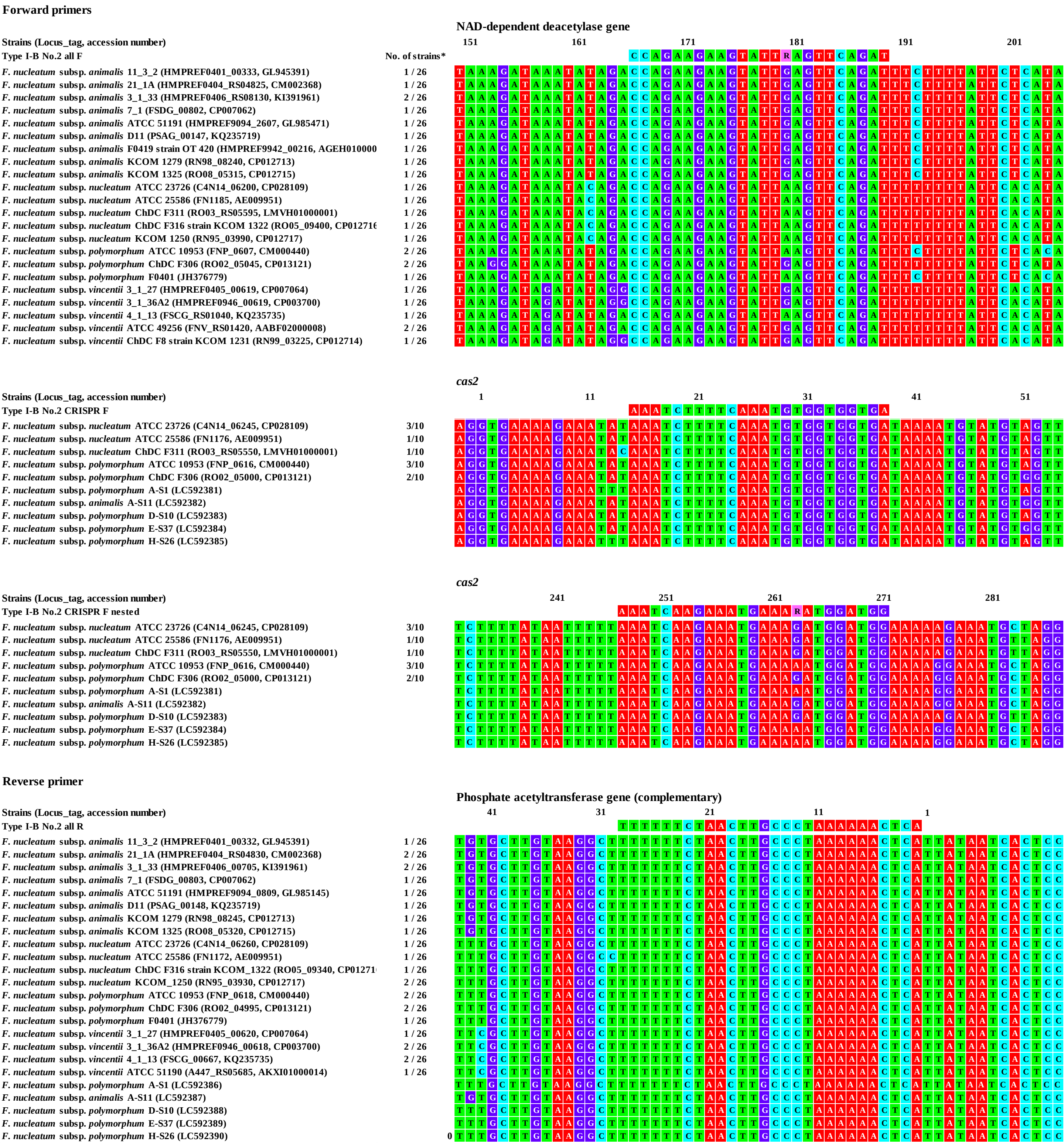

Supplementary Figure S15. DNA sequences for design of primers (continued)
*The number of strains that have a cording sequence with the same sequence among all strains obtained from the database

Type II-A

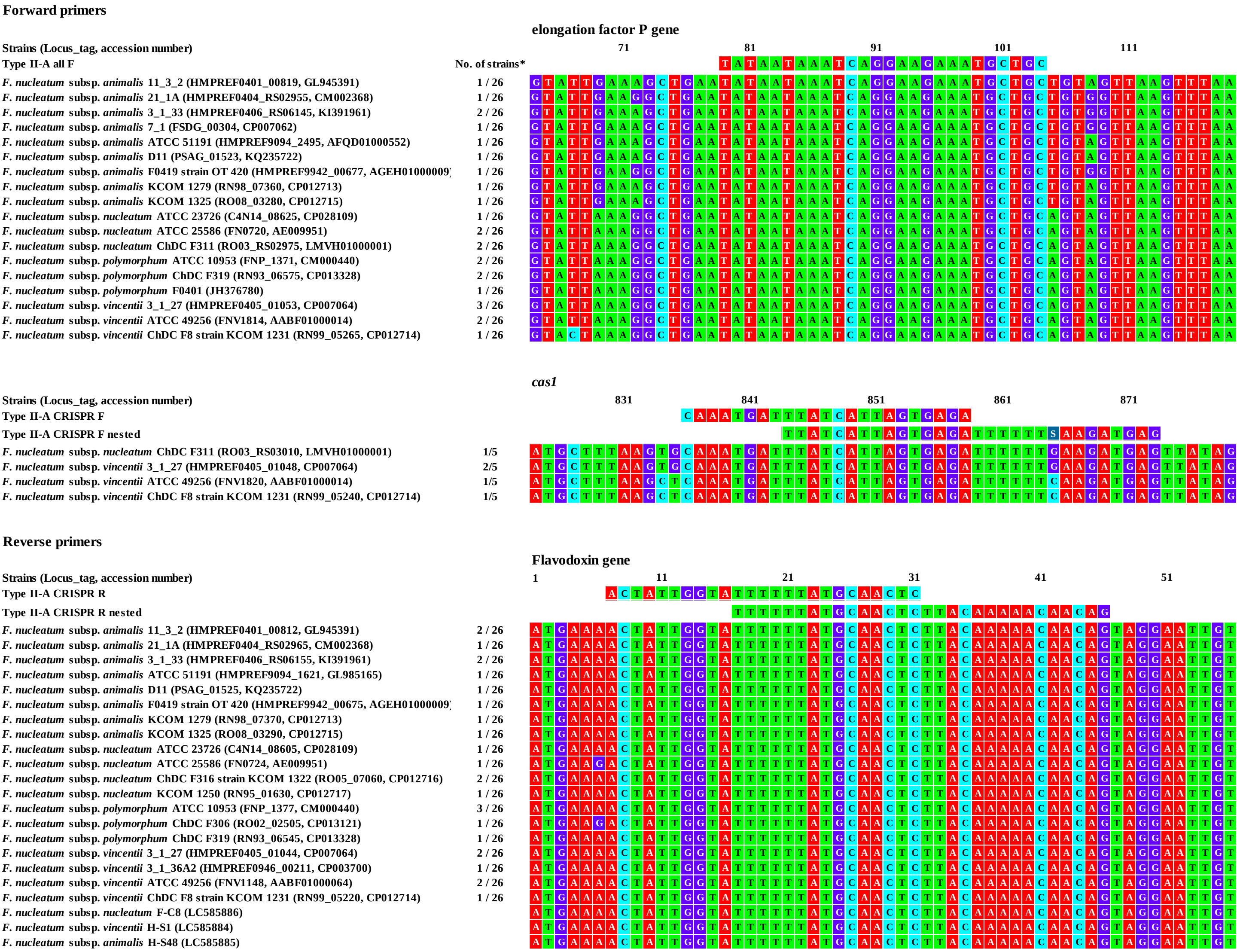

Supplementary Figure S15. DNA sequences for design of primers (continued)
*The number of strains that have a cording sequence with the same sequence among all strains obtained from the database

Type III-A

Forward primers

tRNA (adenosine(37)-N6)-threonylcarbamoyltransferase complex transferase subunit TsaD gene

Strains (Locus_tag, accession number)	Strain number*	271 281 291 301 311
Type III-A all F		TCATTTGCAAAAGGTTTATCTTATGC
F. nucleatum subsp. *animalis* 11_3_2 (HMPREF0401_00119, GL945391)	1 / 26	TTACTTGTGGGAATTTCATTTGCAAAAGGTTTATCTTATGCAAAAAATATTCCTAT
F. nucleatum subsp. *animalis* 21_1A (HMPREF0404_RS05955, CM002368)	1 / 26	TTACTTGTGGGGATTTCATTTGCAAAAGGTTTATCTTATGCAAAAAATATTCCTAT
F. nucleatum subsp. *animalis* 3_1_33 (HMPREF0406_RS08975, KI391961)	3 / 26	TTACTTGTGGGGATTTCATTTGCAAAAGGTTTATCTTATGCAAAAAATATTCCTAT
F. nucleatum subsp. *animalis* ATCC 51191 (HMPREF9094_1276, GL985149)	1 / 26	TTACTTGTGGGAATTTCATTTGCAAAAGGTTTATCTTATGCAAAAAATATTCCTAT
F. nucleatum subsp. *animalis* D11 (PSAG_00394, KQ235716)	1 / 26	TTACTTGTGGGAATTTCATTTGCAAAAGGTTTATCTTATGCAAAAAATATTCCTAT
F. nucleatum subsp. *animalis* F0419 strain OT 420 (HMPREF9942_02278, AGEH0100003(	1 / 26	TTACTTGTGGGAATTTCATTTGCAAAAGGTTTATCTTATGCAAAAAATATTCCTAT
F. nucleatum subsp. *animalis* KCOM 1279 (RN98_08855, CP012713)	1 / 26	TTACTTGTGGGAGTTTCATTTGCAAAAGGTTTATCTTATGCAAAAAATATTCCTAT
F. nucleatum subsp. *animalis* KCOM 1325 (RO08_06195, CP012715)	1 / 26	TTACTTGTGGGAATTTCATTTGCAAAAGGTTTATCTTATGCAAAAAATATTCCTAT
F. nucleatum subsp. *nucleatum* ATCC 23726 (C4N14_09495, CP028109)	1 / 26	TTACTTGTGGGAATTTCATTTGCAAAAGGTTTATCTTATGCAAAAAATATTCCTAT
F. nucleatum subsp. *nucleatum* ATCC 25586 (FN0549, AE009951)	1 / 26	TTACTTGTGGGAGTTTCATTTGCAAAAGGTTTATCTTATGCAAAAAATATTCCTAT
F. nucleatum subsp. *nucleatum* ChDC F311 (RO03_RS02120, LMVH01000001)	1 / 26	TTACTTGTGGGAGTTTCATTTGCAAAAGGTTTATCTTATGCAAAAAATATTCCTAT
F. nucleatum subsp. *nucleatum* ChDC F316 strain KCOM 1322 (RO05_06185, CP012716)	1 / 26	TTACTTGTGGGAGTTTCATTTGCAAAAGGTTTATCTTATGCAAAAAATATTCCTAT
F. nucleatum subsp. *nucleatum* KCOM 1250 (RN95_00760, CP012717)	1 / 26	TTACTTGTGGGAATTTCATTTGCAAAAGGTTTATCTTATGCAAAAAATATTCCTAT
F. nucleatum subsp. *polymorphum* ATCC 10953 (FNP_1813, CM000440)	2 / 26	TTACTTGTGGGAGTTTCATTTGCAAAAGGTTTATCTTATGCAAAAAATATTCCTAT
F. nucleatum subsp. *polymorphum* ChDC F306 (RO02_01360, CP013121)	2 / 26	TTACTTGTCGGAATTTCATTTGCAAAAGGTTTATCTTATGCAAAAAATATTTCTAT
F. nucleatum subsp. *polymorphum* F0401 (JH376781)	1 / 26	TTACTTGTGGGAATTTCATTTGCAAAAGGTTTATCTTATGCAAAAAATATTCCTAT
F. nucleatum subsp. *vincentii* 3_1_27 (HMPREF0405_01166, CP007064)	1 / 26	TTACTTGTGGGAATTTCATTTGCAAAAGGTTTATCTTATGCAAAAAATATTCCTAT
F. nucleatum subsp. *vincentii* 3_1_36A2 (HMPREF0946_00095, CP003700)	1 / 26	TTACTTGTGGGAATTTCATTTGCAAAAGGTTTATCTTATGCAAAAAATATTCCTAT
F. nucleatum subsp. *vincentii* 4_1_13 (FSCG_RS03640, KQ235735)	1 / 26	TTACTTGTGGGAATTTCATTTGCAAAAGGTTTATCTTATGCAAAAAATATTCCTAT
F. nucleatum subsp. *vincentii* ATCC 49256 (FNV1367, AABF01000048)	1 / 26	TTACTTGTGGGAATTTCATTTGCAAAAGGTTTATCTTATGCAAAAAATATTCCTAT
F. nucleatum subsp. *vincentii* ATCC 51190 (A447_RS03975, AKXI01000029)	1 / 26	TTACTTGTGGGGATTTCATTTGCAAAAGGTTTATCTTATGCAAAAAATATTCCTAT
F. nucleatum subsp. *vincentii* ChDC F8 strain KCOM 1231 (RN99_05755, CP012714)	1 / 26	TTACTTGTGGGAATTTCATTTGCAAAAGGTTTATCTTATGCAAAAAATATTCCTAG

cas1

Strains (Locus_tag, accession number)		781 791 801 811 821 831
Type III-A CRISPR F		TTTGATGAAAAAAGTGGAGGAG
F. nucleatum subsp. *animalis* ATCC 51191 (HMPREF9094_1263, GL985149)	1/5	TTATGAAGATTTTGATTTTGATGAAAAAAGTGGAGGAGTATTTTTGAATAAAAAAG
F. nucleatum subsp. *polymorphum* ChDC F306 (RO02_01410, CP013121)	2/5	TTATGAAGATTTTGACTTTGATGAAAAAAGTGGAGGAGTGTTTTTAAATAAAAAAG
F. nucleatum subsp. *polymorphum* F0401 (JH376781)	1/5	TTATGAAGATTTTGACTTTGATGAAAAAAGTGGAGGAGTATTTTTAAATAAAAAAG
F. nucleatum subsp. *vincentii* 4_1_13 (FSCG_RS03600, KQ235735)	1/5	TTATGAAGATTTTAATTTTGATGAAAAAAGTGGAGGAGTATTTTTGAATAAAAAAG
F. nucleatum subsp. *polymorphum* A-S1 (LC592392)		TTATGAAGATTTTGACTTTGATGAAAAAAGTGGAGGAGTGTTTTTAAATAAAAAAG
F. nucleatum subsp. *animalis* D-C37B (LC592391)		TTATGAGGATTTTGATTTTGATGAAAAAAGTGGAGGAGTATTTTTGAATAAAAAAG
F. nucleatum subsp. *polymorphum* E-S32 (LC592395)		TTATGAAGATTTTGACTTTGATGAAAAAAGTGGAGGAGTATTTTTAAATAAAAAAG
F. nucleatum subsp. *nucleatum* F-C8 (LC592394)		TTATGAAGATTTTGATTTTGATGAAAAAAGTGGAGGAGTATTTTTGAATAAAAAAG
F. nucleatum subsp. *polymorphum* F-S14 (LC592393)		NNNNNNNNNNNNNGACTTTGATGAAAAAAGTGGAGGAGTGTTTTTAAATAAAAAAG

cas1

Strains (Locus_tag, accession number)		851 861 871 881 891
Type III-A CRISPR F nested		TTTGAAAARCGTCTTAGACAAGARGTTTC
F. nucleatum subsp. *animalis* ATCC 51191 (HMPREF9094_1263, GL985149)	1/5	AGTTTGTAGAGCAATTTGAAAAGCGTCTTAGACAAGAAGTTTCATATATAACAGAA
F. nucleatum subsp. *polymorphum* ChDC F306 (RO02_01410, CP013121)	2/5	AATTTGTAGAGCAGTTTGAAAAGCGTCTTAGACAAGAGGTTTCATATATAACTGAA
F. nucleatum subsp. *polymorphum* F0401 (JH376781)	1/5	AATTTGTAGAGCAGTTTGAAAAGCGTCTTAGACAAGAGGTTTCATATATAACTGAA
F. nucleatum subsp. *vincentii* 4_1_13 (FSCG_RS03600, KQ235735)	1/5	AGTTTGTAGAACAATTTGAAAAGCGTCTTAGACAAGAAGTTTCATATATAACAGAA
F. nucleatum subsp. *polymorphum* A-S1 (LC592392)		AATTTGTAGAGCAGTTTGAAAAGCGTCTTAGACAAGAGGTTTCATATATAACTGAA
F. nucleatum subsp. *animalis* D-C37B (LC592391)		AGTTTGTAGAGCAATTTGAAAAACGTCTTAGACAAGAAGTTTCATATATAACAGAA
F. nucleatum subsp. *polymorphum* E-S32 (LC592395)		AATTTGTAGAGCAGTTTGAAAAGCGTCTTAGACAAGAGGTTTCATATATAACTGAA
F. nucleatum subsp. *nucleatum* F-C8 (LC592394)		AATTTGTAGAGCAATTTGAAAAGCGTCTTAGACAAGAAGTTTCATATATAACTGAA
F. nucleatum subsp. *polymorphum* F-S14 (LC592393)		AATTTGTAGAGCAGTTTGAAAAGCGTCTTAGACAAGAGGTTTCATATATAACTGAA

Reverse primer

Mannose-1-phosphate guanylyltransferase gene (complementary)

Strains (Locus_tag, accession number)		191 181 171 161 151 141
Type III-A all R		GTAAAGGCAAATTTYCCATGTAAAG
F. nucleatum subsp. *animalis* 11_3_2 (HMPREF0401_00120, GL945391)	1 / 26	AGTAGCCATATCTCTAGTAAAGGCAAATTTCCCATGTAAAGTTCCATTTCCTCCTG
F. nucleatum subsp. *animalis* 21_1A (HMPREF0404_RS05950, CM002368)	1 / 26	AGTAGCCATATCTCTAGTAAAGGCAAATTTCCCATGTAAAGTTCCATTTCCTCCTG
F. nucleatum subsp. *animalis* 3_1_33 (HMPREF0406_RS08970, KI391961)	4 / 26	AGTAGCCATATCTCTAGTAAAGGCAAATTTCCCATGTAAAGTTCCATTTCCTCCTG
F. nucleatum subsp. *animalis* ATCC 51191 (HMPREF9094_1261, GL985149)	1 / 26	AGTAGCCATATCTCTAGTAAAGGCAAATTTCCCATGTAAAGTTCCATTTCCTCCTG
F. nucleatum subsp. *animalis* F0419 strain OT 420 (HMPREF9942_02279, AGEH0100003(	1 / 26	AGTAGCCATATCTCTAGTAAAGGCAAATTTCCCATGTAAAGTTCCATTTCCTCCTG
F. nucleatum subsp. *animalis* KCOM 1279 (RN98_08845, CP012713)	1 / 26	AGTAGCCATATCTCTAGTAAAGGCAAATTTCCCATGTAAAGTTCCATTTCCTCCTG
F. nucleatum subsp. *animalis* KCOM 1325 (RO08_06190, CP012715)	1 / 26	AGTAGCCATATCTCTTGTAAAGGCAAATTTTCCATGTAAAGTTCCATTTCCTCCTG
F. nucleatum subsp. *nucleatum* ATCC 23726 (C4N14_09490, CP028109)	1 / 26	AGTAGCCATATCTCTAGTAAAGGCAAATTTTCCATGTAAAGTTCCATTTCCTCCTG
F. nucleatum subsp. *nucleatum* ATCC 25586 (FN0550, AE009951)	3 / 26	AGTAGCCATATCTCTAGTAAAGGCAAATTTTCCATGTAAAGTTCCATTTCCTCCTG
F. nucleatum subsp. *nucleatum* ChDC F316 strain KCOM 1322 (RO05_06190, CP012716)	1 / 26	AGTAGCCATATCTCTAGTAAAGGCAAATTTTCCATGTAAAGTCCCATTTCCTCCTG
F. nucleatum subsp. *polymorphum* ATCC 10953 (FNP_1814, CM000440)	2 / 26	AGTAGCCATATCTCTAGTAAAGGCAAATTTCCCATGTAAAGTTCCGTTTCCTCCTG
F. nucleatum subsp. *polymorphum* ChDC F319 (RN93_07485, CP013328)	2 / 26	AGTAGCCATATCTCTAGTAAAGGCAAATTTCCCATGTAAAGTTCCGTTTCCTCCTG
F. nucleatum subsp. *polymorphum* F0401 (JH376781)	1 / 26	AGTAGCCATATCTCTAGTAAAGGCAAATTTCCCATGTAAAGTTCCGTTTCCTCCTG
F. nucleatum subsp. *vincentii* 3_1_27 (HMPREF0405_01165, CP007064)	1 / 26	AGTAGCCATATCTCTTGTAAAGGCAAATTTTCCATGTAAAGTTCCATTTCCTCCTG
F. nucleatum subsp. *vincentii* 3_1_36A2 (HMPREF0946_00096, CP003700)	2 / 26	AGTAGCCATATCTCTTGTAAAGGCAAATTTTCCATGTAAAGTTCCATTTCCTCCTG
F. nucleatum subsp. *vincentii* 4_1_13 (FSCG_RS03590, KQ235735)	1 / 26	AGTAGCCATATCTCTTGTAAAGGCAAATTTTCCATGTAAAGTTCCATTTCCTCCTG
F. nucleatum subsp. *vincentii* ATCC 49256 (FNV1365, AABF01000048)	1 / 26	AGTAGCCATATCTCTTGTAAAGGCAAATTTTCCATGTAAAGTTCCATTTCCTCCTG
F. nucleatum subsp. *vincentii* ATCC 51190 (A447_RS03985, AKXI01000029)	1 / 26	AGTAGCCATATCTCTTGTAAAGGCAAATTTTCCATGTAAAGTTCCATTTCCTCCTG
F. nucleatum subsp. *polymorphum* A-S1 (LC592396)		AGTAGCCATATCTCTTGTAAAAGCAAATTTCCCATGTAAAGTTCCATTTCCTCCTG
F. nucleatum subsp. *animalis* D-C37B (LC592397)		AGTAGCCATATCTCTAGTAAAGGCAAATTTCCCATGTAAAGTTCCATTTCCTCCTG
F. nucleatum subsp. *polymorphum* E-S32 (LC592398)		AGTAGCCATATCTCTAGTAAAGGCAAATTTCCCATGTAAAGTTCCGTTTCCTCCTG
F. nucleatum subsp. *nucleatum* F-C8 (LC592399)		AGTAGCCATATCTCTTGTAAAGGCAAATTTTCCATGTAAAGTTCCATTTCCTCCTG
F. nucleatum subsp. *polymorphum* F-S14 (LC592400)		AGTAGCCATATCTCTAGTAAAGGCAAATTTTCCATGTAAAGTTCCGTTTCCTCCTG

Supplementary Figure S15. DNA sequences for design of primers (continued)

*The number of strains that have a cording sequence with the same sequence among all strains obtained from the database

Supplementary Table S1. The number of single nucleotide variants between *F. nucleatum* isolates derived from CRC patients

C-A3 (from CRC) vs. S-A3 (from saliva)

chromosome	position	SNV type	C-A3	S-A3	#SNVs
C-A3	5021	ins	G	GT	1
C-A3	261318	del	TAAGACAAGAC	T	10
C-A3	448270	snp	T	C	1
C-A3	499780	ins	T	TA	1
C-A3	603779	ins	T	TA	1
C-A3	604672	ins	A	AT	1
C-A3	604924	ins	G	GT	1
C-A3	604991	ins	C	CA	1
C-A3	605224	ins	G	GA	1
C-A3	605804	ins	C	CA	1
C-A3	605860	ins	G	GA	1
C-A3	605941	ins	G	GA	1
C-A3	606105	ins	A	AT	1
C-A3	606165	ins	C	CA	1
C-A3	606210	ins	T	TA	1
C-A3	606519	ins	G	GT	1
C-A3	606796	ins	T	TC	1
C-A3	606959	ins	G	GT	1
C-A3	608316	ins	G	GT	1
C-A3	608794	ins	A	AT	1
C-A3	623946	ins	C	CT	1
C-A3	624132	ins	A	AT	1
C-A3	686165	ins	A	AT	1
C-A3	713286	ins	T	TA	1
C-A3	714826	ins	G	GA	1
C-A3	714847	ins	T	TGA	2
C-A3	714916	ins	T	TA	1
C-A3	714925	ins	A	AG	1
C-A3	714965	ins	A	AT	1
C-A3	850009	ins	G	GA	1
C-A3	850331	ins	T	TA	1
C-A3	850471	ins	T	TA	1
C-A3	850887	complex	CAT	GCCATAC	10
C-A3	850945	ins	G	GA	1
C-A3	851075	ins	C	CA	1
C-A3	851591	ins	A	AT	1
C-A3	853341	ins	G	GT	1
C-A3	999995	ins	T	TATCATAAAGATAAAGGAAATTTGG	24
C-A3	1015715	ins	T	TA	1
C-A3	1137992	ins	G	GT	1
C-A3	1508309	snp	C	A	1
C-A3	1514919	ins	C	CT	1
C-A3	1582713	ins	A	AT	1
C-A3	1583709	ins	A	AT	1
C-A3	1584316	ins	A	AT	1
C-A3	1819043	del	AC	A	1
				Total SNVs	88
				genome size	2,276,335
				SNVs / genome size ratio (%)	0.00386586

C-P4 (from CRC) vs. S-P4 (from saliva)

chromosome	position	SNV type	C-P4	S-P4	#SNVs
C-P4	417703	snp	A	G	1
C-P4	683361	ins	G	GA	1
C-P4	869811	ins	T	TA	1
C-P4	878541	ins	A	AT	1
C-P4	2184108	ins	G	GTTTCT	5
				Total SNVs	9
				genome size	2,465,474
				SNVs / genome size ratio (%)	0.00036504

C-V3 (from CRC) vs. S-V3 (from saliva)

chromosome	position	SNV type	C-V3	S-V3	#SNVs
sequence1	272279	ins	T	TAAGCAAGC	8
sequence1	841818	snp	T	C	1
sequence1	1779959	snp	A	G	1
				Total SNVs	10
				genome size	2,139,550
				SNVs / genome size ratio (%)	0.00046739

Supplementary Table S1. The number of single nucleotide variants between *F. nucleatum* isolates derived from CRC patients (continued)

C-P10 (from CRC) vs. S-P10 (from saliva)

chromosome	position	SNV type	C-P10	S-P10	#SNVs
C-P10	28788	snp	A	G	1
C-P10	285962	ins	T	TA	1
C-P10	315740	snp	A	G	1
C-P10	337543	snp	C	T	1
C-P10	338344	ins	C	CA	1
C-P10	347997	del	CA	C	1
C-P10	534061	ins	A	AT	1
C-P10	649436	ins	C	CA	1
C-P10	752669	ins	G	GT	1
C-P10	823139	ins	T	TA	1
C-P10	901153	snp	G	A	1
C-P10	941615	del	TA	T	1
C-P10	953191	ins	A	AT	1
C-P10	957969	ins	C	CT	1
C-P10	1065109	snp	A	C	1
C-P10	1067898	ins	A	AT	1
C-P10	1132796	ins	A	AT	1
C-P10	1332094	ins	A	AT	1
C-P10	1335133	ins	C	CT	1
C-P10	1347628	ins	A	AT	1
C-P10	1419860	ins	C	CT	1
C-P10	1468478	ins	C	CA	1
C-P10	1482647	ins	C	CA	1
C-P10	1504547	ins	A	AT	1
C-P10	1511683	ins	A	ACT	2
C-P10	1615383	ins	C	CT	1
C-P10	1615740	snp	A	T	1
C-P10	1687604	ins	A	AT	1
C-P10	1750610	snp	A	G	1
C-P10	1899894	ins	T	TATGCTC	6
C-P10	1988362	ins	T	TA	1
C-P10	1988554	complex	TGAACCAGCACCAA	AGAAG	19
C-P10	2062172	ins	G	GT	1
C-P10	2132300	ins	T	TA	1
C-P10	2137120	snp	G	A	1
C-P10	2159214	del	CA	C	1
C-P10	2170457	ins	T	TA	1
C-P10	2172962	snp	G	A	1
C-P10	2201795	ins	T	TA	1
C-P10	2236933	ins	A	AT	1
C-P10	2335760	ins	T	TA	1
C-P10	2411581	ins	C	CT	1
C-P10	2414564	del	GT	G	1
C-P10	2517688	snp	C	T	1
C-P10	2524375	ins	T	TG	1
C-P10	2529174	ins	T	TG	1
				Total SNVs	70
				genome size	2,550,062
				SNVs / genome size ratio (%)	0.00274503

C-P11 (from CRC) vs. S-P11 (from saliva)

chromosome	position	SNV type	C-P11	S-P11	#SNVs
C-P11	74001	snp	T	A	1
C-P11	232166	ins	C	CA	1
C-P11	525597	ins	T	TA	1
C-P11	595111	ins	G	GA	1
C-P11	665420	ins	T	TA	1
C-P11	1001202	snp	A	G	1
C-P11	1117830	ins	A	AT	1
C-P11	1119067	ins	C	CT	1
C-P11	1119688	ins	T	TA	1
C-P11	1263161	ins	G	GT	1
C-P11	1268924	ins	G	GT	1
C-P11	1323133	ins	T	TA	1
C-P11	1324680	ins	T	TA	1
C-P11	1404368	ins	G	GT	1
C-P11	1451078	ins	A	AT	1
C-P11	1616328	ins	A	AT	1
C-P11	1621287	ins	C	CT	1
C-P11	1622113	ins	G	GT	1
C-P11	1622863	ins	G	GT	1
C-P11	1907564	ins	G	GT	1
C-P11	1920269	ins	G	GT	1
C-P11	2373007	del	AG	A	1
				Total SNVs	22
				genome size	2,561,355
				SNVs / genome size ratio (%)	0.00085892

Supplementary Table S1. The number of single nucleotide variants between *F. nucleatum* isolates derived from CRC patients (continued)

Between non-paired strains

reference	query	#SNVs	SNVs / genome size ratio (%)
C-A3	C-P4	66556	2.923822724
C-A3	C-V3	62016	2.72437932
C-A3	C-P10	67410	2.96133917
C-A3	C-P11	65936	2.896585959
C-A3	S-P4	66124	2.904844849
C-A3	S-V3	61498	2.701623443
C-A3	S-P10	67401	2.960943798
C-A3	S-P11	65822	2.891577909
C-P4	C-A3	66002	2.677051147
C-P4	C-V3	66922	2.714366487
C-P4	C-P10	31602	1.281781921
C-P4	C-P11	33295	1.350450258
C-P4	S-A3	66087	2.68049876
C-P4	S-V3	66106	2.681269403
C-P4	S-P10	31564	1.280240635
C-P4	S-P11	33251	1.348665612
C-V3	C-A3	61441	2.871678624
C-V3	C-P4	66272	3.097473768
C-V3	C-P10	67597	3.159402678
C-V3	C-P11	67187	3.14023977
C-V3	S-A3	61508	2.874810124
C-V3	S-P4	66067	3.087892314
C-V3	S-P10	67507	3.155196186
C-V3	S-P11	67165	3.139211516
C-P10	C-A3	67276	2.638210365
C-P10	C-P4	31688	1.242636454
C-P10	C-V3	68221	2.675268288
C-P10	C-P11	34331	1.346280992
C-P10	S-A3	67258	2.6375045
C-P10	S-P4	31577	1.238283618
C-P10	S-V3	67540	2.648563055
C-P10	S-P11	34279	1.344241826
C-P11	C-A3	65861	2.571334313
C-P11	C-P4	33248	1.298062939
C-P11	C-V3	67981	2.654103004
C-P11	C-P10	34205	1.335425976
C-P11	S-A3	65855	2.571100062
C-P11	S-P4	33200	1.296188931
C-P11	S-V3	67318	2.628218267
C-P11	S-P10	34219	1.335972561
		Average	2.374168538
		SD	0.716736093

Supplementary Table S2. Information of CRISPR-Cas system of *F. nucleatum* strains in NCBI database

Subspecies	Strains	CRISPR-Cas system （accession number）			
		Type I-B No.1	Type I-B No.2	Type II-A	Type III-A
F. nucleatum subsp. *animalis*	11_3_2	+ (GL945397)	- (GL945391)	- (GL945391)	- (GL945391)
	21_1A	- (ADEE02000002)	- (CM002368)	- (CM002368)	- (CM002368)
	3_1_33	+ (KI391965)	- (KI391961)	- (KI391961)	- (KI391961)
	4_8	+ (CP003723)	- (CP003723)	- (CP003723)	- (CP003723)
	7_1	+ (CP007062)	- (CP007062)	- (CP007062)	- (CP007062)
	ATCC 51191	+ (GL985141)	- (GL985145)	- (GL985165, GL985393)	+ (GL985149)
	D11	- (ACDS02000011)	- (KQ235719)	- (KQ235722)	- (KQ235716)
	F0419 strain OT 420	+ (AGEH01000017, AGEH01000018)	- (AGEH01000006)	- (AGEH01000009)	- (AGEH01000036)
	KCOM 1279	+ (CP012713)	- (CP012713)	- (CP012713)	- (CP012713)
	KCOM 1325	+ (CP012715)	- (CP012715)	- (CP012715)	- (CP012715)
F. nucleatum subsp. *nucleatum*	ATCC 23726	- (CP028109)	+ (CP028109)	- (CP028109)	- (CP028109)
	ATCC 25586	- (AE009951)	+ (AE009951)	- (AE009951)	- (AE009951)
	ChDC F311	- (LMVH01000002)	+ (LMVH01000001)	+ (LMVH01000001)	- (LMVH01000001)
	ChDC F316 strain KCOM 1322	- (CP012716)	+ (CP012716)	- (CP012716)	- (CP012716)
	KCOM 1250	- (CP012717)	+ (CP012717)	- (CP012717)	- (CP012717)
F. nucleatum subsp. *polymorphum*	ATCC 10953	- (CM000440)	+ (CM000440)	- (CM000440)	- (CM000440)
	ChDC F306	- (CP013121)	+ (CP013121)	- (CP013121)	+ (CP013121)
	ChDC F319	- (CP013328)	+ (CP013328)	- (CP013328)	+ (CP013328)
	F0401	- (JH376785)	+ (JH376779)	- (JH376780)	+ (JH376781)
	NCTC10562	- (LN831027)	+ (LN831027)	- (LN831027)	- (LN831027)
F. nucleatum subsp. *vincentii*	3_1_27	- (CP007064)	- (CP007064)	+ (CP007064)	- (CP007064)
	3_1_36A2	- (CP003700)	- (CP003700)	+ (CP003700)	- (CP003700)
	4_1_13	- (KQ235733)	- (KQ235735)	- (KQ235735)	+ (KQ235735)
	ATCC 49256	- (AABF01000001)	- (AABF02000008)	+ (AABF01000064, AABF02000014)	- (AABF01000048)
	ATCC 51190	- (AKXI01000001)	- (AKXI01000014)	- (AKXI01000002)	- (AKXI01000029)
	ChDC F8 strain KCOM 1231	- (CP012714)	- (CP012714)	+ (CP012714)	- (CP012714)

This information was obtained in March of 2015.
+, Strain has this type of CRISPR-Cas system; -, Strain does not have this type of CRISPR-Cas system.

Supplementary Table S3. Primer sequences used for *F. nucleatum*-strain typing PCR

Description	Annealing Temp. (°C)	Forward	Reverse
I-B1_peripheral region	58	TCGCCAAGCGGTAAGGCAACG	CATYACATGTGCWGTTTTTATTATAAATTC
I-B1_1st_PCR	57	TGAAAAATATAAAYTATAATTATGCTTGGG	CATYACATGTGCWGTTTTTATTATAAATTC
I-B1_2nd_PCR	57	CATTTTCAAAAATCTGTTTTTCGTGG	TTTAAAAGRGTWTCTGGATCTGCTAA
I-B2_peripheral region	55	CCAGAAGAAGTATTRAGTTCAGAT	TTTTTTCTAACTTGYCCTAAAAAACTCA
I-B2_1st_PCR	60	AAATCTTTTCAAATGTGGTGGTGA	TTTTTTCTAACTTGYCCTAAAAAACTCA
I-B2_2nd_PCR	60	AAATCAAGAAATGAAARATGGATGG	TTTTTTCTAACTTGYCCTAAAAAACTCA
II-A_peripheral region	58	TATAATAAATCAGGAAGAAATGCTGC	ACTATTGGTATTTTTTATGCAACTC
II-A_1st_PCR	55	CAAATGATTTATCATTAGTGAGA	ACTATTGGTATTTTTTATGCAACTC
II-A_2nd_PCR	60	TTATCATTAGTGAGATTTTTTSAAGATGAG	TTTTTTATGCAACTCTTACAAAAACAACAG
III-A_peripheral region	60	TCATTTGCAAAAGGTTTATCTTATGC	GTAAAGGCAAATTTYCCATGTAAAG
III-A_1st_PCR	60	TTTGATGAAAAAAGTGGAGGAG	GTAAAGGCAAATTTYCCATGTAAAG
III-A_2nd_PCR	60	TTTGAAAARCGTCTTAGACAAGARGTTTC	GTAAAGGCAAATTTYCCATGTAAAG

Supplementary Table S4. The expected PCR amplicon sizes of the CRISPR-associated region obtained from *F. nucleatum* strains

Strain	CRISPR-cas system subtypes	Expected amplicon size (bp)	Accession Number
F. nucleatum subsp. *animalis* JCM11025^{T}	Type I-B1	4834	AFQD01000001
	Type III-A	5886	AFQD01000001
F. nucleatum subsp. *nucleatum* JCM8532^{T}	Type I-B2	2993	AE009951
F. nucleatum subsp. *polymorphum* JCM12990^{T}	Type I-B2	2691	CM000440
F. nucleatum subsp. *vincentii* JCM11023^{T}	Type II-A	Incalculable*	GCA_000182945

The expected amplicon size of *F. nucleatum* subsp. *vincentii* JCM11023^{T} cannot be calculated due to the incompletely assembled genome sequence and the binding positions of the primers, which were located in different contigs (Accession Numbers: AABF01000014 and AABF01000064).

Supplementary Table S5. The validation of PCR amplification by each primer set. The PCR products were sequenced to confirm the successful amplification of the specific target CRISPR-associated regions. Both the 5′- and 3′-ends of the obtained amplicons were Sanger-sequenced and analyzed with the blastn program. All the sequences were 100% matched (E-value 0.0, highest alignment score) to the original genome sequences.

Database	nr/nt		
BLAST algo	megablast		
	1B2N	Type I-B(2)	nucleatum JCM8532^{T} (ATCC 25586)

Query	Sample_Name	CRISPR subtype	Subsp. (Type strain #)	Description	Max Scor	Total Scor	Query Cove	E value	Per. iden	Accession
Query1	1B1-F	Type I-B(1)	animalis (ATCC 51191)	Fusobacterium nucleatum subsp. animalis strain KCOM 1279, complete genome	651	5641	99%	0	86.94	CP012713.1
Query1	1B1-F	Type I-B(1)	animalis (ATCC 51191)	Fusobacterium nucleatum subsp. animalis 7_1, complete genome	627	1784	98%	3.00E-175	86.12	CP007062.1
Query1	1B1-F	Type I-B(1)	animalis (ATCC 51191)	Fusobacterium nucleatum subsp. animalis strain ChDC F332, complete genome	612	4283	99%	8.00E-171	85.64	CP022124.1
Query1	1B1-F	Type I-B(1)	animalis (ATCC 51191)	Fusobacterium nucleatum subsp. animalis strain KCOM 1325, complete genome	588	879	99%	1.00E-163	89.96	CP012715.1
Query1	1B1-F	Type I-B(1)	animalis (ATCC 51191)	Fusobacterium nucleatum subsp. animalis 4_8, complete genome	579	2806	98%	8.00E-161	86.92	CP003723.1
Query1	1B1-F	Type I-B(1)	animalis (ATCC 51191)	Fusobacterium hwasookii ChDC F300, complete genome	69.4	69.4	6%	2.00E-07	100	CP013334.1
Query1	1B1-F	Type I-B(1)	animalis (ATCC 51191)	Fusobacterium pseudoperiodonticum strain KCOM 1263 chromosome, complete	65.8	65.8	5%	3.00E-06	100	CP024700.1
Query1	1B1-F	Type I-B(1)	animalis (ATCC 51191)	Fusobacterium pseudoperiodonticum strain KCOM 1262 chromosome, complete	65.8	65.8	5%	3.00E-06	100	CP024731.1
Query1	1B1-F	Type I-B(1)	animalis (ATCC 51191)	Fusobacterium nucleatum subsp. nucleatum strain KCOM 1250, complete genom	65.8	65.8	5%	3.00E-06	100	CP012717.1
Query	Sample_Name	CRISPR subtype	Subsp. (Type strain #)	Description	Max Score	Total Score	Query Cover	E value	Per. ident	Accession
Query2	1B1-R	Type I-B(1)	animalis (ATCC 51191)	Fusobacterium nucleatum subsp. animalis 7_1, complete genome	575	1261	92%	1.00E-159	86.54	CP007062.1
Query2	1B1-R	Type I-B(1)	animalis (ATCC 51191)	Fusobacterium nucleatum subsp. animalis strain KCOM 1279, complete genome	556	3535	92%	4.00E-154	86.16	CP012713.1
Query2	1B1-R	Type I-B(1)	animalis (ATCC 51191)	Fusobacterium nucleatum subsp. animalis strain ChDC F332, complete genome	553	2779	93%	5.00E-153	85.82	CP022124.1
Query2	1B1-R	Type I-B(1)	animalis (ATCC 51191)	Fusobacterium nucleatum subsp. animalis strain KCOM 1325, complete genome	547	786	88%	2.00E-151	88.17	CP012715.1
Query2	1B1-R	Type I-B(1)	animalis (ATCC 51191)	Fusobacterium nucleatum subsp. animalis 4_8, complete genome	540	4952	94%	4.00E-149	95.82	CP003723.1
Query2	1B1-R	Type I-B(1)	animalis (ATCC 51191)	Fusobacterium nucleatum subsp. vincentii 3_1_27 chromosome 1, complete sequ	104	104	20%	6.00E-18	82.79	CP007064.1
Query	Sample_Name	CRISPR subtype	Subsp. (Type strain #)	Description	Max Score	Total Score	Query Cover	E value	Per. ident	Accession
Query3	1B2N-F	Type I-B(2)	nucleatum (ATCC 25586)	Fusobacterium nucleatum subsp. nucleatum strain 25586 chromosome, complete	1146	3514	100%	0	100	CP028101.1
Query3	1B2N-F	Type I-B(2)	nucleatum (ATCC 25586)	Fusobacterium nucleatum subsp. nucleatum ATCC 25586, complete genome	1146	3514	100%	0	100	AE009951.2
Query3	1B2N-F	Type I-B(2)	nucleatum (ATCC 25586)	Fusobacterium nucleatum subsp. nucleatum strain KCOM 1250, complete genom	484	1730	93%	2.00E-132	82.45	CP012717.1
Query3	1B2N-F	Type I-B(2)	nucleatum (ATCC 25586)	Fusobacterium nucleatum subsp. nucleatum strain ChDC F317, complete genom	466	2703	100%	7.00E-127	81.03	CP022122.1
Query3	1B2N-F	Type I-B(2)	nucleatum (ATCC 25586)	Fusobacterium nucleatum subsp. nucleatum ChDC F316 strain KCOM 1322, com	466	2703	100%	7.00E-127	81.03	CP012716.1
Query3	1B2N-F	Type I-B(2)	nucleatum (ATCC 25586)	Fusobacterium nucleatum subsp. nucleatum ATCC 23726 chromosome, complete	438	3540	100%	2.00E-118	82.98	CP028109.1
Query3	1B2N-F	Type I-B(2)	nucleatum (ATCC 25586)	Fusobacterium nucleatum strain Fn12230 chromosome, complete genome	422	2487	100%	2.00E-113	79.69	CP053468.1
Query3	1B2N-F	Type I-B(2)	nucleatum (ATCC 25586)	Fusobacterium nucleatum subsp. polymorphum genome assembly NCTC10562, c	405	4241	100%	2.00E-108	80.07	LN831027.1
Query3	1B2N-F	Type I-B(2)	nucleatum (ATCC 25586)	Fusobacterium hwasookii strain KCOM 1249 chromosome, complete genome	403	7211	100%	6.00E-108	80.07	CP060112.1
Query3	1B2N-F	Type I-B(2)	nucleatum (ATCC 25586)	Fusobacterium nucleatum subsp. polymorphum strain KCOM 1260 (=ChDC F21	401	2259	92%	2.00E-107	83.6	CP021934.1
Query3	1B2N-F	Type I-B(2)	nucleatum (ATCC 25586)	Fusobacterium nucleatum subsp. polymorphum strain KCOM 1275, complete ge	392	8337	100%	1.00E-104	79.76	CP022123.1
Query3	1B2N-F	Type I-B(2)	nucleatum (ATCC 25586)	Fusobacterium hwasookii ChDC F300, complete genome	390	5306	100%	4.00E-104	83.22	CP013334.1
Query3	1B2N-F	Type I-B(2)	nucleatum (ATCC 25586)	Fusobacterium hwasookii strain KCOM 1253 chromosome, complete genome	390	5479	100%	4.00E-104	79.59	CP060115.1
Query3	1B2N-F	Type I-B(2)	nucleatum (ATCC 25586)	Fusobacterium nucleatum subsp. polymorphum strain ChDC F319 genome	375	5033	93%	1.00E-99	82.74	CP013328.1
Query3	1B2N-F	Type I-B(2)	nucleatum (ATCC 25586)	Fusobacterium nucleatum subsp. polymorphum strain ChDC F306, complete ger	375	4337	100%	1.00E-99	79.32	CP013121.1
Query3	1B2N-F	Type I-B(2)	nucleatum (ATCC 25586)	Fusobacterium hwasookii ChDC F174, complete genome	274	8048	99%	5.00E-69	92.63	CP013331.1
Query3	1B2N-F	Type I-B(2)	nucleatum (ATCC 25586)	Fusobacterium sp. oral taxon 203 genome	75	138	11%	5.00E-09	100	CP016200.1
Query	Sample_Name	CRISPR subtype	Subsp. (Type strain #)	Description	Max Score	Total Score	Query Cover	E value	Per. ident	Accession
Query4	1B2N-R	Type I-B(2)	nucleatum (ATCC 25586)	Fusobacterium nucleatum subsp. nucleatum strain 25586 chromosome, complete	1146	1146	100%	0	100	CP028101.1
Query4	1B2N-R	Type I-B(2)	nucleatum (ATCC 25586)	Fusobacterium nucleatum subsp. nucleatum ATCC 25586, complete genome	1146	1146	100%	0	100	AE009951.2
Query4	1B2N-R	Type I-B(2)	nucleatum (ATCC 25586)	Fusobacterium nucleatum subsp. nucleatum strain ChDC F317, complete genom	1044	1044	100%	0	97.42	CP022122.1
Query4	1B2N-R	Type I-B(2)	nucleatum (ATCC 25586)	Fusobacterium nucleatum subsp. nucleatum ChDC F316 strain KCOM 1322, com	1044	1044	100%	0	97.42	CP012716.1
Query4	1B2N-R	Type I-B(2)	nucleatum (ATCC 25586)	Fusobacterium nucleatum subsp. nucleatum ATCC 23726 chromosome, complete	1038	1038	100%	0	97.26	CP028109.1
Query4	1B2N-R	Type I-B(2)	nucleatum (ATCC 25586)	Fusobacterium nucleatum subsp. nucleatum strain KCOM 1250, complete genom	1038	1038	100%	0	97.26	CP012717.1
Query4	1B2N-R	Type I-B(2)	nucleatum (ATCC 25586)	Fusobacterium sp. oral taxon 203 genome	394	394	41%	3.00E-105	94.51	CP016200.1
Query4	1B2N-R	Type I-B(2)	nucleatum (ATCC 25586)	Fusobacterium nucleatum subsp. polymorphum genome assembly NCTC10562, c	291	291	41%	4.00E-74	87.26	LN831027.1
Query4	1B2N-R	Type I-B(2)	nucleatum (ATCC 25586)	Fusobacterium nucleatum subsp. polymorphum strain KCOM 1260 (=ChDC F21	285	285	41%	2.00E-72	86.87	CP021934.1
Query4	1B2N-R	Type I-B(2)	nucleatum (ATCC 25586)	Fusobacterium nucleatum subsp. polymorphum strain ChDC F319 genome	285	285	41%	2.00E-72	86.87	CP013328.1
Query4	1B2N-R	Type I-B(2)	nucleatum (ATCC 25586)	Fusobacterium nucleatum subsp. polymorphum strain ChDC F306, complete ger	285	285	41%	2.00E-72	86.87	CP013121.1
Query4	1B2N-R	Type I-B(2)	nucleatum (ATCC 25586)	Fusobacterium nucleatum strain Fn12230 chromosome, complete genome	279	279	41%	1.00E-70	86.49	CP053468.1
Query4	1B2N-R	Type I-B(2)	nucleatum (ATCC 25586)	Fusobacterium nucleatum subsp. polymorphum strain KCOM 1275, complete ge	268	268	41%	2.00E-67	85.71	CP022123.1
Query4	1B2N-R	Type I-B(2)	nucleatum (ATCC 25586)	Fusobacterium nucleatum subsp. animalis strain KCOM 1279, complete genome	215	215	41%	3.00E-51	82.06	CP012713.1
Query4	1B2N-R	Type I-B(2)	nucleatum (ATCC 25586)	Fusobacterium nucleatum subsp. animalis 4_8, complete genome	209	209	41%	1.00E-49	81.54	CP003723.1
Query4	1B2N-R	Type I-B(2)	nucleatum (ATCC 25586)	Fusobacterium nucleatum subsp. animalis strain KCOM 1325, complete genome	206	206	41%	2.00E-48	81.3	CP012715.1
Query4	1B2N-R	Type I-B(2)	nucleatum (ATCC 25586)	Fusobacterium nucleatum subsp. animalis 7_1, complete genome	206	206	41%	2.00E-48	81.47	CP007062.1
Query4	1B2N-R	Type I-B(2)	nucleatum (ATCC 25586)	Fusobacterium nucleatum subsp. animalis strain ChDC F332, complete genome	200	200	41%	8.00E-47	80.92	CP022124.1
Query	Sample_Name	CRISPR subtype	Subsp. (Type strain #)	Description	Max Score	Total Score	Query Cover	E value	Per. ident	Accession
Query5	1B2P-F	Type I-B(2)	polymorphum (ATCC 10953)	Fusobacterium nucleatum subsp. polymorphum genome assembly NCTC10562, c	1146	5247	100%	0	100	LN831027.1
Query5	1B2P-F	Type I-B(2)	polymorphum (ATCC 10953)	Fusobacterium nucleatum strain Fn12230 chromosome, complete genome	470	2591	100%	5.00E-128	83.98	CP053468.1
Query5	1B2P-F	Type I-B(2)	polymorphum (ATCC 10953)	Fusobacterium nucleatum subsp. polymorphum strain KCOM 1275, complete ge	468	8589	100%	2.00E-127	81.96	CP022123.1
Query5	1B2P-F	Type I-B(2)	polymorphum (ATCC 10953)	Fusobacterium nucleatum subsp. polymorphum strain ChDC F319 genome	460	5771	100%	3.00E-125	81.76	CP013328.1
Query5	1B2P-F	Type I-B(2)	polymorphum (ATCC 10953)	Fusobacterium nucleatum subsp. polymorphum strain ChDC F306, complete ger	460	5513	100%	3.00E-125	81.76	CP013121.1
Query5	1B2P-F	Type I-B(2)	polymorphum (ATCC 10953)	Fusobacterium nucleatum subsp. polymorphum strain KCOM 1260 (=ChDC F21	453	2220	100%	5.00E-123	80.47	CP021934.1
Query5	1B2P-F	Type I-B(2)	polymorphum (ATCC 10953)	Fusobacterium nucleatum subsp. nucleatum strain 25586 chromosome, complete	403	2727	93%	6.00E-108	80.03	CP028101.1
Query5	1B2P-F	Type I-B(2)	polymorphum (ATCC 10953)	Fusobacterium nucleatum subsp. nucleatum ATCC 25586, complete genome	403	2727	93%	6.00E-108	80.03	AE009951.2
Query5	1B2P-F	Type I-B(2)	polymorphum (ATCC 10953)	Fusobacterium nucleatum subsp. nucleatum ATCC 23726 chromosome, complete	396	4149	93%	9.00E-106	79.93	CP028109.1
Query5	1B2P-F	Type I-B(2)	polymorphum (ATCC 10953)	Fusobacterium nucleatum subsp. nucleatum strain KCOM 1250, complete genom	388	1721	97%	2.00E-103	81.38	CP012717.1
Query5	1B2P-F	Type I-B(2)	polymorphum (ATCC 10953)	Fusobacterium nucleatum subsp. nucleatum strain ChDC F317, complete genom	381	2625	92%	3.00E-101	79.53	CP022122.1
Query5	1B2P-F	Type I-B(2)	polymorphum (ATCC 10953)	Fusobacterium nucleatum subsp. nucleatum ChDC F316 strain KCOM 1322, com	381	2625	92%	3.00E-101	79.53	CP012716.1
Query5	1B2P-F	Type I-B(2)	polymorphum (ATCC 10953)	Fusobacterium hwasookii strain KCOM 1249 chromosome, complete genome	363	7471	100%	9.00E-96	78.85	CP060112.1
Query5	1B2P-F	Type I-B(2)	polymorphum (ATCC 10953)	Fusobacterium hwasookii ChDC F300, complete genome	318	5399	100%	2.00E-82	78.89	CP013334.1
Query5	1B2P-F	Type I-B(2)	polymorphum (ATCC 10953)	Fusobacterium hwasookii strain KCOM 1253 chromosome, complete genome	318	5510	100%	2.00E-82	78.89	CP060115.1
Query5	1B2P-F	Type I-B(2)	polymorphum (ATCC 10953)	Fusobacterium hwasookii ChDC F174, complete genome	135	7294	61%	2.00E-27	74.81	CP013331.1
Query5	1B2P-F	Type I-B(2)	polymorphum (ATCC 10953)	Fusobacterium pseudoperiodonticum strain KCOM 1277 chromosome, complete	69.4	69.4	6%	2.00E-07	97.5	CP024701.1
Query5	1B2P-F	Type I-B(2)	polymorphum (ATCC 10953)	Fusobacterium pseudoperiodonticum strain KCOM 1263 chromosome, complete	69.4	204	19%	2.00E-07	97.5	CP024700.1
Query5	1B2P-F	Type I-B(2)	polymorphum (ATCC 10953)	Fusobacterium pseudoperiodonticum strain KCOM 1262 chromosome, complete	69.4	204	19%	2.00E-07	92	CP024731.1

Supplementary Table S5. The validation of PCR amplification by each primer set (Continued)

Query	Sample_Name	CRISPR subtype	Subsp. (Type strain #)	Description	Max Score	Total Score	Query Cover	E value	Per. ident	Accession
Query6	1B2P-R	Type I-B(2)	polymorphum (ATCC 10953)	Fusobacterium nucleatum subsp. polymorphum genome assembly NCTC10562, c	1181	3908	100%	0	100	LN831027.1
Query6	1B2P-R	Type I-B(2)	polymorphum (ATCC 10953)	Fusobacterium nucleatum strain Fn12230 chromosome, complete genome	931	2468	100%	0	96.15	CP053468.1
Query6	1B2P-R	Type I-B(2)	polymorphum (ATCC 10953)	Fusobacterium nucleatum subsp. polymorphum strain ChDC F319 genome	821	4497	100%	0	89.97	CP013328.1
Query6	1B2P-R	Type I-B(2)	polymorphum (ATCC 10953)	Fusobacterium nucleatum subsp. polymorphum strain ChDC F306, complete ger	817	3699	100%	0	92.53	CP013121.1
Query6	1B2P-R	Type I-B(2)	polymorphum (ATCC 10953)	Fusobacterium nucleatum subsp. polymorphum strain KCOM 1260 (=ChDC F218	802	2023	100%	0	89.47	CP021934.1
Query6	1B2P-R	Type I-B(2)	polymorphum (ATCC 10953)	Fusobacterium nucleatum subsp. polymorphum strain KCOM 1275, complete ger	795	6879	100%	0	89.38	CP022123.1
Query6	1B2P-R	Type I-B(2)	polymorphum (ATCC 10953)	Fusobacterium nucleatum subsp. nucleatum strain 25586 chromosome, complete	289	1798	75%	2.00E-73	87.21	CP028101.1
Query6	1B2P-R	Type I-B(2)	polymorphum (ATCC 10953)	Fusobacterium nucleatum subsp. nucleatum ATCC 25586, complete genome	289	1798	75%	2.00E-73	87.21	AE009951.2
Query6	1B2P-R	Type I-B(2)	polymorphum (ATCC 10953)	Fusobacterium nucleatum subsp. nucleatum ATCC 23726 chromosome, complete	283	2748	75%	8.00E-72	86.82	CP028109.1
Query6	1B2P-R	Type I-B(2)	polymorphum (ATCC 10953)	Fusobacterium nucleatum subsp. nucleatum strain ChDC F317, complete genom	283	1977	75%	8.00E-72	86.82	CP022122.1
Query6	1B2P-R	Type I-B(2)	polymorphum (ATCC 10953)	Fusobacterium nucleatum subsp. nucleatum strain KCOM 1250, complete genom	283	1216	74%	8.00E-72	86.82	CP012717.1
Query6	1B2P-R	Type I-B(2)	polymorphum (ATCC 10953)	Fusobacterium nucleatum subsp. nucleatum ChDC F316 strain KCOM 1322, com	283	1977	77%	8.00E-72	86.82	CP012716.1
Query6	1B2P-R	Type I-B(2)	polymorphum (ATCC 10953)	Fusobacterium hwasookii ChDC F300, complete genome	265	3935	75%	3.00E-66	85.38	CP013334.1
Query6	1B2P-R	Type I-B(2)	polymorphum (ATCC 10953)	Fusobacterium hwasookii strain KCOM 1253 chromosome, complete genome	265	4079	77%	3.00E-66	85.6	CP060115.1
Query6	1B2P-R	Type I-B(2)	polymorphum (ATCC 10953)	Fusobacterium hwasookii strain KCOM 1249 chromosome, complete genome	265	5163	76%	3.00E-66	85.49	CP060112.1
Query6	1B2P-R	Type I-B(2)	polymorphum (ATCC 10953)	Fusobacterium hwasookii ChDC F174, complete genome	259	5639	76%	1.00E-64	85.1	CP013331.1
Query6	1B2P-R	Type I-B(2)	polymorphum (ATCC 10953)	Fusobacterium hwasookii ChDC F206, complete genome	259	259	38%	1.00E-64	85.77	CP013336.1
Query6	1B2P-R	Type I-B(2)	polymorphum (ATCC 10953)	Fusobacterium sp. oral taxon 203 genome	257	325	45%	5.00E-64	85.16	CP016200.1
Query6	1B2P-R	Type I-B(2)	polymorphum (ATCC 10953)	Fusobacterium nucleatum subsp. animalis strain KCOM 1279, complete genome	250	250	39%	8.00E-62	84.58	CP012713.1
Query6	1B2P-R	Type I-B(2)	polymorphum (ATCC 10953)	Fusobacterium nucleatum subsp. animalis 4_8, complete genome	69.4	69.4	5%	2.00E-07	100	CP003723.1

Query	Sample_Name	CRISPR subtype	Subsp. (Type strain #)	Description	Max Score	Total Score	Query Cover	E value	Per. ident	Accession
Query7	2A-F	Type II-A	vincentii (ATCC 49256)	Fusobacterium nucleatum subsp. vincentii ChDC F8 strain KCOM 1231, complet	1144	1144	100%	0	100	CP012714.1
Query7	2A-F	Type II-A	vincentii (ATCC 49256)	Fusobacterium nucleatum subsp. vincentii 3_1_27 chromosome 1, complete sequ	1050	1050	100%	0	97.25	CP007064.1
Query7	2A-F	Type II-A	vincentii (ATCC 49256)	Fusobacterium nucleatum isolate MGYG-HGUT-01347 genome assembly, chrom	1050	1050	100%	0	97.25	LR698955.1
Query7	2A-F	Type II-A	vincentii (ATCC 49256)	Fusobacterium pseudoperiodonticum strain KCOM 1283 chromosome, complete	643	643	99%	3.00E-180	85.58	CP024698.1
Query7	2A-F	Type II-A	vincentii (ATCC 49256)	Fusobacterium pseudoperiodonticum strain KCOM 2555 chromosome, complete	632	632	99%	6.00E-177	85.23	CP024704.1
Query7	2A-F	Type II-A	vincentii (ATCC 49256)	Fusobacterium pseudoperiodonticum strain KCOM 1282 chromosome, complete	623	623	100%	4.00E-174	84.94	CP024702.1

Query	Sample_Name	CRISPR subtype	Subsp. (Type strain #)	Description	Max Score	Total Score	Query Cover	E value	Per. ident	Accession
Query8	2A-R	Type II-A	vincentii (ATCC 49256)	Fusobacterium nucleatum subsp. vincentii 3_1_27 chromosome 1, complete sequ	1134	1134	100%	0	99.68	CP007064.1
Query8	2A-R	Type II-A	vincentii (ATCC 49256)	Fusobacterium nucleatum subsp. vincentii strain KCOM 2931 chromosome, comp	1129	1129	100%	0	99.52	CP024749.1
Query8	2A-R	Type II-A	vincentii (ATCC 49256)	Fusobacterium nucleatum subsp. vincentii ChDC F8 strain KCOM 1231, complet	1118	1118	100%	0	99.19	CP012714.1
Query8	2A-R	Type II-A	vincentii (ATCC 49256)	Fusobacterium nucleatum isolate MGYG-HGUT-01347 genome assembly, chrom	1090	1090	100%	0	98.39	LR698955.1
Query8	2A-R	Type II-A	vincentii (ATCC 49256)	Fusobacterium nucleatum subsp. animalis strain KCOM 1325, complete genome	767	767	80%	0	94.41	CP012715.1
Query8	2A-R	Type II-A	vincentii (ATCC 49256)	Fusobacterium nucleatum subsp. animalis 4_8, complete genome	767	870	92%	0	94.41	CP003723.1
Query8	2A-R	Type II-A	vincentii (ATCC 49256)	Fusobacterium nucleatum subsp. animalis strain ChDC F332, complete genome	761	761	80%	0	94.21	CP022124.1
Query8	2A-R	Type II-A	vincentii (ATCC 49256)	Fusobacterium nucleatum subsp. animalis strain KCOM 1279, complete genome	761	761	80%	0	94.21	CP012713.1
Query8	2A-R	Type II-A	vincentii (ATCC 49256)	Fusobacterium nucleatum subsp. animalis 7_1, complete genome	761	761	80%	0	94.21	CP007062.1
Query8	2A-R	Type II-A	vincentii (ATCC 49256)	Fusobacterium sp. oral taxon 203 genome	712	823	92%	0	92.42	CP016200.1
Query8	2A-R	Type II-A	vincentii (ATCC 49256)	Fusobacterium nucleatum subsp. nucleatum ATCC 23726 chromosome, complete	686	686	77%	0	92.68	CP028109.1
Query8	2A-R	Type II-A	vincentii (ATCC 49256)	Fusobacterium nucleatum subsp. nucleatum strain 25586 chromosome, complete	680	680	77%	0	92.48	CP028101.1
Query8	2A-R	Type II-A	vincentii (ATCC 49256)	Fusobacterium nucleatum subsp. nucleatum ATCC 25586, complete genome	680	680	77%	0	92.48	AE009951.2
Query8	2A-R	Type II-A	vincentii (ATCC 49256)	Fusobacterium nucleatum subsp. nucleatum strain ChDC F317, complete genom	675	675	77%	0	92.26	CP022122.1
Query8	2A-R	Type II-A	vincentii (ATCC 49256)	Fusobacterium nucleatum subsp. nucleatum ChDC F316 strain KCOM 1322, com	675	675	77%	0	92.26	CP012716.1
Query8	2A-R	Type II-A	vincentii (ATCC 49256)	Fusobacterium nucleatum subsp. nucleatum strain KCOM 1250, complete genom	669	669	77%	0	92.05	CP012717.1
Query8	2A-R	Type II-A	vincentii (ATCC 49256)	Fusobacterium nucleatum strain Fn12230 chromosome, complete genome	667	667	81%	0	90.57	CP053468.1
Query8	2A-R	Type II-A	vincentii (ATCC 49256)	Fusobacterium nucleatum subsp. polymorphum strain KCOM 1260 (=ChDC F218	667	667	81%	0	90.57	CP021934.1
Query8	2A-R	Type II-A	vincentii (ATCC 49256)	Fusobacterium nucleatum subsp. polymorphum genome assembly NCTC10562, c	664	664	82%	0	90.12	LN831027.1
Query8	2A-R	Type II-A	vincentii (ATCC 49256)	Fusobacterium nucleatum subsp. polymorphum strain KCOM 1275, complete ger	647	647	77%	0	91.21	CP022123.1
Query8	2A-R	Type II-A	vincentii (ATCC 49256)	Fusobacterium nucleatum subsp. polymorphum strain ChDC F319 genome	647	647	77%	0	91.21	CP013328.1
Query8	2A-R	Type II-A	vincentii (ATCC 49256)	Fusobacterium nucleatum subsp. polymorphum strain ChDC F306, complete ger	647	647	77%	0	91.21	CP013121.1
Query8	2A-R	Type II-A	vincentii (ATCC 49256)	Fusobacterium hwasookii ChDC F206, complete genome	632	632	80%	6.00E-177	89.76	CP013336.1
Query8	2A-R	Type II-A	vincentii (ATCC 49256)	Fusobacterium hwasookii ChDC F300, complete genome	627	627	80%	3.00E-175	89.56	CP013334.1
Query8	2A-R	Type II-A	vincentii (ATCC 49256)	Fusobacterium hwasookii strain KCOM 1253 chromosome, complete genome	627	627	80%	3.00E-175	89.56	CP060115.1
Query8	2A-R	Type II-A	vincentii (ATCC 49256)	Fusobacterium hwasookii strain KCOM 1249 chromosome, complete genome	608	608	77%	1.00E-169	89.75	CP060112.1
Query8	2A-R	Type II-A	vincentii (ATCC 49256)	Fusobacterium hwasookii ChDC F174, complete genome	603	603	77%	5.00E-168	89.54	CP013331.1
Query8	2A-R	Type II-A	vincentii (ATCC 49256)	Fusobacterium pseudoperiodonticum strain KCOM 2653 chromosome, complete	525	525	68%	1.00E-144	89.1	CP024705.1
Query8	2A-R	Type II-A	vincentii (ATCC 49256)	Fusobacterium periodonticum strain 2_1_31 chromosome, complete genome	520	520	67%	5.00E-143	89.18	CP028108.1
Query8	2A-R	Type II-A	vincentii (ATCC 49256)	Fusobacterium pseudoperiodonticum strain KCOM 2305 chromosome, complete	520	520	68%	5.00E-143	88.86	CP024703.1
Query8	2A-R	Type II-A	vincentii (ATCC 49256)	Fusobacterium pseudoperiodonticum strain KCOM 1282 chromosome, complete	520	520	68%	5.00E-143	88.86	CP024702.1
Query8	2A-R	Type II-A	vincentii (ATCC 49256)	Fusobacterium pseudoperiodonticum strain KCOM 1263 chromosome, complete	520	520	68%	5.00E-143	88.86	CP024700.1
Query8	2A-R	Type II-A	vincentii (ATCC 49256)	Fusobacterium pseudoperiodonticum strain KCOM 1283 chromosome, complete	520	520	68%	5.00E-143	88.86	CP024698.1
Query8	2A-R	Type II-A	vincentii (ATCC 49256)	Fusobacterium pseudoperiodonticum strain KCOM 1262 chromosome, complete	520	520	68%	5.00E-143	88.86	CP024731.1
Query8	2A-R	Type II-A	vincentii (ATCC 49256)	Fusobacterium pseudoperiodonticum strain KCOM 2555 chromosome, complete	514	514	68%	2.00E-141	88.63	CP024704.1
Query8	2A-R	Type II-A	vincentii (ATCC 49256)	Fusobacterium pseudoperiodonticum strain KCOM 1277 chromosome, complete	514	514	68%	2.00E-141	88.63	CP024701.1
Query8	2A-R	Type II-A	vincentii (ATCC 49256)	Fusobacterium pseudoperiodonticum strain KCOM 1261 chromosome, complete	514	514	68%	2.00E-141	88.63	CP024699.1
Query8	2A-R	Type II-A	vincentii (ATCC 49256)	Fusobacterium sp. P6 Po strain Fusobacterium gastrosuis sp. nov. CDW1, P6 Po	412	412	69%	9.00E-111	84.19	LT607734.1
Query8	2A-R	Type II-A	vincentii (ATCC 49256)	Fusobacterium gonidiaformans ATCC 25563 chromosome, complete genome	84.2	84.2	20%	8.00E-12	78.95	CP028106.1

Query	Sample_Name	CRISPR subtype	Subsp. (Type strain #)	Description	Max Score	Total Score	Query Cover	E value	Per. ident	Accession
Query9	3A-F	Type III-A	animalis (ATCC 51191)	Fusobacterium nucleatum subsp. animalis strain ChDC F332, complete genome	791	791	100%	0	92.17	CP022124.1
Query9	3A-F	Type III-A	animalis (ATCC 51191)	Fusobacterium nucleatum strain Fn12230 chromosome, complete genome	695	695	99%	0	89.15	CP053468.1
Query9	3A-F	Type III-A	animalis (ATCC 51191)	Fusobacterium hwasookii ChDC F174, complete genome	693	693	100%	0	88.99	CP013331.1
Query9	3A-F	Type III-A	animalis (ATCC 51191)	Fusobacterium hwasookii ChDC F300, complete genome	678	678	99%	0	88.59	CP013334.1
Query9	3A-F	Type III-A	animalis (ATCC 51191)	Fusobacterium hwasookii strain KCOM 1253 chromosome, complete genome	678	678	99%	0	88.59	CP060115.1
Query9	3A-F	Type III-A	animalis (ATCC 51191)	Fusobacterium nucleatum subsp. polymorphum strain KCOM 1260 (=ChDC F218	675	675	99%	0	88.45	CP021934.1
Query9	3A-F	Type III-A	animalis (ATCC 51191)	Fusobacterium hwasookii ChDC F206, complete genome	669	669	99%	0	88.26	CP013336.1
Query9	3A-F	Type III-A	animalis (ATCC 51191)	Fusobacterium nucleatum subsp. polymorphum strain ChDC F306, complete ger	660	660	100%	0	87.99	CP013121.1
Query9	3A-F	Type III-A	animalis (ATCC 51191)	Fusobacterium nucleatum subsp. polymorphum strain ChDC F319 genome	647	647	95%	0	88.5	CP013328.1
Query9	3A-F	Type III-A	animalis (ATCC 51191)	Fusobacterium periodonticum strain 2_1_31 chromosome, complete genome	483	483	76%	6.00E-132	87.07	CP028108.1
Query9	3A-F	Type III-A	animalis (ATCC 51191)	Fusobacterium pseudoperiodonticum strain KCOM 2305 chromosome, complete	449	449	71%	6.00E-122	86.97	CP024703.1

Query	Sample_Name	CRISPR subtype	Subsp. (Type strain #)	Description	Max Score	Total Score	Query Cover	E value	Per. ident	Accession
Query10	3A-R	Type III-A	animalis (ATCC 51191)	Fusobacterium nucleatum subsp. animalis strain ChDC F332, complete genome	1014	1014	100%	0	98.77	CP022124.1
Query10	3A-R	Type III-A	animalis (ATCC 51191)	Fusobacterium nucleatum subsp. animalis 7_1, complete genome	538	538	56%	1.00E-148	96.89	CP007062.1
Query10	3A-R	Type III-A	animalis (ATCC 51191)	Fusobacterium nucleatum subsp. animalis 4_8, complete genome	538	538	56%	1.00E-148	96.89	CP003723.1
Query10	3A-R	Type III-A	animalis (ATCC 51191)	Fusobacterium nucleatum subsp. animalis strain KCOM 1279, complete genome	508	508	52%	1.00E-139	97.02	CP012713.1
Query10	3A-R	Type III-A	animalis (ATCC 51191)	Fusobacterium nucleatum subsp. vincentii strain KCOM 2931 chromosome, comp	505	505	55%	1.00E-138	95.31	CP024749.1
Query10	3A-R	Type III-A	animalis (ATCC 51191)	Fusobacterium nucleatum subsp. nucleatum ATCC 23726 chromosome, complete	429	429	57%	8.00E-116	90.55	CP028109.1
Query10	3A-R	Type III-A	animalis (ATCC 51191)	Fusobacterium nucleatum subsp. nucleatum strain ChDC F317, complete genom	429	429	57%	8.00E-116	90.55	CP022122.1
Query10	3A-R	Type III-A	animalis (ATCC 51191)	Fusobacterium nucleatum subsp. nucleatum ChDC F316 strain KCOM 1322, com	429	429	56%	8.00E-116	90.77	CP012716.1
Query10	3A-R	Type III-A	animalis (ATCC 51191)	Fusobacterium sp. oral taxon 203 genome	401	401	57%	2.00E-107	89.06	CP016200.1
Query10	3A-R	Type III-A	animalis (ATCC 51191)	Fusobacterium nucleatum subsp. nucleatum strain 25586 chromosome, complete	399	399	53%	7.00E-107	90.26	CP028101.1
Query10	3A-R	Type III-A	animalis (ATCC 51191)	Fusobacterium nucleatum subsp. nucleatum ATCC 25586, complete genome	399	399	53%	7.00E-107	90.26	AE009951.2
Query10	3A-R	Type III-A	animalis (ATCC 51191)	Fusobacterium nucleatum subsp. nucleatum strain KCOM 1250, complete genom	394	394	53%	3.00E-105	89.94	CP012717.1
Query10	3A-R	Type III-A	animalis (ATCC 51191)	Fusobacterium nucleatum subsp. animalis strain KCOM 1325, complete genome	374	374	50%	4.00E-99	90.28	CP012715.1
Query10	3A-R	Type III-A	animalis (ATCC 51191)	Fusobacterium nucleatum subsp. vincentii ChDC F8 strain KCOM 1231, complet	370	370	50%	5.00E-98	89.97	CP012714.1
Query10	3A-R	Type III-A	animalis (ATCC 51191)	Fusobacterium nucleatum subsp. vincentii 3_1_27 chromosome 1, complete sequ	370	370	50%	5.00E-98	89.97	CP007064.1
Query10	3A-R	Type III-A	animalis (ATCC 51191)	Fusobacterium nucleatum isolate MGYG-HGUT-01347 genome assembly, chrom	370	370	49%	5.00E-98	90.21	LR698955.1
Query10	3A-R	Type III-A	animalis (ATCC 51191)	Fusobacterium nucleatum strain Fn12230 chromosome, complete genome	350	350	51%	7.00E-92	88.4	CP053468.1
Query10	3A-R	Type III-A	animalis (ATCC 51191)	Fusobacterium nucleatum subsp. polymorphum genome assembly NCTC10562, c	350	350	51%	7.00E-92	88.4	LN831027.1
Query10	3A-R	Type III-A	animalis (ATCC 51191)	Fusobacterium nucleatum subsp. polymorphum strain KCOM 1275, complete ger	322	322	51%	1.00E-83	86.69	CP022123.1
Query10	3A-R	Type III-A	animalis (ATCC 51191)	Fusobacterium hwasookii ChDC F300, complete genome	270	270	50%	5.00E-68	84.14	CP013334.1
Query10	3A-R	Type III-A	animalis (ATCC 51191)	Fusobacterium hwasookii ChDC F206, complete genome	270	270	50%	5.00E-68	84.14	CP013336.1
Query10	3A-R	Type III-A	animalis (ATCC 51191)	Fusobacterium hwasookii strain KCOM 1253 chromosome, complete genome	270	270	50%	5.00E-68	84.14	CP060115.1
Query10	3A-R	Type III-A	animalis (ATCC 51191)	Fusobacterium hwasookii strain KCOM 1249 chromosome, complete genome	235	235	34%	2.00E-57	88	CP060112.1
Query10	3A-R	Type III-A	animalis (ATCC 51191)	Fusobacterium hwasookii ChDC F174, complete genome	230	230	34%	9.00E-56	87.82	CP013331.1

Supplementary Table S5. The validation of PCR amplification by each primer set (Continued)

Database	Whole-Genome shotgun		
BLAST algorith	megablast		
	1B1	Type I-B(1)	animalis JCM11025^{T} (ATCC 51191)
	1B2P	Type I-B(2)	polymorphum JCM12990^{T} (ATCC 10953)
	2A	Type II-A	vincentii JCM11023^{T} (ATCC 49256)
	3A	Type III-A	animalis JCM11025^{T} (ATCC 51191)

Query	Sample_Name	CRISPR subtype	Subsp. (Type strain #)	Description	Max Scor	Total Scor	Query Cove	E value	Per. iden	Accession
Query1	1B1-F	Type I-B(1)	animalis (ATCC 51191)	Fusobacterium nucleatum subsp. animalis ATCC 51191 contig00001, whole genor	1107	7664	100%	0	100	AFQD01000001.1
Query1	1B1-F	Type I-B(1)	animalis (ATCC 51191)	Fusobacterium nucleatum isolate MGYG-HGUT-01326 genome assembly, contig	627	1784	98%	2.00E-178	86.12	CABKNP010000002.1
Query1	1B1-F	Type I-B(1)	animalis (ATCC 51191)	Fusobacterium nucleatum subsp. animalis 7_1 cont1.3, whole genome shotgun se	627	1784	98%	2.00E-178	86.12	AKBT01000003.1
Query1	1B1-F	Type I-B(1)	animalis (ATCC 51191)	Fusobacterium nucleatum subsp. polymorphum F0401 supercont2.1, whole genor	627	1784	98%	2.00E-178	86.12	ADDB02000001.1
Query1	1B1-F	Type I-B(1)	animalis (ATCC 51191)	Fusobacterium nucleatum subsp. animalis 3_1_33 cont2.16, whole genome shotgu	621	621	97%	8.00E-177	86.09	ACQE02000016.1
Query1	1B1-F	Type I-B(1)	animalis (ATCC 51191)	Fusobacterium nucleatum subsp. animalis F0419 cont1.1, whole genome shotgun	612	1610	100%	5.00E-174	85.76	AKCE01000001.1
Query1	1B1-F	Type I-B(1)	animalis (ATCC 51191)	Fusobacterium nucleatum strain UMB0249 .16933_8_42.11, whole genome shotgu	595	6745	99%	5.00E-169	85.22	PNHC01000011.1
Query1	1B1-F	Type I-B(1)	animalis (ATCC 51191)	Fusobacterium nucleatum subsp. animalis ChDC F324 Contig002, whole genome	588	879	100%	8.00E-167	89.96	ATKD01000002.1
Query1	1B1-F	Type I-B(1)	animalis (ATCC 51191)	Fusobacterium nucleatum subsp. animalis 11_3_2 cont1.48, whole genome shotgu	584	1346	98%	1.00E-165	89.85	ACUO01000048.1
Query1	1B1-F	Type I-B(1)	animalis (ATCC 51191)	Fusobacterium sp. CAG:649 WGS project CBBC01000000 data, contig, whole gen	575	575	76%	6.00E-163	89.29	CBBC010000031.1
Query1	1B1-F	Type I-B(1)	animalis (ATCC 51191)	Fusobacterium nucleatum CTI-1 acUTP-supercont1.8.C28, whole genome shotgu	568	842	97%	1.00E-160	92.89	AXNZ01000028.1
Query1	1B1-F	Type I-B(1)	animalis (ATCC 51191)	Fusobacterium nucleatum CTI-3 acUTO-supercont1.4.C9, whole genome shotgun	568	568	54%	1.00E-160	97.87	AXNX01000009.1
Query1	1B1-F	Type I-B(1)	animalis (ATCC 51191)	Fusobacterium nucleatum subsp. animalis F0419 strain OT 420 cont1.18, whole g	564	739	86%	1.00E-159	92.66	AGEH01000018.1
Query1	1B1-F	Type I-B(1)	animalis (ATCC 51191)	Fusobacterium nucleatum subsp. polymorphum strain KCOM 1001 F119_1, whol	558	3191	99%	7.00E-158	83.99	NHRT01000001.1
Query1	1B1-F	Type I-B(1)	animalis (ATCC 51191)	Fusobacterium sp. CM1 ctg7180000008816, whole genome shotgun sequence	536	1057	98%	3.00E-151	96.05	JANB01000091.1
Query1	1B1-F	Type I-B(1)	animalis (ATCC 51191)	Fusobacterium nucleatum subsp. polymorphum strain KCOM 1002 (=ChDC F175	532	6565	100%	4.00E-150	83.44	NIRJ01000001.1
Query1	1B1-F	Type I-B(1)	animalis (ATCC 51191)	Fusobacterium nucleatum strain MJR7757B Fusobacterium_spHMPREF3221-1.0	532	532	54%	4.00E-150	96.02	LRPY01000274.1
Query1	1B1-F	Type I-B(1)	animalis (ATCC 51191)	Fusobacterium sp. HMSC065F01 Fusobacterium_spHMPREF2931-1.0_Cont816.8	529	760	76%	5.00E-149	95.47	LTWB01000128.1
Query1	1B1-F	Type I-B(1)	animalis (ATCC 51191)	Fusobacterium sp. CM22 ctg7180000007337, whole genome shotgun sequence	521	5028	100%	9.00E-147	83.2	JAQC01000015.1
Query1	1B1-F	Type I-B(1)	animalis (ATCC 51191)	Fusobacterium nucleatum CTI-5 acUTW-supercont1.24.C34, whole genome shotg	457	457	45%	2.00E-127	96.72	AXNW01000034.1
Query1	1B1-F	Type I-B(1)	animalis (ATCC 51191)	Fusobacterium nucleatum subsp. animalis 3_1_33 cont2.18, whole genome shotgu	178	4440	50%	2.00E-43	78.5	ACQE02000018.1
Query1	1B1-F	Type I-B(1)	animalis (ATCC 51191)	Fusobacterium sp. CM1 ctg7180000008828, whole genome shotgun sequence	147	3136	51%	6.00E-34	76.8	JANB01000087.1
Query1	1B1-F	Type I-B(1)	animalis (ATCC 51191)	Fusobacterium sp. HMSC065F01 Fusobacterium_spHMPREF2931-1.0_Cont816.4	141	1372	51%	3.00E-32	76.05	LTWB01000124.1
Query1	1B1-F	Type I-B(1)	animalis (ATCC 51191)	Fusobacterium sp. CM22 ctg7180000007336_2, whole genome shotgun sequence	141	1489	50%	3.00E-32	76.13	JAQC01000003.1
Query1	1B1-F	Type I-B(1)	animalis (ATCC 51191)	Fusobacterium sp. HMSC065F01 Fusobacterium_spHMPREF2931-1.0_Cont816.6	135	1165	50%	1.00E-30	76.14	LTWB01000126.1
Query1	1B1-F	Type I-B(1)	animalis (ATCC 51191)	Fusobacterium sp. HMSC065F01 Fusobacterium_spHMPREF2931-1.0_Cont816.1	135	1011	51%	1.00E-30	78.57	LTWB01000119.1
Query1	1B1-F	Type I-B(1)	animalis (ATCC 51191)	Fusobacterium sp. HMSC065F01 Fusobacterium_spHMPREF2931-1.0_Cont816.5	134	1166	50%	5.00E-30	78.48	LTWB01000125.1
Query1	1B1-F	Type I-B(1)	animalis (ATCC 51191)	Fusobacterium nucleatum subsp. animalis strain KCOM 1280 (= ChDC F318) F31	132	573	51%	2.00E-29	75.39	NJGJ01000001.1
Query1	1B1-F	Type I-B(1)	animalis (ATCC 51191)	Fusobacterium sp. HMSC065F01 Fusobacterium_spHMPREF2931-1.0_Cont816.7	130	982	49%	6.00E-29	75.5	LTWB01000127.1
Query1	1B1-F	Type I-B(1)	animalis (ATCC 51191)	Fusobacterium sp. CM22 ctg7180000007336_1, whole genome shotgun sequence	130	1115	51%	6.00E-29	75.66	JAQC01000020.1
Query1	1B1-F	Type I-B(1)	animalis (ATCC 51191)	Fusobacterium nucleatum subsp. animalis F0419 strain OT 420 cont1.17, whole g	119	357	48%	1.00E-25	75.24	AGEH01000017.1
Query1	1B1-F	Type I-B(1)	animalis (ATCC 51191)	Fusobacterium sp. CM1 ctg7180000008832, whole genome shotgun sequence	117	671	50%	5.00E-25	75	JANB01000093.1
Query1	1B1-F	Type I-B(1)	animalis (ATCC 51191)	Fusobacterium sp. HMSC065F01 Fusobacterium_spHMPREF2931-1.0_Cont816.2	108	1119	50%	3.00E-22	79.76	LTWB01000122.1
Query1	1B1-F	Type I-B(1)	animalis (ATCC 51191)	Fusobacterium sp. HMSC065F01 Fusobacterium_spHMPREF2931-1.0_Cont816.3	106	733	51%	1.00E-21	74.6	LTWB01000123.1
Query1	1B1-F	Type I-B(1)	animalis (ATCC 51191)	Fusobacterium nucleatum subsp. nucleatum strain ChDC F311 F311_contig0000	69.4	135	12%	1.00E-10	100	LMVH01000001.1
Query1	1B1-F	Type I-B(1)	animalis (ATCC 51191)	Fusobacterium hwasookii ChDC F300 Contig091, whole genome shotgun sequenc	69.4	69.4	6%	1.00E-10	100	ATKG01000091.1
Query	Sample_Name	CRISPR subtype	Subsp. (Type strain #)	Description	Max Score	Total Score	Query Cover	E value	Per. ident	Accession
Query2	1B1-R	Type I-B(1)	animalis (ATCC 51191)	Fusobacterium nucleatum subsp. animalis ATCC 51191 contig00001, whole genor	1053	5598	100%	0	100	AFQD01000001.1
Query2	1B1-R	Type I-B(1)	animalis (ATCC 51191)	Fusobacterium sp. CM1 ctg7180000008832, whole genome shotgun sequence	580	1365	94%	1.00E-164	89.43	JANB01000093.1
Query2	1B1-R	Type I-B(1)	animalis (ATCC 51191)	Fusobacterium nucleatum subsp. animalis strain KCOM 1280 (= ChDC F318) F31	575	1050	92%	6.00E-163	86.72	NJGJ01000001.1
Query2	1B1-R	Type I-B(1)	animalis (ATCC 51191)	Fusobacterium nucleatum isolate MGYG-HGUT-01326 genome assembly, contig	575	1261	92%	6.00E-163	86.54	CABKNP010000002.1
Query2	1B1-R	Type I-B(1)	animalis (ATCC 51191)	Fusobacterium nucleatum subsp. animalis 7_1 cont1.3, whole genome shotgun se	575	1261	92%	6.00E-163	86.54	AKBT01000003.1
Query2	1B1-R	Type I-B(1)	animalis (ATCC 51191)	Fusobacterium nucleatum subsp. polymorphum F0401 supercont2.1, whole genor	575	1261	92%	6.00E-163	86.54	ADDB02000001.1
Query2	1B1-R	Type I-B(1)	animalis (ATCC 51191)	Fusobacterium nucleatum subsp. animalis F0419 cont1.1, whole genome shotgun	562	1259	93%	5.00E-159	88.77	AKCE01000001.1
Query2	1B1-R	Type I-B(1)	animalis (ATCC 51191)	Fusobacterium nucleatum subsp. animalis F0419 strain OT 420 cont1.17, whole g	562	562	80%	5.00E-159	88.91	AGEH01000017.1
Query2	1B1-R	Type I-B(1)	animalis (ATCC 51191)	Fusobacterium sp. CAG:649 WGS project CBBC01000000 data, contig, whole gen	560	560	81%	2.00E-158	88.49	CBBC010000122.1
Query2	1B1-R	Type I-B(1)	animalis (ATCC 51191)	Fusobacterium sp. HMSC065F01 Fusobacterium_spHMPREF2931-1.0_Cont816.1	556	1226	93%	2.00E-157	88.84	LTWB01000119.1
Query2	1B1-R	Type I-B(1)	animalis (ATCC 51191)	Fusobacterium nucleatum CTI-3 acUTO-supercont1.3.C8, whole genome shotgun	556	768	94%	2.00E-157	86.09	AXNX01000008.1
Query2	1B1-R	Type I-B(1)	animalis (ATCC 51191)	Fusobacterium nucleatum subsp. animalis 11_3_2 cont1.48, whole genome shotgu	556	3530	92%	2.00E-157	88.7	ACUO01000048.1
Query2	1B1-R	Type I-B(1)	animalis (ATCC 51191)	Fusobacterium nucleatum CTI-1 acUTP-supercont1.5.C25, whole genome shotgu	547	673	70%	1.00E-154	96.67	AXNZ01000025.1
Query2	1B1-R	Type I-B(1)	animalis (ATCC 51191)	Fusobacterium nucleatum subsp. animalis ChDC F324 Contig002, whole genome	547	786	75%	1.00E-154	96.67	ATKD01000002.1
Query2	1B1-R	Type I-B(1)	animalis (ATCC 51191)	Fusobacterium nucleatum strain MJR7757B Fusobacterium_spHMPREF3221-1.0	545	545	69%	5.00E-154	91.73	LRPY01000066.1
Query2	1B1-R	Type I-B(1)	animalis (ATCC 51191)	Fusobacterium nucleatum subsp. animalis 3_1_33 cont2.18, whole genome shotgu	540	2923	88%	2.00E-152	95.82	ACQE02000018.1
Query2	1B1-R	Type I-B(1)	animalis (ATCC 51191)	Fusobacterium nucleatum CTI-5 acUTW-supercont1.31.C42, whole genome shotg	534	534	57%	1.00E-150	95.78	AXNW01000042.1
Query2	1B1-R	Type I-B(1)	animalis (ATCC 51191)	Fusobacterium nucleatum strain UMB0249 .16933_8_42.11, whole genome shotgu	527	4462	100%	2.00E-148	90.84	PNHC01000011.1
Query2	1B1-R	Type I-B(1)	animalis (ATCC 51191)	Fusobacterium nucleatum subsp. animalis strain P2_CP NODE_6_length_82368_	300	300	28%	4.00E-80	99.39	NPND01000006.1
Query2	1B1-R	Type I-B(1)	animalis (ATCC 51191)	Fusobacterium nucleatum subsp. animalis strain P2_LM NODE_70_length_9528_	300	300	28%	4.00E-80	99.39	NPNC01000070.1
Query2	1B1-R	Type I-B(1)	animalis (ATCC 51191)	Fusobacterium nucleatum subsp. animalis 21_1A cont3.2, whole genome shotgun	300	300	28%	4.00E-80	99.39	ADEE02000002.1
Query2	1B1-R	Type I-B(1)	animalis (ATCC 51191)	Fusobacterium nucleatum subsp. animalis D11 adfWA-supercont2.1.C11, whole g	300	300	28%	4.00E-80	99.39	ACDS02000011.1
Query2	1B1-R	Type I-B(1)	animalis (ATCC 51191)	Fusobacterium sp. HMSC064B11 Fusobacterium_spHMPREF3051-1.0_Cont40.1,	137	137	19%	3.00E-31	89.19	LTSL01000097.1
Query2	1B1-R	Type I-B(1)	animalis (ATCC 51191)	Fusobacterium nucleatum subsp. polymorphum strain KCOM 1001 F119_1, whol	124	1703	48%	3.00E-27	80.59	NHRT01000001.1
Query2	1B1-R	Type I-B(1)	animalis (ATCC 51191)	Fusobacterium sp. CM1 ctg7180000008828, whole genome shotgun sequence	124	1962	39%	3.00E-27	80.47	JANB01000087.1
Query2	1B1-R	Type I-B(1)	animalis (ATCC 51191)	Fusobacterium nucleatum subsp. animalis 3_1_33 cont2.16, whole genome shotgu	122	5690	42%	1.00E-26	81.18	ACQE02000016.1
Query2	1B1-R	Type I-B(1)	animalis (ATCC 51191)	Fusobacterium nucleatum subsp. animalis 3_1_33 cont2.17, whole genome shotgu	119	2871	40%	1.00E-25	80.84	ACQE02000017.1
Query2	1B1-R	Type I-B(1)	animalis (ATCC 51191)	Fusobacterium sp. CM22 ctg7180000007337, whole genome shotgun sequence	108	2718	39%	3.00E-22	79.41	JAQC01000015.1
Query2	1B1-R	Type I-B(1)	animalis (ATCC 51191)	Fusobacterium nucleatum subsp. polymorphum strain KCOM 1002 (=ChDC F175	104	3683	41%	3.00E-21	78.95	NIRJ01000001.1
Query2	1B1-R	Type I-B(1)	animalis (ATCC 51191)	Fusobacterium sp. HMSC064B12 Fusobacterium_spHMPREF2775-1.0_Cont549.1	104	104	21%	3.00E-21	82.4	LTGW01000093.1
Query2	1B1-R	Type I-B(1)	animalis (ATCC 51191)	Fusobacterium sp. CM22 ctg7180000007336_1, whole genome shotgun sequence	104	667	30%	3.00E-21	85.58	JAQC01000020.1
Query2	1B1-R	Type I-B(1)	animalis (ATCC 51191)	Fusobacterium sp. CM1 ctg7180000008816, whole genome shotgun sequence	100	2406	40%	5.00E-20	78.57	JANB01000091.1
Query2	1B1-R	Type I-B(1)	animalis (ATCC 51191)	Fusobacterium sp. HMSC065F01 Fusobacterium_spHMPREF2931-1.0_Cont816.7	99	519	39%	2.00E-19	78.57	LTWB01000127.1
Query2	1B1-R	Type I-B(1)	animalis (ATCC 51191)	Fusobacterium sp. CM22 ctg7180000007336_2, whole genome shotgun sequence	97.1	901	40%	6.00E-19	75.22	JAQC01000003.1
Query2	1B1-R	Type I-B(1)	animalis (ATCC 51191)	Fusobacterium sp. HMSC065F01 Fusobacterium_spHMPREF2931-1.0_Cont816.5	93.5	661	39%	8.00E-18	77.71	LTWB01000125.1
Query2	1B1-R	Type I-B(1)	animalis (ATCC 51191)	Fusobacterium sp. HMSC065F01 Fusobacterium_spHMPREF2931-1.0_Cont816.4	93.5	776	29%	8.00E-18	77.84	LTWB01000124.1
Query2	1B1-R	Type I-B(1)	animalis (ATCC 51191)	Fusobacterium nucleatum W1481 Contig37, whole genome shotgun sequence	91.6	91.6	10%	3.00E-17	93.44	AXUR01000037.1
Query2	1B1-R	Type I-B(1)	animalis (ATCC 51191)	Fusobacterium sp. HMSC065F01 Fusobacterium_spHMPREF2931-1.0_Cont816.6	89.8	687	29%	1.00E-16	83.51	LTWB01000126.1
Query2	1B1-R	Type I-B(1)	animalis (ATCC 51191)	Fusobacterium sp. HMSC065F01 Fusobacterium_spHMPREF2931-1.0_Cont816.3	84.2	352	38%	5.00E-15	76.47	LTWB01000123.1
Query2	1B1-R	Type I-B(1)	animalis (ATCC 51191)	Fusobacterium sp. HMSC065F01 Fusobacterium_spHMPREF2931-1.0_Cont816.2	80.5	535	30%	6.00E-14	76.57	LTWB01000122.1
Query2	1B1-R	Type I-B(1)	animalis (ATCC 51191)	Fusobacterium nucleatum subsp. animalis F0419 strain OT 420 cont1.18, whole g	63.9	122	28%	6.00E-09	79	AGEH01000018.1
Query2	1B1-R	Type I-B(1)	animalis (ATCC 51191)	Fusobacterium nucleatum CTI-3 acUTO-supercont1.4.C9, whole genome shotgun	60.2	118	11%	8.00E-08	100	AXNX01000009.1
Query2	1B1-R	Type I-B(1)	animalis (ATCC 51191)	Fusobacterium sp. HMSC065F01 Fusobacterium_spHMPREF2931-1.0_Cont816.8	56.5	56.5	5%	1.00E-06	100	LTWB01000128.1
Query2	1B1-R	Type I-B(1)	animalis (ATCC 51191)	Fusobacterium nucleatum CTI-1 acUTP-supercont1.8.C28, whole genome shotgu	56.5	113	5%	1.00E-06	100	AXNZ01000028.1

Supplementary Table S5. The validation of PCR amplification by each primer set (Continued)

Query	Sample_Name	CRISPR subtype	Subsp. (Type strain #)	Description	Max Score	Total Score	Query Cover	E value	Per. ident	Accession
Query3	1B2N-F	Type I-B(2)	nucleatum (ATCC 25586)	Fusobacterium nucleatum subsp. nucleatum ChDC F316 Contig049, whole genom	466	1474	100%	4.00E-130	81.03	ATKC01000049.1
Query3	1B2N-F	Type I-B(2)	nucleatum (ATCC 25586)	Fusobacterium nucleatum subsp. nucleatum ATCC 23726 contig00035, whole gen	438	3618	100%	9.00E-122	82.98	ADVK01000030.1
Query3	1B2N-F	Type I-B(2)	nucleatum (ATCC 25586)	Fusobacterium nucleatum CTI-6 addSJ-supercont1.12, whole genome shotgun se	436	2722	100%	3.00E-121	80.91	AXNV01000012.1
Query3	1B2N-F	Type I-B(2)	nucleatum (ATCC 25586)	Fusobacterium nucleatum subsp. nucleatum strain ChDC F311 F311_contig0000	435	6920	93%	1.00E-120	81	LMVH01000001.1
Query3	1B2N-F	Type I-B(2)	nucleatum (ATCC 25586)	Fusobacterium hwasookii ChDC F174 Contig035, whole genome shotgun sequenc	435	6373	100%	1.00E-120	81.06	ATKF01000035.1
Query3	1B2N-F	Type I-B(2)	nucleatum (ATCC 25586)	Fusobacterium sp. oral taxon 370 str. F0437 F_sporaltaxon370F0437-1.0_Cont284	424	1370	92%	3.00E-117	80.81	AGAD01000088.1
Query3	1B2N-F	Type I-B(2)	nucleatum (ATCC 25586)	Fusobacterium nucleatum strain OH5060 scaffold_0, whole genome shotgun sequ	414	7756	100%	2.00E-114	80.44	RQZD01000001.1
Query3	1B2N-F	Type I-B(2)	nucleatum (ATCC 25586)	Fusobacterium nucleatum strain 12230 NODE_1_length_386863_cov_114.933, wh	412	2329	94%	6.00E-114	80.41	QKOC01000001.1
Query3	1B2N-F	Type I-B(2)	nucleatum (ATCC 25586)	Fusobacterium nucleatum strain 12230 MIT 2016 NODE_17_length_36103_cov_2	412	2329	94%	6.00E-114	80.41	NXMD01000017.1
Query3	1B2N-F	Type I-B(2)	nucleatum (ATCC 25586)	Fusobacterium nucleatum subsp. polymorphum strain KCOM 1257 (=ChDC F186	412	8594	100%	6.00E-114	80.51	NIRL01000001.1
Query3	1B2N-F	Type I-B(2)	nucleatum (ATCC 25586)	TPA_asm: Fusobacterium sp. isolate UBA8871 contig_2885, whole genome shotgu	412	2329	93%	6.00E-114	80.41	DPCP01000050.1
Query3	1B2N-F	Type I-B(2)	nucleatum (ATCC 25586)	Fusobacterium nucleatum subsp. polymorphum strain KCOM 1274 (=ChDC F309	411	4483	100%	2.00E-113	80.2	NIRO01000001.1
Query3	1B2N-F	Type I-B(2)	nucleatum (ATCC 25586)	Fusobacterium nucleatum subsp. polymorphum ATCC 10953 Ctg92, whole genom	405	4241	100%	9.00E-112	80.07	AARG01000014.1
Query3	1B2N-F	Type I-B(2)	nucleatum (ATCC 25586)	Fusobacterium hwasookii ChDC F128 contig00003, whole genome shotgun seque	403	7216	100%	3.00E-111	80.07	ALVD01000003.1
Query3	1B2N-F	Type I-B(2)	nucleatum (ATCC 25586)	Fusobacterium sp. OBRC1 ctgN370L120013C40.9385, whole genome shotgun sequ	398	9084	93%	2.00E-109	79.97	JANA01000075.1
Query3	1B2N-F	Type I-B(2)	nucleatum (ATCC 25586)	Fusobacterium nucleatum subsp. polymorphum strain KCOM 1265 contig1, whol	396	4216	92%	6.00E-109	79.86	PEQW01000001.1
Query3	1B2N-F	Type I-B(2)	nucleatum (ATCC 25586)	Fusobacterium nucleatum subsp. polymorphum strain KCOM 1248 (=ChDC F113	396	4216	93%	6.00E-109	79.86	NIRK01000001.1
Query3	1B2N-F	Type I-B(2)	nucleatum (ATCC 25586)	Fusobacterium nucleatum subsp. polymorphum strain KCOM 1267(=ChDC F290)	392	3070	93%	7.00E-108	79.69	NIRM01000005.1
Query3	1B2N-F	Type I-B(2)	nucleatum (ATCC 25586)	Fusobacterium nucleatum 13_3C adlCx-supercont1.1.C11, whole genome shotgun	390	6841	100%	3.00E-107	78.85	JAOZ01000011.1
Query3	1B2N-F	Type I-B(2)	nucleatum (ATCC 25586)	Fusobacterium nucleatum isolate MGYG-HGUT-01464 genome assembly, contig	390	6841	100%	3.00E-107	78.85	CABKTD010000001.1
Query3	1B2N-F	Type I-B(2)	nucleatum (ATCC 25586)	Fusobacterium hwasookii ChDC F300 Contig133, whole genome shotgun sequenc	390	5834	100%	3.00E-107	83.22	ATKG01000133.1
Query3	1B2N-F	Type I-B(2)	nucleatum (ATCC 25586)	Fusobacterium hwasookii ChDC F145 Contig141, whole genome shotgun sequenc	390	5479	100%	3.00E-107	79.59	ATKE01000141.1
Query3	1B2N-F	Type I-B(2)	nucleatum (ATCC 25586)	Fusobacterium canifelinum strain OH4460_COT-188 scaffold_1, whole genome sh	383	3608	100%	4.00E-105	79.37	RQYY01000002.1
Query3	1B2N-F	Type I-B(2)	nucleatum (ATCC 25586)	Fusobacterium sp. CM22 ctg7180000007352, whole genome shotgun sequence	370	817	86%	3.00E-101	88.89	JAQC01000070.1
Query3	1B2N-F	Type I-B(2)	nucleatum (ATCC 25586)	Fusobacterium sp. HMSC064B11 Fusobacterium_spHMPREF3051-1.0_Cont666.2	355	887	91%	1.00E-96	84.45	LTSL01000151.1
Query3	1B2N-F	Type I-B(2)	nucleatum (ATCC 25586)	Fusobacterium nucleatum isolate MGYG-HGUT-01459 genome assembly, contig	339	339	32%	1.00E-91	97.01	CABKSX010000028.1
Query3	1B2N-F	Type I-B(2)	nucleatum (ATCC 25586)	Fusobacterium nucleatum CTI-2 addTD-supercont1.34.C57, whole genome shotgu	339	339	32%	1.00E-91	97.01	AXNY01000057.1
Query3	1B2N-F	Type I-B(2)	nucleatum (ATCC 25586)	Fusobacterium nucleatum subsp. polymorphum strain KCOM 1001 F119_1, whol	215	3700	92%	2.00E-54	76.17	NHRT01000001.1
Query3	1B2N-F	Type I-B(2)	nucleatum (ATCC 25586)	Fusobacterium nucleatum subsp. nucleatum ChDC F316 Contig050, whole genom	171	1596	61%	4.00E-41	76.21	ATKC01000050.1
Query3	1B2N-F	Type I-B(2)	nucleatum (ATCC 25586)	Fusobacterium sp. HMSC064B11 Fusobacterium_spHMPREF3051-1.0_Cont666.5	163	3188	67%	6.00E-39	75.79	LTSL01000154.1
Query3	1B2N-F	Type I-B(2)	nucleatum (ATCC 25586)	Fusobacterium sp. oral taxon 370 str. F0437 F_sporaltaxon370F0437-1.0_Cont132	159	1638	61%	8.00E-38	75.58	AGAD01000274.1
Query3	1B2N-F	Type I-B(2)	nucleatum (ATCC 25586)	Fusobacterium sp. HMSC064B11 Fusobacterium_spHMPREF3051-1.0_Cont666.4	156	1522	67%	1.00E-36	77.29	LTSL01000153.1
Query3	1B2N-F	Type I-B(2)	nucleatum (ATCC 25586)	Fusobacterium sp. HMSC064B11 Fusobacterium_spHMPREF3051-1.0_Cont666.3	148	3300	60%	2.00E-34	75.07	LTSL01000152.1
Query3	1B2N-F	Type I-B(2)	nucleatum (ATCC 25586)	Fusobacterium hwasookii ChDC F174 Contig036, whole genome shotgun sequenc	148	1792	67%	2.00E-34	75.26	ATKF01000036.1
Query3	1B2N-F	Type I-B(2)	nucleatum (ATCC 25586)	Fusobacterium sp. oral taxon 370 str. F0437 F_sporaltaxon370F0437-1.0_Cont132	135	1309	60%	1.00E-30	74.61	AGAD01000270.1
Query3	1B2N-F	Type I-B(2)	nucleatum (ATCC 25586)	Fusobacterium sp. oral taxon 370 str. F0437 F_sporaltaxon370F0437-1.0_Cont132	126	941	59%	8.00E-28	77.59	AGAD01000271.1
Query3	1B2N-F	Type I-B(2)	nucleatum (ATCC 25586)	Fusobacterium sp. oral taxon 370 str. F0437 F_sporaltaxon370F0437-1.0_Cont132	126	1282	67%	8.00E-28	73.95	AGAD01000269.1
Query3	1B2N-F	Type I-B(2)	nucleatum (ATCC 25586)	Fusobacterium sp. oral taxon 370 str. F0437 F_sporaltaxon370F0437-1.0_Cont132	126	923	59%	8.00E-28	77.5	AGAD01000267.1
Query3	1B2N-F	Type I-B(2)	nucleatum (ATCC 25586)	Fusobacterium sp. oral taxon 370 str. F0437 F_sporaltaxon370F0437-1.0_Cont132	122	892	59%	1.00E-26	75.16	AGAD01000273.1
Query3	1B2N-F	Type I-B(2)	nucleatum (ATCC 25586)	Fusobacterium sp. oral taxon 370 str. F0437 F_sporaltaxon370F0437-1.0_Cont132	122	746	59%	1.00E-26	80.92	AGAD01000268.1
Query3	1B2N-F	Type I-B(2)	nucleatum (ATCC 25586)	Fusobacterium sp. HMSC064B11 Fusobacterium_spHMPREF3051-1.0_Cont666.6	119	1682	67%	1.00E-25	75.16	LTSL01000155.1
Query3	1B2N-F	Type I-B(2)	nucleatum (ATCC 25586)	Fusobacterium nucleatum subsp. polymorphum strain KCOM 1330 (=ChDC F330	104	605	59%	4.00E-21	75.97	NIRQ01000001.1
Query3	1B2N-F	Type I-B(2)	nucleatum (ATCC 25586)	Fusobacterium sp. oral taxon 370 str. F0437 F_sporaltaxon370F0437-1.0_Cont132	100	642	67%	5.00E-20	75.53	AGAD01000272.1
Query3	1B2N-F	Type I-B(2)	nucleatum (ATCC 25586)	Fusobacterium sp. oral taxon 370 str. F0437 F_sporaltaxon370F0437-1.0_Cont132	87.9	570	68%	4.00E-16	77.01	AGAD01000266.1
Query3	1B2N-F	Type I-B(2)	nucleatum (ATCC 25586)	Fusobacterium sp. CM22 ctg7180000007323, whole genome shotgun sequence	76.8	670	58%	8.00E-13	74.58	JAQC01000063.1
Query3	1B2N-F	Type I-B(2)	nucleatum (ATCC 25586)	Fusobacterium sp. CAG:649 WGS project CBBC01000000 data, contig, whole gen	75	138	11%	3.00E-12	100	CBBC010000033.1
Query3	1B2N-F	Type I-B(2)	nucleatum (ATCC 25586)	Fusobacterium nucleatum 13-08-02 DNA, NODE_27, whole genome shotgun sequ	69.4	69.4	7%	1.00E-10	95.45	BHYR01000025.1
Query3	1B2N-F	Type I-B(2)	nucleatum (ATCC 25586)	Fusobacterium nucleatum isolate MGYG-HGUT-01459 genome assembly, contig	58.4	335	31%	3.00E-07	94.74	CABKSX010000027.1
Query3	1B2N-F	Type I-B(2)	nucleatum (ATCC 25586)	Fusobacterium nucleatum CTI-2 addTD-supercont1.33.C56, whole genome shotgu	58.4	335	31%	3.00E-07	94.74	AXNY01000056.1
Query	Sample_Name	CRISPR subtype	Subsp. (Type strain #)	Description	Max Score	Total Score	Query Cover	E value	Per. ident	Accession
Query4	1B2N-R	Type I-B(2)	nucleatum (ATCC 25586)	Fusobacterium nucleatum subsp. nucleatum ChDC F316 Contig050, whole genom	1044	1044	100%	0	97.42	ATKC01000050.1
Query4	1B2N-R	Type I-B(2)	nucleatum (ATCC 25586)	Fusobacterium nucleatum subsp. nucleatum strain ChDC F311 F311_contig0000	1038	1038	100%	0	97.26	LMVH01000001.1
Query4	1B2N-R	Type I-B(2)	nucleatum (ATCC 25586)	Fusobacterium nucleatum isolate MGYG-HGUT-01459 genome assembly, contig	1038	1038	100%	0	97.26	CABKSX010000027.1
Query4	1B2N-R	Type I-B(2)	nucleatum (ATCC 25586)	Fusobacterium nucleatum CTI-2 addTD-supercont1.33.C56, whole genome shotgu	1038	1038	100%	0	97.26	AXNY01000056.1
Query4	1B2N-R	Type I-B(2)	nucleatum (ATCC 25586)	Fusobacterium nucleatum subsp. nucleatum ATCC 23726 contig00035, whole gen	1038	1038	100%	0	97.26	ADVK01000030.1
Query4	1B2N-R	Type I-B(2)	nucleatum (ATCC 25586)	Fusobacterium nucleatum CTI-6 addSJ-supercont1.12, whole genome shotgun se	296	296	41%	6.00E-79	87.64	AXNV01000012.1
Query4	1B2N-R	Type I-B(2)	nucleatum (ATCC 25586)	Fusobacterium canifelinum strain OH4460_COT-188 scaffold_1, whole genome sh	291	291	46%	3.00E-77	85.07	RQYY01000002.1
Query4	1B2N-R	Type I-B(2)	nucleatum (ATCC 25586)	Fusobacterium nucleatum subsp. polymorphum ATCC 10953 Ctg92, whole genom	291	291	41%	3.00E-77	87.26	AARG01000014.1
Query4	1B2N-R	Type I-B(2)	nucleatum (ATCC 25586)	Fusobacterium nucleatum subsp. polymorphum strain KCOM 1330 (=ChDC F330	285	285	41%	1.00E-75	86.87	NIRQ01000001.1
Query4	1B2N-R	Type I-B(2)	nucleatum (ATCC 25586)	Fusobacterium sp. CM22 ctg7180000007323, whole genome shotgun sequence	285	285	41%	1.00E-75	86.87	JAQC01000063.1
Query4	1B2N-R	Type I-B(2)	nucleatum (ATCC 25586)	Fusobacterium nucleatum subsp. polymorphum strain KCOM 1278 (=ChDC F313	283	283	41%	5.00E-75	86.77	NIRP01000004.1
Query4	1B2N-R	Type I-B(2)	nucleatum (ATCC 25586)	Fusobacterium nucleatum subsp. polymorphum strain KCOM 1271 (=ChDC F305	283	283	41%	5.00E-75	86.77	NIRN01000001.1
Query4	1B2N-R	Type I-B(2)	nucleatum (ATCC 25586)	Fusobacterium nucleatum strain 12230 NODE_1_length_386863_cov_114.933, wh	279	279	41%	6.00E-74	86.49	QKOC01000001.1
Query4	1B2N-R	Type I-B(2)	nucleatum (ATCC 25586)	Fusobacterium nucleatum subsp. polymorphum strain KCOM 1265 contig1, whol	279	279	41%	6.00E-74	86.43	PEQW01000001.1
Query4	1B2N-R	Type I-B(2)	nucleatum (ATCC 25586)	Fusobacterium nucleatum strain 12230 MIT 2016 NODE_17_length_36103_cov_2	279	279	41%	6.00E-74	86.49	NXMD01000017.1
Query4	1B2N-R	Type I-B(2)	nucleatum (ATCC 25586)	Fusobacterium nucleatum subsp. polymorphum strain KCOM 1248 (=ChDC F113	279	279	41%	6.00E-74	86.43	NIRK01000001.1
Query4	1B2N-R	Type I-B(2)	nucleatum (ATCC 25586)	Fusobacterium nucleatum subsp. polymorphum strain KCOM 1001 F119_1, whol	279	279	41%	6.00E-74	86.49	NHRT01000001.1
Query4	1B2N-R	Type I-B(2)	nucleatum (ATCC 25586)	Fusobacterium nucleatum 13_3C adlCx-supercont1.1.C11, whole genome shotgun	279	279	41%	6.00E-74	86.49	JAOZ01000011.1
Query4	1B2N-R	Type I-B(2)	nucleatum (ATCC 25586)	Fusobacterium sp. OBRC1 ctgN370L120013C40.9385, whole genome shotgun sequ	279	279	41%	6.00E-74	86.49	JANA01000075.1
Query4	1B2N-R	Type I-B(2)	nucleatum (ATCC 25586)	TPA_asm: Fusobacterium sp. isolate UBA8871 contig_2885, whole genome shotgu	279	279	41%	6.00E-74	86.49	DPCP01000050.1
Query4	1B2N-R	Type I-B(2)	nucleatum (ATCC 25586)	Fusobacterium nucleatum isolate MGYG-HGUT-01464 genome assembly, contig	279	279	41%	6.00E-74	86.49	CABKTD010000001.1
Query4	1B2N-R	Type I-B(2)	nucleatum (ATCC 25586)	Fusobacterium nucleatum strain OH5060 scaffold_0, whole genome shotgun sequ	274	274	41%	3.00E-72	86.1	RQZD01000001.1
Query4	1B2N-R	Type I-B(2)	nucleatum (ATCC 25586)	Fusobacterium nucleatum subsp. polymorphum strain KCOM 1274 (=ChDC F309	274	274	41%	3.00E-72	86.1	NIRO01000001.1
Query4	1B2N-R	Type I-B(2)	nucleatum (ATCC 25586)	Fusobacterium nucleatum subsp. polymorphum strain KCOM 1257 (=ChDC F186	268	268	41%	1.00E-70	85.71	NIRL01000001.1
Query4	1B2N-R	Type I-B(2)	nucleatum (ATCC 25586)	Fusobacterium sp. HMSC064B11 Fusobacterium_spHMPREF3051-1.0_Cont666.6	268	268	41%	1.00E-70	85.71	LTSL01000155.1
Query4	1B2N-R	Type I-B(2)	nucleatum (ATCC 25586)	Fusobacterium nucleatum subsp. polymorphum strain KCOM 1232 (= ChDC F37)	252	252	40%	1.00E-65	85.26	NJGI01000001.1
Query4	1B2N-R	Type I-B(2)	nucleatum (ATCC 25586)	Fusobacterium nucleatum subsp. polymorphum strain KCOM 1267(=ChDC F290)	246	246	41%	6.00E-64	84.17	NIRM01000005.1
Query4	1B2N-R	Type I-B(2)	nucleatum (ATCC 25586)	Fusobacterium nucleatum subsp. polymorphum strain KCOM 1002 (=ChDC F175	244	244	41%	2.00E-63	83.91	NIRJ01000001.1
Query4	1B2N-R	Type I-B(2)	nucleatum (ATCC 25586)	Fusobacterium nucleatum subsp. animalis strain P2_CP NODE_1_length_135687	211	211	41%	2.00E-53	81.82	NPND01000001.1
Query4	1B2N-R	Type I-B(2)	nucleatum (ATCC 25586)	Fusobacterium nucleatum subsp. animalis strain P2_LM NODE_33_length_2780	211	211	41%	2.00E-53	81.82	NPNC01000033.1
Query4	1B2N-R	Type I-B(2)	nucleatum (ATCC 25586)	Fusobacterium nucleatum subsp. animalis strain KCOM 1280 (= ChDC F318) F31	206	206	41%	1.00E-51	81.47	NJGJ01000001.1
Query4	1B2N-R	Type I-B(2)	nucleatum (ATCC 25586)	Fusobacterium sp. HMSC065F01 Fusobacterium_spHMPREF2931-1.0_Cont493.1	206	206	41%	1.00E-51	81.23	LTWB01000069.1
Query4	1B2N-R	Type I-B(2)	nucleatum (ATCC 25586)	Fusobacterium nucleatum isolate MGYG-HGUT-01326 genome assembly, contig	206	206	41%	1.00E-51	81.47	CABKNP010000002.1
Query4	1B2N-R	Type I-B(2)	nucleatum (ATCC 25586)	Fusobacterium nucleatum CTI-1 acUTP-supercont1.3.C16, whole genome shotgu	206	206	41%	1.00E-51	81.47	AXNZ01000016.1
Query4	1B2N-R	Type I-B(2)	nucleatum (ATCC 25586)	Fusobacterium nucleatum CTI-3 acUTO-supercont1.6.C20, whole genome shotgu	206	206	41%	1.00E-51	81.4	AXNX01000020.1
Query4	1B2N-R	Type I-B(2)	nucleatum (ATCC 25586)	Fusobacterium nucleatum CTI-5 acUTW-supercont1.15.C21, whole genome shotg	206	206	41%	1.00E-51	81.3	AXNW01000021.1
Query4	1B2N-R	Type I-B(2)	nucleatum (ATCC 25586)	Fusobacterium nucleatum subsp. animalis ChDC F324 Contig101, whole genome	206	206	41%	1.00E-51	81.47	ATKD01000101.1
Query4	1B2N-R	Type I-B(2)	nucleatum (ATCC 25586)	Fusobacterium nucleatum subsp. animalis F0419 cont1.1, whole genome shotgun	206	206	41%	1.00E-51	81.23	AKCE01000001.1
Query4	1B2N-R	Type I-B(2)	nucleatum (ATCC 25586)	Fusobacterium nucleatum subsp. animalis 7_1 cont1.1, whole genome shotgun se	206	206	41%	1.00E-51	81.3	AKBT01000001.1
Query4	1B2N-R	Type I-B(2)	nucleatum (ATCC 25586)	Fusobacterium nucleatum subsp. animalis F0419 strain OT 420 cont1.6, whole ge	206	206	41%	1.00E-51	81.4	AGEH01000006.1
Query4	1B2N-R	Type I-B(2)	nucleatum (ATCC 25586)	Fusobacterium nucleatum subsp. animalis 21_1A cont3.2, whole genome shotgun	206	206	41%	1.00E-51	81.4	ADEE02000002.1
Query4	1B2N-R	Type I-B(2)	nucleatum (ATCC 25586)	Fusobacterium nucleatum subsp. polymorphum F0401 supercont2.1, whole genor	206	206	41%	1.00E-51	81.47	ADDB02000001.1
Query4	1B2N-R	Type I-B(2)	nucleatum (ATCC 25586)	Fusobacterium nucleatum subsp. animalis 11_3_2 cont1.7, whole genome shotgur	206	206	41%	1.00E-51	81.4	ACUO01000007.1
Query4	1B2N-R	Type I-B(2)	nucleatum (ATCC 25586)	Fusobacterium nucleatum subsp. animalis 3_1_33 cont2.3, whole genome shotgur	206	206	41%	1.00E-51	81.4	ACQE02000003.1
Query4	1B2N-R	Type I-B(2)	nucleatum (ATCC 25586)	Fusobacterium nucleatum subsp. animalis D11 adfWA-supercont2.5.C79, whole g	206	206	41%	1.00E-51	81.3	ACDS02000079.1
Query4	1B2N-R	Type I-B(2)	nucleatum (ATCC 25586)	Fusobacterium sp. CAG:649 WGS project CBBC01000000 data, contig, whole gen	200	200	41%	5.00E-50	81.08	CBBC010000190.1
Query4	1B2N-R	Type I-B(2)	nucleatum (ATCC 25586)	Fusobacterium nucleatum strain UMB0249 .16933_8_42.3, whole genome shotgur	195	195	41%	2.00E-48	80.69	PNHC01000003.1
Query4	1B2N-R	Type I-B(2)	nucleatum (ATCC 25586)	Fusobacterium nucleatum strain MJR7757B Fusobacterium_spHMPREF3221-1.0	195	195	41%	2.00E-48	80.69	LRPY01000169.1
Query4	1B2N-R	Type I-B(2)	nucleatum (ATCC 25586)	Fusobacterium sp. CM1 ctg7180000008867, whole genome shotgun sequence	189	189	41%	1.00E-46	80.15	JANB01000090.1
Query4	1B2N-R	Type I-B(2)	nucleatum (ATCC 25586)	Fusobacterium nucleatum subsp. animalis ATCC 51191 contig00129, whole genor	172	172	28%	1.00E-41	84.44	AFQD01000129.1

Supplementary Table S5. The validation of PCR amplification by each primer set (Continued)

Query	Sample_Name	CRISPR subtype	Subsp. (Type strain #)	Description	Max Score	Total Score	Query Cover	E value	Per. ident	Accession
Query5	1B2P-F	Type I-B(2)	polymorphum (ATCC 10953)	Fusobacterium nucleatum subsp. polymorphum ATCC 10953 Ctg92, whole genom	1146	5247	100%	0	100	AARG01000014.1
Query5	1B2P-F	Type I-B(2)	polymorphum (ATCC 10953)	Fusobacterium nucleatum 13_3C adlCx-supercont1.1.C11, whole genome shotgun	518	6841	100%	1.00E-145	83.59	JAOZ01000011.1
Query5	1B2P-F	Type I-B(2)	polymorphum (ATCC 10953)	Fusobacterium nucleatum isolate MGYG-HGUT-01464 genome assembly, contig:	518	6841	100%	1.00E-145	83.59	CABKTD010000001.1
Query5	1B2P-F	Type I-B(2)	polymorphum (ATCC 10953)	Fusobacterium nucleatum subsp. polymorphum strain KCOM 1257 (=ChDC F186	496	8933	100%	5.00E-139	84.93	NIRL01000001.1
Query5	1B2P-F	Type I-B(2)	polymorphum (ATCC 10953)	Fusobacterium sp. OBRC1 ctgN370L120013C40.9385, whole genome shotgun sequ	490	10394	100%	3.00E-137	82.65	JANA01000075.1
Query5	1B2P-F	Type I-B(2)	polymorphum (ATCC 10953)	Fusobacterium nucleatum subsp. polymorphum strain KCOM 1267(=ChDC F290)	484	3602	100%	1.00E-135	82.71	NIRM01000005.1
Query5	1B2P-F	Type I-B(2)	polymorphum (ATCC 10953)	Fusobacterium nucleatum subsp. polymorphum strain KCOM 1274 (=ChDC F309	481	5304	100%	2.00E-134	82.48	NIRO01000001.1
Query5	1B2P-F	Type I-B(2)	polymorphum (ATCC 10953)	Fusobacterium nucleatum strain 12230 NODE_1_length_386863_cov_114.933, wh	470	2419	100%	3.00E-131	83.98	QKOC01000001.1
Query5	1B2P-F	Type I-B(2)	polymorphum (ATCC 10953)	Fusobacterium nucleatum strain 12230 MIT 2016 NODE_17_length_36103_cov_2	470	2419	100%	3.00E-131	83.98	NXMD01000017.1
Query5	1B2P-F	Type I-B(2)	polymorphum (ATCC 10953)	TPA_asm: Fusobacterium sp. isolate UBA8871 contig_2885, whole genome shotg	470	2475	100%	3.00E-131	83.98	DPCP01000050.1
Query5	1B2P-F	Type I-B(2)	polymorphum (ATCC 10953)	Fusobacterium nucleatum subsp. polymorphum strain KCOM 1265 contig1, whol	466	4797	94%	4.00E-130	82.14	PEQW01000001.1
Query5	1B2P-F	Type I-B(2)	polymorphum (ATCC 10953)	Fusobacterium nucleatum subsp. polymorphum strain KCOM 1248 (=ChDC F113	466	4799	100%	4.00E-130	82.14	NIRK01000001.1
Query5	1B2P-F	Type I-B(2)	polymorphum (ATCC 10953)	Fusobacterium nucleatum CTI-6 addSJ-supercont1.12, whole genome shotgun se	448	2901	100%	2.00E-124	81.66	AXNV01000012.1
Query5	1B2P-F	Type I-B(2)	polymorphum (ATCC 10953)	Fusobacterium nucleatum strain OH5060 scaffold_0, whole genome shotgun sequ	427	9034	100%	2.00E-118	82.63	RQZD01000001.1
Query5	1B2P-F	Type I-B(2)	polymorphum (ATCC 10953)	Fusobacterium sp. HMSC064B11 Fusobacterium_spHMPREF3051-1.0_Cont666.2	422	970	91%	9.00E-117	92.08	LTSL01000151.1
Query5	1B2P-F	Type I-B(2)	polymorphum (ATCC 10953)	Fusobacterium sp. CM22 ctg7180000007352, whole genome shotgun sequence	407	913	80%	3.00E-112	97.87	JAQC01000070.1
Query5	1B2P-F	Type I-B(2)	polymorphum (ATCC 10953)	Fusobacterium nucleatum subsp. nucleatum ATCC 23726 contig00035, whole gen	396	4149	93%	6.00E-109	79.93	ADVK01000030.1
Query5	1B2P-F	Type I-B(2)	polymorphum (ATCC 10953)	Fusobacterium nucleatum subsp. nucleatum ChDC F316 Contig049, whole genom	381	1394	92%	2.00E-104	79.53	ATKC01000049.1
Query5	1B2P-F	Type I-B(2)	polymorphum (ATCC 10953)	Fusobacterium nucleatum subsp. nucleatum strain ChDC F311 F311_contig00000	377	8505	97%	2.00E-103	79.26	LMVH01000001.1
Query5	1B2P-F	Type I-B(2)	polymorphum (ATCC 10953)	Fusobacterium sp. oral taxon 370 str. F0437 F_sporaltaxon370F0437-1.0_Cont284	377	1168	92%	2.00E-103	79.32	AGAD01000088.1
Query5	1B2P-F	Type I-B(2)	polymorphum (ATCC 10953)	Fusobacterium hwasookii ChDC F128 contig00003, whole genome shotgun seque	363	7487	100%	6.00E-99	78.85	ALVD01000003.1
Query5	1B2P-F	Type I-B(2)	polymorphum (ATCC 10953)	Fusobacterium canifelinum strain OH4460_COT-188 scaffold_1, whole genome sh	340	3945	93%	3.00E-92	78.12	RQYY01000002.1
Query5	1B2P-F	Type I-B(2)	polymorphum (ATCC 10953)	Fusobacterium nucleatum subsp. polymorphum strain KCOM 1001 F119_1, whole	335	4100	92%	1.00E-90	81.43	NHRT01000001.1
Query5	1B2P-F	Type I-B(2)	polymorphum (ATCC 10953)	Fusobacterium hwasookii ChDC F300 Contig133, whole genome shotgun sequence	318	5917	100%	1.00E-85	78.89	ATKG01000133.1
Query5	1B2P-F	Type I-B(2)	polymorphum (ATCC 10953)	Fusobacterium hwasookii ChDC F145 Contig141, whole genome shotgun sequenc	318	5510	100%	1.00E-85	78.89	ATKE01000141.1
Query5	1B2P-F	Type I-B(2)	polymorphum (ATCC 10953)	Fusobacterium hwasookii ChDC F174 Contig035, whole genome shotgun sequenc	305	6721	93%	1.00E-81	90.25	ATKF01000035.1
Query5	1B2P-F	Type I-B(2)	polymorphum (ATCC 10953)	Fusobacterium nucleatum isolate MGYG-HGUT-01459 genome assembly, contig:	255	255	31%	1.00E-66	89.95	CABKSX010000028.1
Query5	1B2P-F	Type I-B(2)	polymorphum (ATCC 10953)	Fusobacterium nucleatum CTI-2 addTD-supercont1.34.C57, whole genome shotgu	255	255	31%	1.00E-66	89.95	AXNY01000057.1
Query5	1B2P-F	Type I-B(2)	polymorphum (ATCC 10953)	Fusobacterium sp. HMSC064B11 Fusobacterium_spHMPREF3051-1.0_Cont666.3	172	3489	67%	1.00E-41	78.14	LTSL01000152.1
Query5	1B2P-F	Type I-B(2)	polymorphum (ATCC 10953)	Fusobacterium sp. HMSC064B11 Fusobacterium_spHMPREF3051-1.0_Cont666.5	165	3292	67%	2.00E-39	75.17	LTSL01000154.1
Query5	1B2P-F	Type I-B(2)	polymorphum (ATCC 10953)	Fusobacterium sp. oral taxon 370 str. F0437 F_sporaltaxon370F0437-1.0_Cont132	158	1011	59%	3.00E-37	79.66	AGAD01000271.1
Query5	1B2P-F	Type I-B(2)	polymorphum (ATCC 10953)	Fusobacterium sp. HMSC064B11 Fusobacterium_spHMPREF3051-1.0_Cont666.6	148	1750	59%	2.00E-34	75	LTSL01000155.1
Query5	1B2P-F	Type I-B(2)	polymorphum (ATCC 10953)	Fusobacterium sp. oral taxon 370 str. F0437 F_sporaltaxon370F0437-1.0_Cont132	147	1344	67%	6.00E-34	76.62	AGAD01000270.1
Query5	1B2P-F	Type I-B(2)	polymorphum (ATCC 10953)	Fusobacterium hwasookii ChDC F174 Contig036, whole genome shotgun sequenc	141	1731	60%	3.00E-32	76.14	ATKF01000036.1
Query5	1B2P-F	Type I-B(2)	polymorphum (ATCC 10953)	Fusobacterium sp. oral taxon 370 str. F0437 F_sporaltaxon370F0437-1.0_Cont132	139	1613	61%	1.00E-31	76.05	AGAD01000269.1
Query5	1B2P-F	Type I-B(2)	polymorphum (ATCC 10953)	Fusobacterium nucleatum subsp. nucleatum ChDC F316 Contig050, whole genom	137	1634	59%	4.00E-31	74.48	ATKC01000050.1
Query5	1B2P-F	Type I-B(2)	polymorphum (ATCC 10953)	Fusobacterium sp. HMSC064B11 Fusobacterium_spHMPREF3051-1.0_Cont666.4	130	1612	60%	6.00E-29	77.55	LTSL01000153.1
Query5	1B2P-F	Type I-B(2)	polymorphum (ATCC 10953)	Fusobacterium sp. oral taxon 370 str. F0437 F_sporaltaxon370F0437-1.0_Cont132	128	1508	60%	2.00E-28	74.14	AGAD01000274.1
Query5	1B2P-F	Type I-B(2)	polymorphum (ATCC 10953)	Fusobacterium sp. oral taxon 370 str. F0437 F_sporaltaxon370F0437-1.0_Cont132	124	760	58%	3.00E-27	77.22	AGAD01000267.1
Query5	1B2P-F	Type I-B(2)	polymorphum (ATCC 10953)	Fusobacterium sp. oral taxon 370 str. F0437 F_sporaltaxon370F0437-1.0_Cont132	113	983	59%	6.00E-24	74.84	AGAD01000273.1
Query5	1B2P-F	Type I-B(2)	polymorphum (ATCC 10953)	Fusobacterium sp. oral taxon 370 str. F0437 F_sporaltaxon370F0437-1.0_Cont132	108	796	58%	3.00E-22	76.35	AGAD01000268.1
Query5	1B2P-F	Type I-B(2)	polymorphum (ATCC 10953)	Fusobacterium nucleatum subsp. polymorphum strain KCOM 1330 (=ChDC F330	102	838	58%	1.00E-20	78.74	NIRQ01000001.1
Query5	1B2P-F	Type I-B(2)	polymorphum (ATCC 10953)	Fusobacterium sp. CM22 ctg7180000007323, whole genome shotgun sequence	100	643	59%	5.00E-20	75.65	JAQC01000063.1
Query5	1B2P-F	Type I-B(2)	polymorphum (ATCC 10953)	Fusobacterium sp. oral taxon 370 str. F0437 F_sporaltaxon370F0437-1.0_Cont132	97.1	691	58%	6.00E-19	75.73	AGAD01000272.1
Query5	1B2P-F	Type I-B(2)	polymorphum (ATCC 10953)	Fusobacterium sp. CAG:649 WGS project CBBC01000000 data, contig, whole gen	84.2	84.2	9%	5.00E-15	93.22	CBBC010000033.1
Query5	1B2P-F	Type I-B(2)	polymorphum (ATCC 10953)	Fusobacterium sp. oral taxon 370 str. F0437 F_sporaltaxon370F0437-1.0_Cont132	78.7	609	59%	2.00E-13	81.63	AGAD01000266.1
Query5	1B2P-F	Type I-B(2)	polymorphum (ATCC 10953)	Fusobacterium nucleatum strain MJR7757B Fusobacterium_spHMPREF3221-1.0	71.3	135	12%	4.00E-11	100	LRPY01000169.1
Query5	1B2P-F	Type I-B(2)	polymorphum (ATCC 10953)	Fusobacterium sp. Marseille-P2749 strain Marseille-P2749T genome assembly, co	71.3	135	12%	4.00E-11	100	FMJA01000005.1
Query5	1B2P-F	Type I-B(2)	polymorphum (ATCC 10953)	Fusobacterium massiliense isolate MGYG-HGUT-01555 genome assembly, contig	71.3	135	12%	4.00E-11	100	CABKWM010000004.1
Query5	1B2P-F	Type I-B(2)	polymorphum (ATCC 10953)	Fusobacterium hwasookii ChDC F300 Contig091, whole genome shotgun sequenc	71.3	71.3	6%	4.00E-11	100	ATKG01000091.1
Query5	1B2P-F	Type I-B(2)	polymorphum (ATCC 10953)	Fusobacterium nucleatum subsp. vincentii ATCC 49256 Contig0376, whole genon	71.3	199	18%	4.00E-11	100	AABF01000002.1
Query5	1B2P-F	Type I-B(2)	polymorphum (ATCC 10953)	Fusobacterium periodonticum isolate MGYG-HGUT-01350 genome assembly, cor	69.4	69.4	5%	1.00E-10	100	CABKPC010000022.1
Query5	1B2P-F	Type I-B(2)	polymorphum (ATCC 10953)	Fusobacterium periodonticum 1_1_41FAA cont1.41, whole genome shotgun seque	69.4	69.4	5%	1.00E-10	100	ADGG01000041.1
Query5	1B2P-F	Type I-B(2)	polymorphum (ATCC 10953)	Fusobacterium nucleatum subsp. polymorphum strain KCOM 1271 (=ChDC F305	63.9	63.9	7%	6.00E-09	90	NIRN01000001.1
Query5	1B2P-F	Type I-B(2)	polymorphum (ATCC 10953)	Fusobacterium nucleatum isolate MGYG-HGUT-01459 genome assembly, contig:	56.5	329	28%	1.00E-06	100	CABKSX010000027.1
Query5	1B2P-F	Type I-B(2)	polymorphum (ATCC 10953)	Fusobacterium nucleatum CTI-2 addTD-supercont1.33.C56, whole genome shotgu	56.5	329	28%	1.00E-06	100	AXNY01000056.1

Query	Sample_Name	CRISPR subtype	Subsp. (Type strain #)	Description	Max Score	Total Score	Query Cover	E value	Per. ident	Accession
Query6	1B2P-R	Type I-B(2)	polymorphum (ATCC 10953)	Fusobacterium nucleatum subsp. polymorphum ATCC 10953 Ctg92, whole genom	1181	3908	100%	0	100	AARG01000014.1
Query6	1B2P-R	Type I-B(2)	polymorphum (ATCC 10953)	Fusobacterium nucleatum strain 12230 NODE_1_length_386863_cov_114.933, wh	931	2387	100%	0	96.15	QKOC01000001.1
Query6	1B2P-R	Type I-B(2)	polymorphum (ATCC 10953)	Fusobacterium nucleatum strain 12230 MIT 2016 NODE_17_length_36103_cov_2	931	2387	100%	0	96.15	NXMD01000017.1
Query6	1B2P-R	Type I-B(2)	polymorphum (ATCC 10953)	TPA_asm: Fusobacterium sp. isolate UBA8871 contig_2885, whole genome shotg	931	2387	100%	0	96.15	DPCP01000050.1
Query6	1B2P-R	Type I-B(2)	polymorphum (ATCC 10953)	Fusobacterium nucleatum subsp. polymorphum strain KCOM 1330 (=ChDC F330	852	1137	100%	0	90.87	NIRQ01000001.1
Query6	1B2P-R	Type I-B(2)	polymorphum (ATCC 10953)	Fusobacterium nucleatum subsp. polymorphum strain KCOM 1001 F119_1, whole	841	3168	100%	0	90.73	NHRT01000001.1
Query6	1B2P-R	Type I-B(2)	polymorphum (ATCC 10953)	Fusobacterium sp. CM22 ctg7180000007323, whole genome shotgun sequence	839	1093	99%	0	90.62	JAQC01000063.1
Query6	1B2P-R	Type I-B(2)	polymorphum (ATCC 10953)	Fusobacterium sp. OBRC1 ctgN370L120013C40.9385, whole genome shotgun sequ	824	7508	100%	0	90.23	JANA01000075.1
Query6	1B2P-R	Type I-B(2)	polymorphum (ATCC 10953)	Fusobacterium nucleatum 13_3C adlCx-supercont1.1.C11, whole genome shotgun	822	5653	100%	0	92.89	JAOZ01000011.1
Query6	1B2P-R	Type I-B(2)	polymorphum (ATCC 10953)	Fusobacterium nucleatum isolate MGYG-HGUT-01464 genome assembly, contig:	822	5653	100%	0	92.89	CABKTD010000001.1
Query6	1B2P-R	Type I-B(2)	polymorphum (ATCC 10953)	Fusobacterium nucleatum subsp. polymorphum strain KCOM 1265 contig1, whol	809	3665	100%	0	89.74	PEQW01000001.1
Query6	1B2P-R	Type I-B(2)	polymorphum (ATCC 10953)	Fusobacterium nucleatum subsp. polymorphum strain KCOM 1248 (=ChDC F113	809	3665	100%	0	89.74	NIRK01000001.1
Query6	1B2P-R	Type I-B(2)	polymorphum (ATCC 10953)	Fusobacterium nucleatum CTI-6 addSJ-supercont1.12, whole genome shotgun se	802	2482	100%	0	92.23	AXNV01000012.1
Query6	1B2P-R	Type I-B(2)	polymorphum (ATCC 10953)	Fusobacterium sp. HMSC064B11 Fusobacterium_spHMPREF3051-1.0_Cont666.6	795	1926	100%	0	92.03	LTSL01000155.1
Query6	1B2P-R	Type I-B(2)	polymorphum (ATCC 10953)	Fusobacterium nucleatum subsp. polymorphum strain KCOM 1257 (=ChDC F186	784	6594	100%	0	89.08	NIRL01000001.1
Query6	1B2P-R	Type I-B(2)	polymorphum (ATCC 10953)	Fusobacterium nucleatum subsp. polymorphum strain KCOM 1267(=ChDC F290)	726	2926	100%	0	87.46	NIRM01000005.1
Query6	1B2P-R	Type I-B(2)	polymorphum (ATCC 10953)	Fusobacterium nucleatum subsp. polymorphum strain KCOM 1274 (=ChDC F309	676	3686	100%	0	86.7	NIRO01000001.1
Query6	1B2P-R	Type I-B(2)	polymorphum (ATCC 10953)	Fusobacterium nucleatum strain OH5060 scaffold_0, whole genome shotgun sequ	520	6079	95%	3.00E-146	98.31	RQZD01000001.1
Query6	1B2P-R	Type I-B(2)	polymorphum (ATCC 10953)	Fusobacterium nucleatum subsp. polymorphum strain KCOM 1278 (=ChDC F313	446	446	39%	6.00E-124	98.42	NIRP01000004.1
Query6	1B2P-R	Type I-B(2)	polymorphum (ATCC 10953)	Fusobacterium nucleatum subsp. polymorphum strain KCOM 1271 (=ChDC F305	446	446	39%	6.00E-124	98.42	NIRN01000001.1
Query6	1B2P-R	Type I-B(2)	polymorphum (ATCC 10953)	Fusobacterium nucleatum subsp. polymorphum strain KCOM 1232 (= ChDC F37)	414	414	38%	2.00E-114	97.15	NJGI01000001.1
Query6	1B2P-R	Type I-B(2)	polymorphum (ATCC 10953)	Fusobacterium nucleatum subsp. polymorphum strain KCOM 1002 (=ChDC F175	344	344	38%	2.00E-93	91.6	NIRJ01000001.1
Query6	1B2P-R	Type I-B(2)	polymorphum (ATCC 10953)	Fusobacterium nucleatum subsp. nucleatum strain ChDC F311 F311_contig00000	283	5111	75%	5.00E-75	86.82	LMVH01000001.1
Query6	1B2P-R	Type I-B(2)	polymorphum (ATCC 10953)	Fusobacterium nucleatum isolate MGYG-HGUT-01459 genome assembly, contig:	283	447	54%	5.00E-75	86.82	CABKSX010000027.1
Query6	1B2P-R	Type I-B(2)	polymorphum (ATCC 10953)	Fusobacterium nucleatum CTI-2 addTD-supercont1.33.C56, whole genome shotgu	283	447	54%	5.00E-75	86.82	AXNY01000056.1
Query6	1B2P-R	Type I-B(2)	polymorphum (ATCC 10953)	Fusobacterium nucleatum subsp. nucleatum ChDC F316 Contig050, whole genom	283	1379	75%	5.00E-75	86.82	ATKC01000050.1
Query6	1B2P-R	Type I-B(2)	polymorphum (ATCC 10953)	Fusobacterium nucleatum subsp. nucleatum ATCC 23726 contig00035, whole gen	283	2748	77%	5.00E-75	86.82	ADVK01000030.1
Query6	1B2P-R	Type I-B(2)	polymorphum (ATCC 10953)	Fusobacterium canifelinum strain OH4460_COT-188 scaffold_1, whole genome sh	278	2621	96%	2.00E-73	87.04	RQYY01000002.1
Query6	1B2P-R	Type I-B(2)	polymorphum (ATCC 10953)	Fusobacterium hwasookii ChDC F300 Contig133, whole genome shotgun sequenc	265	4361	78%	2.00E-69	85.38	ATKG01000133.1
Query6	1B2P-R	Type I-B(2)	polymorphum (ATCC 10953)	Fusobacterium hwasookii ChDC F145 Contig141, whole genome shotgun sequenc	265	4079	77%	2.00E-69	85.6	ATKE01000141.1
Query6	1B2P-R	Type I-B(2)	polymorphum (ATCC 10953)	Fusobacterium hwasookii ChDC F128 contig00003, whole genome shotgun seque	265	5163	76%	2.00E-69	85.49	ALVD01000003.1
Query6	1B2P-R	Type I-B(2)	polymorphum (ATCC 10953)	Fusobacterium hwasookii ChDC F206 Contig012, whole genome shotgun sequenc	259	259	38%	8.00E-68	85.77	ATKH01000012.1
Query6	1B2P-R	Type I-B(2)	polymorphum (ATCC 10953)	Fusobacterium hwasookii ChDC F174 Contig036, whole genome shotgun sequenc	259	1432	75%	8.00E-68	84.88	ATKF01000036.1
Query6	1B2P-R	Type I-B(2)	polymorphum (ATCC 10953)	Fusobacterium nucleatum subsp. animalis strain P2_CP NODE_1_length_135687	252	252	39%	1.00E-65	84.65	NPND01000001.1
Query6	1B2P-R	Type I-B(2)	polymorphum (ATCC 10953)	Fusobacterium nucleatum subsp. animalis strain P2_LM NODE_33_length_27802	252	252	39%	1.00E-65	84.65	NPNC01000033.1
Query6	1B2P-R	Type I-B(2)	polymorphum (ATCC 10953)	Fusobacterium sp. HMSC064B11 Fusobacterium_spHMPREF3051-1.0_Cont666.3	154	2644	35%	4.00E-36	84.3	LTSL01000152.1
Query6	1B2P-R	Type I-B(2)	polymorphum (ATCC 10953)	Fusobacterium hwasookii ChDC F174 Contig035, whole genome shotgun sequenc	122	4668	35%	1.00E-26	77.54	ATKF01000035.1
Query6	1B2P-R	Type I-B(2)	polymorphum (ATCC 10953)	Fusobacterium sp. HMSC064B11 Fusobacterium_spHMPREF3051-1.0_Cont666.5	108	2271	38%	3.00E-22	76.6	LTSL01000154.1
Query6	1B2P-R	Type I-B(2)	polymorphum (ATCC 10953)	Fusobacterium sp. oral taxon 370 str. F0437 F_sporaltaxon370F0437-1.0_Cont132	108	1071	35%	3.00E-22	76.39	AGAD01000274.1
Query6	1B2P-R	Type I-B(2)	polymorphum (ATCC 10953)	Fusobacterium sp. oral taxon 370 str. F0437 F_sporaltaxon370F0437-1.0_Cont132	99	566	35%	2.00E-19	85.29	AGAD01000273.1
Query6	1B2P-R	Type I-B(2)	polymorphum (ATCC 10953)	Fusobacterium sp. oral taxon 370 str. F0437 F_sporaltaxon370F0437-1.0_Cont132	99	904	35%	2.00E-19	76.15	AGAD01000269.1
Query6	1B2P-R	Type I-B(2)	polymorphum (ATCC 10953)	Fusobacterium sp. oral taxon 370 str. F0437 F_sporaltaxon370F0437-1.0_Cont132	99	435	35%	2.00E-19	78.92	AGAD01000268.1
Query6	1B2P-R	Type I-B(2)	polymorphum (ATCC 10953)	Fusobacterium sp. oral taxon 370 str. F0437 F_sporaltaxon370F0437-1.0_Cont132	97.1	592	37%	7.00E-19	77.84	AGAD01000267.1
Query6	1B2P-R	Type I-B(2)	polymorphum (ATCC 10953)	Fusobacterium sp. HMSC064B11 Fusobacterium_spHMPREF3051-1.0_Cont666.4	95.3	948	37%	2.00E-18	75.73	LTSL01000153.1
Query6	1B2P-R	Type I-B(2)	polymorphum (ATCC 10953)	Fusobacterium sp. oral taxon 370 str. F0437 F_sporaltaxon370F0437-1.0_Cont132	93.5	1013	35%	9.00E-18	77.71	AGAD01000270.1
Query6	1B2P-R	Type I-B(2)	polymorphum (ATCC 10953)	Fusobacterium sp. oral taxon 370 str. F0437 F_sporaltaxon370F0437-1.0_Cont132	89.8	602	35%	1.00E-16	75.21	AGAD01000271.1
Query6	1B2P-R	Type I-B(2)	polymorphum (ATCC 10953)	Fusobacterium sp. oral taxon 370 str. F0437 F_sporaltaxon370F0437-1.0_Cont284	87.9	575	34%	4.00E-16	77.51	AGAD01000088.1
Query6	1B2P-R	Type I-B(2)	polymorphum (ATCC 10953)	Fusobacterium sp. oral taxon 370 str. F0437 F_sporaltaxon370F0437-1.0_Cont132	84.2	398	35%	5.00E-15	83.33	AGAD01000272.1
Query6	1B2P-R	Type I-B(2)	polymorphum (ATCC 10953)	Fusobacterium sp. oral taxon 370 str. F0437 F_sporaltaxon370F0437-1.0_Cont132	84.2	347	35%	5.00E-15	83	AGAD01000266.1
Query6	1B2P-R	Type I-B(2)	polymorphum (ATCC 10953)	Fusobacterium sp. HMSC064B11 Fusobacterium_spHMPREF3051-1.0_Cont666.2	78.7	376	29%	2.00E-13	81.82	LTSL01000151.1
Query6	1B2P-R	Type I-B(2)	polymorphum (ATCC 10953)	Fusobacterium sp. CM22 ctg7180000007352, whole genome shotgun sequence	78.7	304	30%	2.00E-13	81.82	JAQC01000070.1
Query6	1B2P-R	Type I-B(2)	polymorphum (ATCC 10953)	Fusobacterium nucleatum subsp. nucleatum ChDC F316 Contig049, whole genom	78.7	754	35%	2.00E-13	76.02	ATKC01000049.1
Query6	1B2P-R	Type I-B(2)	polymorphum (ATCC 10953)	Fusobacterium sp. CAG:649 WGS project CBBC01000000 data, contig, whole gen	67.6	67.6	6%	5.00E-10	97.44	CBBC010000033.1
Query6	1B2P-R	Type I-B(2)	polymorphum (ATCC 10953)	Fusobacterium nucleatum 13-08-02 DNA, NODE_27, whole genome shotgun sequ	67.6	67.6	5%	5.00E-10	100	BHYR01000025.1

Supplementary Table S5. The validation of PCR amplification by each primer set (Continued)

Query	Sample_Name	CRISPR subtype	Subsp. (Type strain #)	Description	Max Score	Total Score	Query Cover	E value	Per. ident	Accession
Query7	2A-F	Type II-A	vincentii (ATCC 49256)	Fusobacterium nucleatum subsp. vincentii strain KCOM 2880 contig1, whole gen	1144	1144	100%	0	100	PEIJ01000001.1
Query7	2A-F	Type II-A	vincentii (ATCC 49256)	Fusobacterium nucleatum subsp. vincentii ChDC F8 Contig132, whole genome sh	1144	1144	100%	0	100	ATKB01000132.1
Query7	2A-F	Type II-A	vincentii (ATCC 49256)	Fusobacterium nucleatum subsp. vincentii ATCC 49256 Contig0362, whole genom	1144	1144	100%	0	100	AABF01000014.1
Query7	2A-F	Type II-A	vincentii (ATCC 49256)	Fusobacterium sp. HMSC064B12 Fusobacterium_spHMPREF2775-1.0_Cont168.2	1050	1050	100%	0	97.25	LTGW01000035.1
Query7	2A-F	Type II-A	vincentii (ATCC 49256)	Fusobacterium nucleatum strain MJR7757B Fusobacterium_spHMPREF3221-1.0	1024	1024	99%	0	96.6	LRPY01000286.1
Query7	2A-F	Type II-A	vincentii (ATCC 49256)	Fusobacterium sp. CAG:649 WGS project CBBC01000000 data, contig, whole gen	1024	1024	99%	0	96.6	CBBC010000067.1
Query7	2A-F	Type II-A	vincentii (ATCC 49256)	Fusobacterium nucleatum subsp. nucleatum strain ChDC F311 F311_contig00000	974	974	99%	0	95.14	LMVH01000001.1
Query7	2A-F	Type II-A	vincentii (ATCC 49256)	Fusobacterium periodonticum isolate MGYG-HGUT-01350 genome assembly, co	649	649	99%	0	85.71	CABKPC010000024.1
Query7	2A-F	Type II-A	vincentii (ATCC 49256)	Fusobacterium periodonticum 1_1_41FAA cont1.21, whole genome shotgun seque	649	649	99%	0	85.71	ADGG01000021.1
Query	Sample_Name	CRISPR subtype	Subsp. (Type strain #)	Description	Max Score	Total Score	Query Cover	E value	Per. ident	Accession
Query8	2A-R	Type II-A	vincentii (ATCC 49256)	Fusobacterium nucleatum subsp. vincentii strain NCTC11326 genome assembly,	1146	1146	100%	0	100	UGGX01000003.1
Query8	2A-R	Type II-A	vincentii (ATCC 49256)	Fusobacterium nucleatum strain AB1 PROKKA_contig000004, whole genome sho	1146	1146	100%	0	100	MLQO01000004.1
Query8	2A-R	Type II-A	vincentii (ATCC 49256)	Fusobacterium sp. CM21 contig00019, whole genome shotgun sequence	1146	1146	100%	0	100	AZYU01000404.1
Query8	2A-R	Type II-A	vincentii (ATCC 49256)	Fusobacterium nucleatum CTI-7 acUUj-supercont1.25.C38, whole genome shotgu	1146	1146	100%	0	100	AXNU01000038.1
Query8	2A-R	Type II-A	vincentii (ATCC 49256)	Fusobacterium nucleatum subsp. vincentii ATCC 51190 Contig00002, whole geno	1146	1146	100%	0	100	AKXI01000002.1
Query8	2A-R	Type II-A	vincentii (ATCC 49256)	Fusobacterium nucleatum subsp. vincentii ATCC 49256 Contig0326, whole genom	1146	1146	100%	0	100	AABF01000064.1
Query8	2A-R	Type II-A	vincentii (ATCC 49256)	Fusobacterium nucleatum subsp. vincentii 4_1_13 cont2.13, whole genome shotgu	1134	1134	100%	0	99.68	ACDE02000013.1
Query8	2A-R	Type II-A	vincentii (ATCC 49256)	Fusobacterium nucleatum subsp. vincentii strain KCOM 2880 contig1, whole gen	1118	1118	100%	0	99.19	PEIJ01000001.1
Query8	2A-R	Type II-A	vincentii (ATCC 49256)	Fusobacterium nucleatum subsp. vincentii ChDC F8 Contig130, whole genome sh	1118	1118	100%	0	99.19	ATKB01000130.1
Query8	2A-R	Type II-A	vincentii (ATCC 49256)	Fusobacterium sp. HMSC064B12 Fusobacterium_spHMPREF2775-1.0_Cont168.2	1096	1096	100%	0	98.55	LTGW01000031.1
Query8	2A-R	Type II-A	vincentii (ATCC 49256)	Fusobacterium nucleatum CTI-5 acUTW-supercont1.13.C19, whole genome shotg	778	881	92%	0	94.81	AXNW01000019.1
Query8	2A-R	Type II-A	vincentii (ATCC 49256)	Fusobacterium nucleatum subsp. animalis strain KCOM 1280 (= ChDC F318) F31	774	774	80%	0	94.62	NJGJ01000001.1
Query8	2A-R	Type II-A	vincentii (ATCC 49256)	Fusobacterium nucleatum subsp. animalis strain P2_CP NODE_19_length_44291	773	773	80%	0	94.61	NPND01000019.1
Query8	2A-R	Type II-A	vincentii (ATCC 49256)	Fusobacterium nucleatum subsp. animalis strain P2_LM NODE_23_length_3733	773	773	80%	0	94.61	NPNC01000023.1
Query8	2A-R	Type II-A	vincentii (ATCC 49256)	Fusobacterium sp. CM1 ctg7180000008793, whole genome shotgun sequence	773	884	92%	0	94.61	JANB01000112.1
Query8	2A-R	Type II-A	vincentii (ATCC 49256)	Fusobacterium nucleatum subsp. animalis 21_1A cont3.2, whole genome shotgun	773	884	92%	0	94.61	ADEE02000002.1
Query8	2A-R	Type II-A	vincentii (ATCC 49256)	Fusobacterium nucleatum strain UMB0249 .16933_8_42.1, whole genome shotgu	767	767	80%	0	94.41	PNHC01000001.1
Query8	2A-R	Type II-A	vincentii (ATCC 49256)	Fusobacterium sp. HMSC065F01 Fusobacterium_spHMPREF2931-1.0_Cont493.2	767	767	80%	0	94.41	LTWB01000077.1
Query8	2A-R	Type II-A	vincentii (ATCC 49256)	Fusobacterium nucleatum subsp. animalis ChDC F324 Contig069, whole genome	767	767	80%	0	94.41	ATKD01000069.1
Query8	2A-R	Type II-A	vincentii (ATCC 49256)	Fusobacterium nucleatum subsp. animalis 11_3_2 cont1.10, whole genome shotgu	767	870	92%	0	94.41	ACUO01000010.1
Query8	2A-R	Type II-A	vincentii (ATCC 49256)	Fusobacterium nucleatum subsp. animalis 3_1_33 cont2.6, whole genome shotgu	767	870	92%	0	94.58	ACQE02000006.1
Query8	2A-R	Type II-A	vincentii (ATCC 49256)	Fusobacterium nucleatum subsp. animalis D11 adfWA-supercont2.8.C117, whole	767	767	80%	0	94.58	ACDS02000117.1
Query8	2A-R	Type II-A	vincentii (ATCC 49256)	Fusobacterium sp. CAG:649 WGS project CBBC01000000 data, contig, whole gen	761	859	92%	0	94.21	CBBC010000067.1
Query8	2A-R	Type II-A	vincentii (ATCC 49256)	Fusobacterium nucleatum isolate MGYG-HGUT-01326 genome assembly, contig	761	761	80%	0	94.21	CABKNP010000002.1
Query8	2A-R	Type II-A	vincentii (ATCC 49256)	Fusobacterium nucleatum CTI-1 acUTP-supercont1.3.C21, whole genome shotgu	761	864	92%	0	94.38	AXNZ01000021.1
Query8	2A-R	Type II-A	vincentii (ATCC 49256)	Fusobacterium nucleatum subsp. animalis F0419 cont1.1, whole genome shotgun	761	761	80%	0	94.21	AKCE01000001.1
Query8	2A-R	Type II-A	vincentii (ATCC 49256)	Fusobacterium nucleatum subsp. animalis 7_1 cont1.1, whole genome shotgun se	761	761	80%	0	94.21	AKBT01000001.1
Query8	2A-R	Type II-A	vincentii (ATCC 49256)	Fusobacterium nucleatum subsp. animalis F0419 strain OT 420 cont1.9, whole ge	761	761	80%	0	94.38	AGEH01000009.1
Query8	2A-R	Type II-A	vincentii (ATCC 49256)	Fusobacterium nucleatum subsp. polymorphum F0401 supercont2.1, whole genom	761	761	80%	0	94.21	ADDB02000001.1
Query8	2A-R	Type II-A	vincentii (ATCC 49256)	Fusobacterium nucleatum strain MJR7757B Fusobacterium_spHMPREF3221-1.0	756	853	92%	0	94.01	LRPY01000288.1
Query8	2A-R	Type II-A	vincentii (ATCC 49256)	Fusobacterium nucleatum CC53 contig_seqid4, whole genome shotgun sequence	756	756	80%	0	94.18	AOUL01000004.1
Query8	2A-R	Type II-A	vincentii (ATCC 49256)	Fusobacterium nucleatum CTI-3 acUTO-supercont1.6.C20, whole genome shotgu	745	848	92%	0	93.61	AXNX01000020.1
Query8	2A-R	Type II-A	vincentii (ATCC 49256)	Fusobacterium nucleatum 13-08-02 DNA, NODE_35, whole genome shotgun sequ	741	741	77%	0	94.77	BHYR01000032.1
Query8	2A-R	Type II-A	vincentii (ATCC 49256)	Fusobacterium nucleatum W1481 Contig51, whole genome shotgun sequence	686	686	77%	0	92.69	AXUR01000051.1
Query8	2A-R	Type II-A	vincentii (ATCC 49256)	Fusobacterium nucleatum subsp. nucleatum ATCC 23726 contig00024, whole gen	686	686	77%	0	92.68	ADVK01000020.1
Query8	2A-R	Type II-A	vincentii (ATCC 49256)	Fusobacterium nucleatum subsp. animalis ATCC 51191 contig00271, whole genom	678	678	68%	0	95.53	AFQD01000271.1
Query8	2A-R	Type II-A	vincentii (ATCC 49256)	Fusobacterium nucleatum subsp. polymorphum strain KCOM 1330 (=ChDC F330	676	676	81%	0	90.94	NIRQ01000001.1
Query8	2A-R	Type II-A	vincentii (ATCC 49256)	Fusobacterium nucleatum subsp. polymorphum strain KCOM 1278 (=ChDC F313	676	676	81%	0	90.94	NIRP01000004.1
Query8	2A-R	Type II-A	vincentii (ATCC 49256)	Fusobacterium nucleatum subsp. polymorphum strain KCOM 1248 (=ChDC F113	676	676	81%	0	90.94	NIRK01000001.1
Query8	2A-R	Type II-A	vincentii (ATCC 49256)	Fusobacterium nucleatum subsp. nucleatum strain ChDC F311 F311_contig00000	675	675	77%	0	92.26	LMVH01000001.1
Query8	2A-R	Type II-A	vincentii (ATCC 49256)	Fusobacterium nucleatum subsp. nucleatum ChDC F316 Contig058, whole genom	675	675	77%	0	92.26	ATKC01000058.1
Query8	2A-R	Type II-A	vincentii (ATCC 49256)	Fusobacterium nucleatum subsp. polymorphum strain KCOM 1271 (=ChDC F305	671	671	80%	0	91	NIRN01000001.1
Query8	2A-R	Type II-A	vincentii (ATCC 49256)	Fusobacterium nucleatum subsp. polymorphum strain KCOM 1002 (=ChDC F175	671	671	80%	0	91	NIRJ01000001.1
Query8	2A-R	Type II-A	vincentii (ATCC 49256)	Fusobacterium nucleatum subsp. polymorphum strain KCOM 1267(=ChDC F290)	669	669	82%	0	90.31	NIRM01000002.1
Query8	2A-R	Type II-A	vincentii (ATCC 49256)	Fusobacterium nucleatum isolate MGYG-HGUT-01459 genome assembly, contig	669	669	77%	0	92.05	CABKSX010000021.1
Query8	2A-R	Type II-A	vincentii (ATCC 49256)	Fusobacterium nucleatum CTI-2 addTD-supercont1.28.C49, whole genome shotgu	669	669	77%	0	92.05	AXNY01000049.1
Query8	2A-R	Type II-A	vincentii (ATCC 49256)	Fusobacterium nucleatum strain 12230 NODE_12_length_77059_cov_105.367, wh	667	667	82%	0	90.43	QKOC01000012.1
Query8	2A-R	Type II-A	vincentii (ATCC 49256)	Fusobacterium nucleatum strain 12230 MIT 2016 NODE_20_length_35538_cov_1	667	667	82%	0	90.43	NXMD01000020.1
Query8	2A-R	Type II-A	vincentii (ATCC 49256)	Fusobacterium nucleatum 13_3C adlCx-supercont1.1.C7, whole genome shotgun	667	667	82%	0	90.43	JAOZ01000007.1
Query8	2A-R	Type II-A	vincentii (ATCC 49256)	TPA_asm: Fusobacterium sp. isolate UBA8871 contig_614, whole genome shotgu	667	667	82%	0	90.43	DPCP01000031.1
Query8	2A-R	Type II-A	vincentii (ATCC 49256)	Fusobacterium nucleatum isolate MGYG-HGUT-01464 genome assembly, contig	667	667	82%	0	90.43	CABKTD010000001.1
Query8	2A-R	Type II-A	vincentii (ATCC 49256)	Fusobacterium nucleatum CTI-6 addSJ-supercont1.16, whole genome shotgun se	667	667	79%	0	91.28	AXNV01000016.1
Query8	2A-R	Type II-A	vincentii (ATCC 49256)	Fusobacterium nucleatum subsp. polymorphum strain KCOM 1001 F119_1, whol	665	665	81%	0	90.55	NHRT01000001.1
Query8	2A-R	Type II-A	vincentii (ATCC 49256)	Fusobacterium sp. CM22 ctg7180000007327, whole genome shotgun sequence	665	665	80%	0	90.8	JAQC01000053.1
Query8	2A-R	Type II-A	vincentii (ATCC 49256)	Fusobacterium nucleatum subsp. polymorphum strain KCOM 1265 contig1, whol	664	664	82%	0	90.12	PEQW01000001.1
Query8	2A-R	Type II-A	vincentii (ATCC 49256)	Fusobacterium nucleatum subsp. polymorphum strain KCOM 1274 (=ChDC F309	664	664	82%	0	90.12	NIRO01000002.1
Query8	2A-R	Type II-A	vincentii (ATCC 49256)	Fusobacterium nucleatum subsp. polymorphum strain KCOM 1257 (=ChDC F186	664	664	82%	0	90.12	NIRL01000001.1
Query8	2A-R	Type II-A	vincentii (ATCC 49256)	Fusobacterium sp. OBRC1 ctgN311L199939C41.22, whole genome shotgun seque	664	664	82%	0	90.12	JANA01000084.1
Query8	2A-R	Type II-A	vincentii (ATCC 49256)	Fusobacterium nucleatum subsp. polymorphum ATCC 10953 Ctg91, whole genom	664	664	82%	0	90.12	AARG01000013.1
Query8	2A-R	Type II-A	vincentii (ATCC 49256)	Fusobacterium nucleatum strain OH5060 scaffold_2, whole genome shotgun sequ	656	656	79%	0	90.87	RQZD01000003.1
Query8	2A-R	Type II-A	vincentii (ATCC 49256)	Fusobacterium nucleatum subsp. polymorphum strain KCOM 1232 (= ChDC F37)	652	652	82%	0	89.73	NJGI01000001.1
Query8	2A-R	Type II-A	vincentii (ATCC 49256)	Fusobacterium sp. HMSC064B11 Fusobacterium_spHMPREF3051-1.0_Cont221.2	645	645	79%	0	90.47	LTSL01000033.1
Query8	2A-R	Type II-A	vincentii (ATCC 49256)	Fusobacterium canifelinum strain OH4460_COT-188 scaffold_0, whole genome sh	627	627	78%	2.00E-178	90.12	RQYY01000001.1
Query8	2A-R	Type II-A	vincentii (ATCC 49256)	Fusobacterium hwasookii ChDC F128 contig00001, whole genome shotgun seque	608	608	77%	7.00E-173	89.75	ALVD01000001.1
Query8	2A-R	Type II-A	vincentii (ATCC 49256)	Fusobacterium hwasookii ChDC F174 Contig017, whole genome shotgun sequenc	603	603	77%	3.00E-171	89.54	ATKF01000017.1
Query8	2A-R	Type II-A	vincentii (ATCC 49256)	Fusobacterium hwasookii ChDC F206 Contig019, whole genome shotgun sequenc	595	595	76%	5.00E-169	89.47	ATKH01000019.1
Query8	2A-R	Type II-A	vincentii (ATCC 49256)	Fusobacterium hwasookii ChDC F145 Contig129, whole genome shotgun sequenc	590	590	76%	2.00E-167	89.26	ATKE01000129.1
Query8	2A-R	Type II-A	vincentii (ATCC 49256)	Fusobacterium pseudoperiodonticum strain KCOM 1321 contig1, whole genome	525	525	68%	7.00E-148	89.1	PEQX01000001.1
Query8	2A-R	Type II-A	vincentii (ATCC 49256)	Fusobacterium pseudoperiodonticum strain KCOM 1259 contig1, whole genome	520	520	68%	3.00E-146	88.86	PEQY01000001.1
Query8	2A-R	Type II-A	vincentii (ATCC 49256)	Fusobacterium periodonticum isolate MGYG-HGUT-01350 genome assembly, co	520	520	68%	3.00E-146	88.86	CABKPC010000024.1
Query8	2A-R	Type II-A	vincentii (ATCC 49256)	Fusobacterium periodonticum isolate MGYG-HGUT-01324 genome assembly, co	520	520	67%	3.00E-146	89.18	CABKNO010000001.1
Query8	2A-R	Type II-A	vincentii (ATCC 49256)	Fusobacterium periodonticum 1_1_41FAA cont1.26, whole genome shotgun seque	520	520	68%	3.00E-146	88.86	ADGG01000026.1
Query8	2A-R	Type II-A	vincentii (ATCC 49256)	Fusobacterium periodonticum D10 cont1.234, whole genome shotgun sequence	520	520	67%	3.00E-146	89.18	ACIF01000234.1
Query8	2A-R	Type II-A	vincentii (ATCC 49256)	Fusobacterium periodonticum 2_1_31 acIQb-supercont3.1.C7, whole genome sho	520	520	67%	3.00E-146	89.18	ACDC03000007.1
Query8	2A-R	Type II-A	vincentii (ATCC 49256)	Fusobacterium hwasookii ChDC F300 Contig053, whole genome shotgun sequenc	512	512	62%	5.00E-144	90.49	ATKG01000053.1
Query8	2A-R	Type II-A	vincentii (ATCC 49256)	Fusobacterium periodonticum ATCC 33693 F_periodonticum-1.0.1_Cont25.7, wh	503	503	68%	3.00E-141	88.15	ACJY01000111.1
Query8	2A-R	Type II-A	vincentii (ATCC 49256)	Fusobacterium sp. Marseille-P2749 strain Marseille-P2749T genome assembly, c	399	399	71%	4.00E-110	83.04	FMJA01000007.1
Query8	2A-R	Type II-A	vincentii (ATCC 49256)	Fusobacterium massiliense isolate MGYG-HGUT-01555 genome assembly, contig	399	399	71%	4.00E-110	83.04	CABKWM010000006.1
Query8	2A-R	Type II-A	vincentii (ATCC 49256)	Fusobacterium sp. oral taxon 370 str. F0437 F_sporaltaxon370F0437-1.0_Cont26	392	392	53%	7.00E-108	88.18	AGAD01000082.1
Query8	2A-R	Type II-A	vincentii (ATCC 49256)	Fusobacterium periodonticum ATCC 33693 F_periodonticum-1.0.1_Cont25.20, w	102	102	11%	1.00E-20	92.96	ACJY01000099.1
Query8	2A-R	Type II-A	vincentii (ATCC 49256)	Fusobacterium sp. oral taxon 370 str. F0437 F_sporaltaxon370F0437-1.0_Cont25	97.1	97.1	11%	6.00E-19	91.55	AGAD01000081.1
Query8	2A-R	Type II-A	vincentii (ATCC 49256)	Fusobacterium gonidiaformans ATCC 25563 cont2.23, whole genome shotgun seq	84.2	84.2	20%	5.00E-15	78.95	ACET02000023.1
Query	Sample_Name	CRISPR subtype	Subsp. (Type strain #)	Description	Max Score	Total Score	Query Cover	E value	Per. ident	Accession
Query9	3A-F	Type III-A	animalis (ATCC 51191)	Fusobacterium nucleatum subsp. animalis ATCC 51191 contig00206, whole genom	1027	1027	100%	0	99.82	AFQD01000206.1
Query9	3A-F	Type III-A	animalis (ATCC 51191)	Fusobacterium nucleatum subsp. animalis strain P2_CP NODE_49_length_10001	824	824	100%	0	93.24	NPND01000049.1
Query9	3A-F	Type III-A	animalis (ATCC 51191)	Fusobacterium nucleatum subsp. animalis strain P2_LM NODE_63_length_1052	824	824	100%	0	93.24	NPNC01000063.1
Query9	3A-F	Type III-A	animalis (ATCC 51191)	Fusobacterium nucleatum CTI-1 acUTP-supercont1.3.C16, whole genome shotgu	785	785	100%	0	91.99	AXNZ01000016.1
Query9	3A-F	Type III-A	animalis (ATCC 51191)	Fusobacterium sp. CM1 ctg7180000008772, whole genome shotgun sequence	763	763	100%	0	91.3	JANB01000066.1
Query9	3A-F	Type III-A	animalis (ATCC 51191)	Fusobacterium nucleatum CTI-5 acUTW-supercont1.33.C45, whole genome shotg	760	760	94%	0	92.48	AXNW01000045.1
Query9	3A-F	Type III-A	animalis (ATCC 51191)	Fusobacterium nucleatum subsp. vincentii 4_1_13 cont2.13, whole genome shotgu	745	745	97%	0	91.24	ACDE02000013.1
Query9	3A-F	Type III-A	animalis (ATCC 51191)	Fusobacterium nucleatum W1481 Contig70, whole genome shotgun sequence	736	736	99%	0	89.29	AXUR01000070.1
Query9	3A-F	Type III-A	animalis (ATCC 51191)	Fusobacterium canifelinum strain OH4460_COT-188 scaffold_2, whole genome sh	732	732	100%	0	90.23	RQYY01000003.1
Query9	3A-F	Type III-A	animalis (ATCC 51191)	Fusobacterium nucleatum 13-08-02 DNA, NODE_1, whole genome shotgun seque	730	730	100%	0	90.2	BHYR01000001.1
Query9	3A-F	Type III-A	animalis (ATCC 51191)	Fusobacterium nucleatum strain 12230 NODE_2_length_191728_cov_154.899, wh	695	695	97%	0	89.62	QKOC01000002.1
Query9	3A-F	Type III-A	animalis (ATCC 51191)	Fusobacterium nucleatum strain 12230 MIT 2016 NODE_11_length_49682_cov_2	695	695	97%	0	89.62	NXMD01000011.1
Query9	3A-F	Type III-A	animalis (ATCC 51191)	Fusobacterium nucleatum 13_3C adlCx-supercont1.1.C4, whole genome shotgun	695	695	99%	0	89.13	JAOZ01000004.1
Query9	3A-F	Type III-A	animalis (ATCC 51191)	TPA_asm: Fusobacterium sp. isolate UBA8871 contig_961, whole genome shotgu	695	695	97%	0	89.62	DPCP01000032.1
Query9	3A-F	Type III-A	animalis (ATCC 51191)	Fusobacterium nucleatum isolate MGYG-HGUT-01464 genome assembly, contig	695	695	99%	0	89.13	CABKTD010000001.1
Query9	3A-F	Type III-A	animalis (ATCC 51191)	Fusobacterium nucleatum subsp. polymorphum strain KCOM 1001 F119_1, whol	693	693	100%	0	88.99	NHRT01000001.1
Query9	3A-F	Type III-A	animalis (ATCC 51191)	Fusobacterium hwasookii ChDC F174 Contig063, whole genome shotgun sequenc	693	693	100%	0	88.99	ATKF01000063.1
Query9	3A-F	Type III-A	animalis (ATCC 51191)	Fusobacterium nucleatum subsp. polymorphum strain KCOM 1267(=ChDC F290)	680	680	99%	0	88.61	NIRM01000002.1
Query9	3A-F	Type III-A	animalis (ATCC 51191)	Fusobacterium hwasookii ChDC F300 Contig043, whole genome shotgun sequenc	678	678	99%	0	88.59	ATKG01000043.1
Query9	3A-F	Type III-A	animalis (ATCC 51191)	Fusobacterium hwasookii ChDC F145 Contig047, whole genome shotgun sequenc	678	678	99%	0	88.59	ATKE01000047.1
Query9	3A-F	Type III-A	animalis (ATCC 51191)	Fusobacterium nucleatum subsp. polymorphum strain KCOM 1232 (= ChDC F37)	669	669	99%	0	88.26	NJGI01000001.1
Query9	3A-F	Type III-A	animalis (ATCC 51191)	Fusobacterium hwasookii ChDC F206 Contig065, whole genome shotgun sequenc	669	669	99%	0	88.26	ATKH01000065.1
Query9	3A-F	Type III-A	animalis (ATCC 51191)	Fusobacterium periodonticum isolate MGYG-HGUT-01324 genome assembly, co	483	483	71%	4.00E-135	88.47	CABKNO010000001.1
Query9	3A-F	Type III-A	animalis (ATCC 51191)	Fusobacterium periodonticum 2_1_31 acIQb-supercont3.1.C6, whole genome sho	483	483	71%	4.00E-135	88.47	ACDC03000006.1
Query9	3A-F	Type III-A	animalis (ATCC 51191)	Fusobacterium periodonticum isolate MGYG-HGUT-01350 genome assembly, co	472	472	71%	8.00E-132	87.97	CABKPC010000024.1
Query9	3A-F	Type III-A	animalis (ATCC 51191)	Fusobacterium periodonticum 1_1_41FAA cont1.29, whole genome shotgun seque	472	472	71%	8.00E-132	87.97	ADGG01000029.1
Query9	3A-F	Type III-A	animalis (ATCC 51191)	Fusobacterium pseudoperiodonticum strain KCOM 1321 contig1, whole genome	449	449	71%	4.00E-125	86.97	PEQX01000001.1

Supplementary Table S5. The validation of PCR amplification by each primer set (Continued)

Query	Sample_Name	CRISPR subtype	Subsp. (Type strain #)	Description	Max Score	Total Score	Query Cover	E value	Per. ident	Accession
Query10	3A-R	Type III-A	animalis (ATCC 51191)	Fusobacterium nucleatum subsp. animalis ATCC 51191 contig00205, whole genon	1033	1294	100%	0	99.47	AFQD01000205.1
Query10	3A-R	Type III-A	animalis (ATCC 51191)	Fusobacterium nucleatum CTI-5 acUTW-supercont1.23.C33, whole genome shotg	1026	1026	100%	0	99.13	AXNW01000033.1
Query10	3A-R	Type III-A	animalis (ATCC 51191)	Fusobacterium sp. CM1 ctg7180000008772, whole genome shotgun sequence	1024	1024	100%	0	99.12	JANB01000066.1
Query10	3A-R	Type III-A	animalis (ATCC 51191)	Fusobacterium nucleatum CTI-1 acUTP-supercont1.3.C16, whole genome shotgu	1014	1014	100%	0	98.77	AXNZ01000016.1
Query10	3A-R	Type III-A	animalis (ATCC 51191)	Fusobacterium nucleatum strain UMB0249 .16933_8_42.2, whole genome shotgu	549	549	56%	4.00E-155	97.52	PNHC01000002.1
Query10	3A-R	Type III-A	animalis (ATCC 51191)	Fusobacterium nucleatum subsp. animalis strain KCOM 1280 (= ChDC F318) F31	549	549	54%	4.00E-155	98.71	NJGJ01000001.1
Query10	3A-R	Type III-A	animalis (ATCC 51191)	Fusobacterium nucleatum subsp. animalis F0419 cont1.2, whole genome shotgun	549	549	56%	4.00E-155	97.52	AKCE01000002.1
Query10	3A-R	Type III-A	animalis (ATCC 51191)	Fusobacterium nucleatum subsp. animalis F0419 strain OT 420 cont1.36, whole g	549	549	56%	4.00E-155	97.52	AGEH01000036.1
Query10	3A-R	Type III-A	animalis (ATCC 51191)	Fusobacterium nucleatum subsp. animalis D11 adfWA-supercont2.2.C13, whole g	544	544	54%	2.00E-153	98.38	ACDS02000013.1
Query10	3A-R	Type III-A	animalis (ATCC 51191)	Fusobacterium nucleatum strain MJR7757B Fusobacterium_spHMPREF3221-1.0	538	538	56%	8.00E-152	96.62	LRPY01000058.1
Query10	3A-R	Type III-A	animalis (ATCC 51191)	Fusobacterium nucleatum isolate MGYG-HGUT-01326 genome assembly, contig:	538	538	56%	8.00E-152	96.89	CABKNP010000002.1
Query10	3A-R	Type III-A	animalis (ATCC 51191)	Fusobacterium nucleatum subsp. animalis 7_1 cont1.1, whole genome shotgun se	538	538	56%	8.00E-152	96.89	AKBT01000001.1
Query10	3A-R	Type III-A	animalis (ATCC 51191)	Fusobacterium nucleatum subsp. polymorphum F0401 supercont2.1, whole genor	538	538	56%	8.00E-152	96.89	ADDB02000001.1
Query10	3A-R	Type III-A	animalis (ATCC 51191)	Fusobacterium nucleatum subsp. animalis 11_3_2 cont1.3, whole genome shotgun	538	538	56%	8.00E-152	96.89	ACUO01000003.1
Query10	3A-R	Type III-A	animalis (ATCC 51191)	Fusobacterium nucleatum subsp. animalis 3_1_33 cont2.3, whole genome shotgun	538	538	56%	8.00E-152	96.89	ACQE02000003.1
Query10	3A-R	Type III-A	animalis (ATCC 51191)	Fusobacterium sp. HMSC065F01 Fusobacterium_spHMPREF2931-1.0_Cont493.2	532	532	54%	4.00E-150	97.73	LTWB01000080.1
Query10	3A-R	Type III-A	animalis (ATCC 51191)	Fusobacterium sp. CAG:649 WGS project CBBC01000000 data, contig, whole gen	532	532	56%	4.00E-150	96.58	CBBC010000054.1
Query10	3A-R	Type III-A	animalis (ATCC 51191)	Fusobacterium nucleatum CTI-3 acUTO-supercont1.6.C20, whole genome shotgu	532	532	54%	4.00E-150	97.73	AXNX01000020.1
Query10	3A-R	Type III-A	animalis (ATCC 51191)	Fusobacterium nucleatum subsp. animalis 21_1A cont3.2, whole genome shotgun	532	532	54%	4.00E-150	97.73	ADEE02000002.1
Query10	3A-R	Type III-A	animalis (ATCC 51191)	Fusobacterium nucleatum subsp. vincentii strain NCTC11326 genome assembly,	505	505	55%	8.00E-142	95.31	UGGX01000003.1
Query10	3A-R	Type III-A	animalis (ATCC 51191)	Fusobacterium nucleatum strain AB1 PROKKA_contig000036, whole genome sho	505	505	56%	8.00E-142	95.05	MLQO01000036.1
Query10	3A-R	Type III-A	animalis (ATCC 51191)	Fusobacterium sp. CM21 contig00092, whole genome shotgun sequence	505	505	55%	8.00E-142	95.31	AZYU01000374.1
Query10	3A-R	Type III-A	animalis (ATCC 51191)	Fusobacterium nucleatum CTI-7 acUUj-supercont1.24.C37, whole genome shotgu	505	505	55%	8.00E-142	95.31	AXNU01000037.1
Query10	3A-R	Type III-A	animalis (ATCC 51191)	Fusobacterium nucleatum subsp. vincentii ATCC 51190 Contig00029, whole geno	505	505	56%	8.00E-142	95.05	AKXI01000029.1
Query10	3A-R	Type III-A	animalis (ATCC 51191)	Fusobacterium nucleatum subsp. animalis strain P2_CP NODE_8_length_76245_	499	499	57%	4.00E-140	94.48	NPND01000008.1
Query10	3A-R	Type III-A	animalis (ATCC 51191)	Fusobacterium nucleatum subsp. animalis strain P2_LM NODE_95_length_3975_	499	499	57%	4.00E-140	94.48	NPNC01000095.1
Query10	3A-R	Type III-A	animalis (ATCC 51191)	Fusobacterium nucleatum isolate MGYG-HGUT-01459 genome assembly, contig:	429	429	56%	5.00E-119	90.77	CABKSX010000021.1
Query10	3A-R	Type III-A	animalis (ATCC 51191)	Fusobacterium nucleatum CTI-2 addTD-supercont1.28.C47, whole genome shotgu	429	429	56%	5.00E-119	90.77	AXNY01000047.1
Query10	3A-R	Type III-A	animalis (ATCC 51191)	Fusobacterium nucleatum subsp. nucleatum ChDC F316 Contig067, whole genom	429	429	57%	5.00E-119	90.55	ATKC01000067.1
Query10	3A-R	Type III-A	animalis (ATCC 51191)	Fusobacterium nucleatum subsp. nucleatum ATCC 23726 contig00070, whole gen	429	429	57%	5.00E-119	90.55	ADVK01000056.1
Query10	3A-R	Type III-A	animalis (ATCC 51191)	Fusobacterium nucleatum subsp. nucleatum strain ChDC F311 F311_contig0000(	399	399	53%	4.00E-110	90.26	LMVH01000001.1
Query10	3A-R	Type III-A	animalis (ATCC 51191)	Fusobacterium sp. HMSC064B12 Fusobacterium_spHMPREF2775-1.0_Cont52.2,	394	394	51%	2.00E-108	90.88	LTGW01000092.1
Query10	3A-R	Type III-A	animalis (ATCC 51191)	Fusobacterium nucleatum subsp. vincentii ATCC 49256 Contig0343, whole genon	394	394	51%	2.00E-108	90.64	AABF01000048.1
Query10	3A-R	Type III-A	animalis (ATCC 51191)	Fusobacterium nucleatum subsp. animalis ChDC F324 Contig092, whole genome	374	374	49%	2.00E-102	90.53	ATKD01000092.1
Query10	3A-R	Type III-A	animalis (ATCC 51191)	Fusobacterium nucleatum subsp. vincentii strain KCOM 2880 contig1, whole gen	370	370	49%	3.00E-101	90.21	PEIJ01000001.1
Query10	3A-R	Type III-A	animalis (ATCC 51191)	Fusobacterium nucleatum subsp. vincentii ChDC F8 Contig134, whole genome sh	370	370	50%	3.00E-101	89.97	ATKB01000134.1
Query10	3A-R	Type III-A	animalis (ATCC 51191)	Fusobacterium nucleatum subsp. polymorphum strain KCOM 1278 (=ChDC F313	368	368	53%	1.00E-100	88.39	NIRP01000001.1
Query10	3A-R	Type III-A	animalis (ATCC 51191)	Fusobacterium nucleatum subsp. polymorphum strain KCOM 1271 (=ChDC F305	368	368	55%	1.00E-100	87.81	NIRN01000002.1
Query10	3A-R	Type III-A	animalis (ATCC 51191)	Fusobacterium nucleatum subsp. vincentii 4_1_13 cont2.13, whole genome shotgu	366	366	52%	4.00E-100	88.52	ACDE02000013.1
Query10	3A-R	Type III-A	animalis (ATCC 51191)	Fusobacterium sp. OBRC1 ctgN280L181103C40.6313, whole genome shotgun sequ	361	361	51%	2.00E-98	89.12	JANA01000053.1
Query10	3A-R	Type III-A	animalis (ATCC 51191)	Fusobacterium sp. CM22 ctg7180000007341, whole genome shotgun sequence	357	357	53%	2.00E-97	87.74	JAQC01000021.1
Query10	3A-R	Type III-A	animalis (ATCC 51191)	Fusobacterium nucleatum subsp. polymorphum strain KCOM 1267(=ChDC F290)	355	355	50%	9.00E-97	89.2	NIRM01000002.1
Query10	3A-R	Type III-A	animalis (ATCC 51191)	Fusobacterium nucleatum subsp. polymorphum strain KCOM 1002 (=ChDC F175	355	355	56%	9.00E-97	86.77	NIRJ01000001.1
Query10	3A-R	Type III-A	animalis (ATCC 51191)	Fusobacterium nucleatum 13_3C adlCx-supercont1.1.C5, whole genome shotgun	351	351	57%	1.00E-95	86.28	JAOZ01000005.1
Query10	3A-R	Type III-A	animalis (ATCC 51191)	Fusobacterium nucleatum isolate MGYG-HGUT-01464 genome assembly, contig:	351	351	57%	1.00E-95	86.28	CABKTD010000001.1
Query10	3A-R	Type III-A	animalis (ATCC 51191)	Fusobacterium nucleatum strain 12230 NODE_2_length_191728_cov_154.899, wh	350	350	55%	4.00E-95	86.6	QKOC01000002.1
Query10	3A-R	Type III-A	animalis (ATCC 51191)	Fusobacterium nucleatum strain 12230 MIT 2016 NODE_11_length_49682_cov_2	350	350	55%	4.00E-95	86.6	NXMD01000011.1
Query10	3A-R	Type III-A	animalis (ATCC 51191)	Fusobacterium nucleatum subsp. polymorphum strain KCOM 1248 (=ChDC F113	350	350	50%	4.00E-95	88.62	NIRK01000001.1
Query10	3A-R	Type III-A	animalis (ATCC 51191)	Fusobacterium nucleatum 13-08-02 DNA, NODE_1, whole genome shotgun seque	350	350	57%	4.00E-95	86.67	BHYR01000001.1
Query10	3A-R	Type III-A	animalis (ATCC 51191)	Fusobacterium nucleatum CTI-6 addSJ-supercont1.2, whole genome shotgun seq	350	350	51%	4.00E-95	88.4	AXNV01000002.1
Query10	3A-R	Type III-A	animalis (ATCC 51191)	Fusobacterium nucleatum subsp. polymorphum ATCC 10953 Ctg90, whole genom	350	350	51%	4.00E-95	88.4	AARG01000012.1
Query10	3A-R	Type III-A	animalis (ATCC 51191)	Fusobacterium nucleatum subsp. polymorphum strain KCOM 1232 (= ChDC F37)	344	344	55%	2.00E-93	86.25	NJGI01000001.1
Query10	3A-R	Type III-A	animalis (ATCC 51191)	Fusobacterium nucleatum subsp. polymorphum strain KCOM 1274 (=ChDC F309	344	344	51%	2.00E-93	88.05	NIRO01000009.1
Query10	3A-R	Type III-A	animalis (ATCC 51191)	Fusobacterium nucleatum subsp. polymorphum strain KCOM 1257 (=ChDC F186	339	339	55%	9.00E-92	85.98	NIRL01000002.1
Query10	3A-R	Type III-A	animalis (ATCC 51191)	Fusobacterium nucleatum W1481 Contig15, whole genome shotgun sequence	335	335	56%	1.00E-90	85.89	AXUR01000015.1
Query10	3A-R	Type III-A	animalis (ATCC 51191)	Fusobacterium nucleatum CC53 contig_seqid259, whole genome shotgun sequenc	333	333	45%	4.00E-90	90	AOUL01000259.1
Query10	3A-R	Type III-A	animalis (ATCC 51191)	Fusobacterium sp. HMSC064B11 Fusobacterium_spHMPREF3051-1.0_Cont100.1	316	316	51%	4.00E-85	86.35	LTSL01000002.1
Query10	3A-R	Type III-A	animalis (ATCC 51191)	Fusobacterium canifelinum strain OH4460_COT-188 scaffold_2, whole genome sh	313	313	52%	5.00E-84	86.05	RQYY01000003.1
Query10	3A-R	Type III-A	animalis (ATCC 51191)	Fusobacterium hwasookii ChDC F206 Contig064, whole genome shotgun sequenc	270	270	50%	3.00E-71	84.14	ATKH01000064.1
Query10	3A-R	Type III-A	animalis (ATCC 51191)	Fusobacterium hwasookii ChDC F300 Contig044, whole genome shotgun sequenc	270	270	50%	3.00E-71	84.14	ATKG01000044.1
Query10	3A-R	Type III-A	animalis (ATCC 51191)	Fusobacterium hwasookii ChDC F145 Contig047, whole genome shotgun sequenc	270	270	50%	3.00E-71	84.14	ATKE01000047.1
Query10	3A-R	Type III-A	animalis (ATCC 51191)	Fusobacterium hwasookii ChDC F128 contig00001, whole genome shotgun seque	235	235	34%	1.00E-60	88	ALVD01000001.1
Query10	3A-R	Type III-A	animalis (ATCC 51191)	Fusobacterium hwasookii ChDC F174 Contig065, whole genome shotgun sequenc	230	230	34%	6.00E-59	87.82	ATKF01000065.1
Query10	3A-R	Type III-A	animalis (ATCC 51191)	TPA_asm: Fusobacterium sp. isolate UBA8871 contig_2976, whole genome shotgu	228	228	37%	2.00E-58	86.11	DPCP01000057.1

Supplementary Table S6. Lengths of PCR products from five pairs of isolates amplified by *F. nucleatum* genotyping PCR predicted from whole genome sequences

Isolates derived from CRC

			Repeat region (predicted by PROKKA)				CRISPR-associated region PCR				
Strain*	Isolate IDs	CRISPR type	start position	end position	length	annotation	forward start position	forward end position	reverse start position	reverse end position	amplicon length
A3	C-A3	typeI-B1	2268382	2270980	2599	CRISPR with 40 repeat units	2268021	2268046	2271320	2271345	3325
		typeIII-A	676375	679726	3352	CRISPR with 45 repeat units	675756	675784	680300	680324	4569
P4	C-P4	typeIII-A	511511	517284	5774	CRISPR with 77 repeat units	510891	510919	517604	517628	6738
		Others	1286749	1287977	1229	CRISPR with 19 repeat units	-	-	-	-	-
V3	C-V3	-	-	-	-	-	-	-	-	-	-
P10	C-P10	typeI-B2	878708	883340	4633	CRISPR with 70 repeat units	883585	883609	878237	878264	5373
P11	C-P11	-	-	-	-	-	-	-	-	-	-
		Others	457991	460422	2432	CRISPR with 33 repeat units	-	-	-	-	-
		typeI-B2	1453930	1455883	1954	CRISPR with 30 repeat units	1456128	1456152	1453477	1453504	2676

Isolates derived from saliva

			Repeat region (predicted by PROKKA)				CRISPR-associated region PCR				
Strain*	Isolate IDs	CRISPR type	start position	end position	length	prokka annotation	forward start position	forward end position	reverse start position	reverse end position	amplicon length
A3	S-A3	typeI-B1	612988	615586	2599	CRISPR with 40 repeat units	615922	615947	612623	612648	3325
		typeIII-A	2204290	2207641	3352	CRISPR with 45 repeat units	2208232	2208260	2203692	2203716	4569
P4	S-P4	typeIII-A	1147766	1153539	5774	CRISPR with 77 repeat units	1147146	1147174	1153859	1153883	6738
		Others	1923007	1924235	1229	CRISPR with 19 repeat units	-	-	-	-	-
V3	S-V3	-	-	-	-	-	-	-	-	-	-
P10	C-P10	typeI-B2	987052	991285	4234	CRISPR with 64 repeat units	1565819	1565843	1570765	1570792	4974
P11	S-P11	Others	48551	48775	225	CRISPR with 4 repeat units	-	-	-	-	-
		Others	368635	371066	2432	CRISPR with 33 repeat units	-	-	-	-	-
		typeI-B2	1364276	1366229	1954	CRISPR with 30 repeat units	1366474	1366498	1363823	1363850	2676

*Strain IDs are from our previous study (15). Repeat region was predicted by PROKKA. All lengths of *F. nucleatum*-genotyping PCR products derived from pair of isolates were same, excluding a pair of isolate (C-P10 and S-P10).
